# A scoping review of racism and anti-racist solutions in the health care of people who have experienced trafficking

**DOI:** 10.1371/journal.pone.0324795

**Published:** 2025-06-20

**Authors:** Brooke Bernardin, Sakshi Garg, Fajr Khan, Yewon Lee, Carrie Wade, Hanni Stoklosa

**Affiliations:** 1 Harvard T.H. Chan School of Public Health, Boston, Massachusetts, United States of America; 2 Harvard College, Cambridge, Massachusetts, United States of America; 3 Department of Emergency Medicine, Brigham and Women’s Hospital, Harvard Medical School, HEAL Trafficking, Boston, Massachusetts, United States of America; De Montfort University, UNITED KINGDOM OF GREAT BRITAIN AND NORTHERN IRELAND

## Abstract

Racial minorities are disproportionately affected by human trafficking, with African Americans making up over 30% of trafficking cases in the United States despite being only 14% of the national population. Health care providers play a crucial role in identifying and supporting survivors of trafficking as roughly two-thirds of individuals who have been trafficked encounter a healthcare professional. However, discrimination against trafficked patients of color in health care, a key setting for frontline service provision, remains unexplored. We undertook a scoping review to identify the effects of racial and ethnic discrimination in the healthcare of individuals who have experienced human trafficking, with the aim of informing anti-racist practice, treatments, interventions, and research. Following the PRISMA extension for scoping reviews (PRISMA-ScR), we identified 41 sources comprising quantitative and qualitative studies, case reports, grey literature, and text and opinion pieces. Quantitative studies indicated that there are significant gaps in service availability for Black, Indigenous, and People of Color (BIPOC) survivors in the midwestern United States. Our remaining sources suggested that healthcare provider bias and discrimination emerged through victim blaming, adultification, criminalization, or invisibility of BIPOC survivors. Racism was also perpetuated structurally through lack of culturally relevant training, fear of punishment from police or immigration enforcement, and sociocultural barriers to accessing healthcare. Furthermore, we identified best practices for future anti-trafficking efforts in health care on several levels including treatment, research, intervention design and evaluation, community partnerships, coalition-building, and political advocacy. Ultimately, healthcare providers have a unique opportunity to respond to human trafficking, but to do so effectively will require comprehensively addressing critical gaps in care for BIPOC populations across individual, interpersonal, and structural levels.

## Introduction

Human trafficking is the recruitment, transport, transfer, harboring, or receipt of individuals through force, fraud, or deception, for the purpose of labor exploitation, including sexual, physical, or domestic labor [1]. Though data collection on the extent of human trafficking is limited due to its hidden nature, its global prevalence is approximated at 27.6 million in 2021 [2]. While trafficking occurs across all races and ethnicities, African Americans are disproportionately affected, making up nearly 32% of law enforcement-tracked trafficking cases in the United States between 2008 and 2010 [3]. White, Asian, and Hispanic/Latino individuals were reported victims in 20%, 15%, and 5% of human trafficking cases during the same period.

Human trafficking has numerous health consequences, including traumatic injuries, sexually transmitted infections, and psychological symptoms. Consequently, an estimated 68% of individuals who have been trafficked come into contact with a medical professional [4]. Moreover, depression, anxiety, and post-traumatic stress disorder are prevalent among patients who have been trafficked, so they also come into contact with mental health professionals and may require long-term support [5]. Health care providers therefore play a crucial role in identifying and supporting survivors of human trafficking. However, case reports suggest that providers fail to recognize patients who have been trafficked [6,7]. This stems from a myriad of victim and health provider factors, such as fear of judgment or reprisal upon disclosure and providers’ lacking education on signs of trafficking [8,9]. Moreover, racial and ethnic biases within the anti-trafficking movement can also perpetuate stereotypes, making health care providers less likely to recognize and provide proper care to trafficking survivors of color [10,11].

Racism is a system of advantage and oppression based on race, involving individual, institutional, and structural discrimination that perpetuates inequities in power, resources, and opportunities among racial groups [12]. Racism in healthcare manifests through implicit bias, structural barriers, and direct discrimination, negatively impacting patient-provider interactions, treatment decisions, and health outcomes. Implicit biases among healthcare providers often favor White patients over minorities, leading to poorer communication and less effective care [13]. Structural racism, including state policies and socioeconomic disparities, further limits healthcare access for Black individuals while benefiting White patients [14]. These disparities contribute to profound differences in morbidity and mortality; for example, Black women are three to four times more likely to die from pregnancy-related complications than White women, and Black patients with conditions like heart disease or cancer often receive delayed or less aggressive treatment, resulting in worse outcomes [15,16]. Experiences of racism also contribute to delayed or forgone care, as seen during the COVID-19 pandemic, where minority communities faced disproportionately higher rates of severe illness and death [15]. Women of color face especially pronounced disparities in areas like perinatal and cancer care, with lower rates of timely diagnoses and appropriate treatments [17].

The existing evidence describing racism in the anti-trafficking movement, and specifically the impact of racism within health care settings, has yet to be systematically explored. In 2020, Lepianka and Colbert conducted a systematic review of literature documenting the health care needs of women who are trafficked for sex in the United States [18]. However, the publications examined lacked information regarding the race and ethnicity of patients–data that is critical to understanding their experiences of navigating the health care system. Many other reviews concerning patients who have been trafficked have focused primarily on educational resources, official protocols, or training materials available to health care providers [19–24]. Thus, although there is a wealth of literature documenting racial discrimination in health care, as well as in institutional responses to trafficking, the impact of racism on health professionals’ ability to combat trafficking remains unexplored.

Due to the lack of research on racism’s impact on health care responses to trafficking, a scoping review was undertaken. This scoping review aimed to answer the following questions: What does the existing literature say about the effect of racism in health care interactions among people who have experienced human trafficking? What potential anti-racist solutions have been identified?

## Methods

The objectives of this scoping review were to (1) characterize the existing literature on racism in health care among people who have experienced human trafficking, (2) inform anti-racist interventions and education for health care settings, and (3) identify evidence gaps and directions for future research. The question for this scoping review was formulated using the population, concept, context framework. For this review, the population is people who have experienced human trafficking, the concept is racism/anti-racism, and the context is health care.

A protocol for this review was developed by the research team using the JBI scoping review protocol template and registered on Covidence [25]. The protocol was approved by a health sciences research librarian prior to the start of the review. See S1 Appendix for the review protocol. This review was conducted in accordance with the PRISMA-ScR methodology for scoping reviews [26]. See S2 Appendix for the PRISMA-ScR checklist.

Sources were filtered to those written in English (as dictated by the language competencies of the reviewers) and to publication dates between January 2003 and January 2024, which encompasses the most active periods of human trafficking research and theory. Sources were eligible for inclusion if the following criteria were met:

1. Population: The population of interest was either people who have experienced human trafficking (as defined by the UN Palermo Protocol) or people at risk of human trafficking. Research studies with a population of healthcare providers or mixed service providers were included if the providers served as a proxy for investigating the experiences of people who experience trafficking.
2. Concept: The source investigated racial/ethnic discrimination and/or solutions to this discrimination.
3. Context: Discrimination and/or solutions occurred within a healthcare setting and/or involved healthcare providers. Studies from multiple countries and regions were included to capture a broad understanding of human trafficking and anti-racism efforts.

In order to adequately characterize this under-researched area, quantitative research studies, qualitative research studies, case reports, and text and opinion pieces were eligible for inclusion. Systematic reviews were theoretically eligible for inclusion, but none met criteria.

The most recent search was completed on April 11, 2024, and included the following databases: Ovid Medline, EMBASE, Web of Science, CINAHL, PsycINFO, Social Sciences Database, Global Health. See S3 Appendix for the full Ovid Medline search. Two key scholars in the field were contacted for additional sources, and a global listserv for anti-human trafficking research was used to solicit grey literature for the review.

Titles and abstracts were screened against the inclusion criteria by one independent reviewer, and potentially relevant sources were retrieved in full. The full text of selected citations were assessed in detail against the inclusion criteria by two independent reviewers. Any disagreements between the two reviewers were resolved through discussion, or with the lead investigator as a third reviewer.

For all included sources, data charting of basic characteristics was completed using a predetermined extraction form. This data included the geographic focus of the source, objectives, source type (quantitative research, qualitative research, case report, or text and opinion), methods and/or frameworks used, type of trafficking discussed (sex vs. labor trafficking, age/gender demographic, and domestic vs. immigrant population), racial/ethnic groups discussed, and the specific healthcare focus of the source.

For quantitative studies, key numerical findings were recorded. For qualitative research studies, case reports, and text and opinion pieces, written findings were extracted verbatim and collated in a text document. If the entire source focused on the population, concept, and context of interest – e.g., a textbook chapter about providing equitable care to BIPOC patients experiencing trafficking – all findings were extracted. For sources with a broader focus, only the findings that specifically mentioned the population, concept, and context were included. For example, in a qualitative study of trafficking survivors’ interactions with healthcare providers, only those findings that explicitly mentioned race or racism were extracted. All data charting was completed by one independent reviewer and checked by a second independent reviewer.

Critical appraisal for each source was completed by one independent reviewer and checked by a second independent reviewer. The quality of each source was assessed using the appropriate JBI critical appraisal tool [27].

The quantitative research findings were synthesized via narrative review as the studies were too heterogeneous for a meta-analysis. Written findings from qualitative research, case reports, and text and opinion pieces were synthesized via meta-aggregation. Meta-aggregation is a systematic approach to synthesizing qualitative evidence by identifying key themes, grouping them into categories, and developing overarching synthesized findings that remain close to the original data without reinterpreting it. For the meta-aggregation, two researchers open coded the data, then worked together with the lead investigator to organize the findings into categories. Findings were iteratively re-categorized and further divided into subcategories through cycles of independent analysis and group discussion. See S4 Appendix for the full meta-aggregation data.

## Results and discussion

### Source characteristics and scope of evidence

#### Overview of included sources.

**Fig 1:**
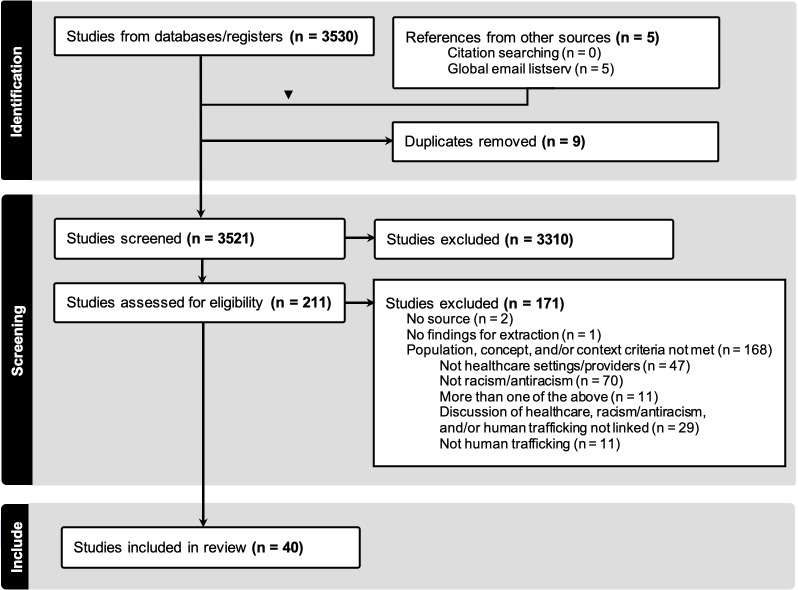
PRISMA flowchart.

A total of 3521 sources were identified for screening. The database search yielded 3525 sources, the global email listserv yielded 5 additional grey literature sources for screening, citation searching did not yield any additional sources, and 9 duplicates were removed. Of the 3521 studies screened, 211 were reviewed in full, and 41 sources were ultimately included (see Fig 1). For readability, references have been removed from this section, however, characteristics for each study are detailed in Table 1.

**Table 1:** Characteristics of included studies.

Author, Publication Date, and Title	Geographic Location/ Focus	Objective(s)	Source Type, Methods, and Framework(s)	Sex vs. Labor Trafficking	Age/Gender Demographic	Racial/Ethnic Groups Discussed	Healthcare Focus
Bounds, D (2020) – Adapting a family intervention to reduce risk factors for sexual exploitation [28]	United States: Urban Mid-Western city	To examine the relevance of an evidence-based intervention called STRIVE (support to reunite, involve, and value each other), which has been a successful family re-engagement strategy, for youth with risk factors for sexual exploitation.	Qualitative research: Focus groups with youth at risk for sexual exploitation and service providers (including APRNs and LCSWs) using the ecological model and intersectionality as the theoretical framework.	Sex trafficking	Children/ youth	Black/African American	Mental health care
Bounds, D (2023) – Strengthening Families to Disrupt Intergenerational Health InequitiesWith Adolescents at Risk for Commercial Sexual Exploitation, Substance Use, and HIV [66]	United States: Urban Mid-Western city	To share lessons learned from implementing the STRIVE intervention to aid public health practicioners working with high-risk minoritized adolescents and their families	Text and opinion: Public health practicioner recommendations based on findings from semistructured interviews and researcher experiences piloting this intervention	Sex trafficking	Children/ youth	Black/African American	Mental health care, counselors, public health practicioners
Bryant-Davis, T (2017) – Cultural Oppression and Human Trafficking: Exploring the Role of Racism and Ethnic Bias [62]	Global, but with greater focus on the United States	To describe the role of racism and ethnic bias in increasing the risk for human trafficking, outline cultural strengths relevant to the treatment of racial and ethnic minority human trafficking survivors, and recommend potential solutions to the issue of trafficking among racial and ethnic minorities.	Text and opinion: Clinical recommendations based on a critical review of the literature.	All/ Unspecified	All/ Unspecified	Racial and ethnic minorities	Mental health care, health systems broadly
Bryant-Davis, T (2017) – Women, Sex Trafficking, and the Justice System: From Victimization to Restoration (chapter in: Gender, Psychology, and Justice: The Mental Health of Women and Girls in the Legal System) [44]	United States	To examine how social dynamics related to gender, class, and race shape the U.S. justice system’s response to victims of sex trafficking and to present implications for the clinical care of trafficking survivors.	Text and opinion: Clinical recommendations based on a critical review of the literature and the first author’s clinical practice using frameworks of culturally congruent responses and a restorative approach.	Sex trafficking	Women, girls	Ethnic minorities	Mental health professionals
Bryant-Davis, T (2019) – Still We Rise: Psychotherapy for African American Girls and Women Exiting Sex Trafficking [37]	United States	To provide an overview of sex trafficking, approaches to counseling survivors of sex trafficking, dynamics of sex trafficking for African American girls and women, and approaches to counseling African American trauma survivors.	Text and opinion: Clinical recommendations based on a critical review of the literature and the author’s experience, using strengths-based, culturally congruent, and Womanist (Black feminist) frameworks.	Sex trafficking	Women, girls	Black/African American	Mental health care
Burns, C. (2021) - “We measure what we value”: Building the science to equitably respond to labor and sex trafficking [38]	United States	To discuss the need for equitable identification of people experiencing human trafficking, particularly labor trafficking.	Text and opinion: Commentary piece using an equity lens and a person-centered/trauma-informed framework.	Labor trafficking	All/ Unspecified	People of color	Emergency medicine
Claggett, K (2014) – The Role of Perceptions, Training and Experience of Psychology Students in Identifying Potential Victims of Human Trafficking [48]	United States	To determine whether graduate psychology students can identify victims of sex trafficking from fictional vignettes, and to explore factors including training, experience, and belief in stereotypes.	*Cross-sectional study: Electronic survey of graduate psychology students.	Sex trafficking	All/ Unspecified	Hispanic/ Latino, Black, Asian, Native American, Other Pacific Islander	Clinical psychology
Cook, M (2022) – Addressing Racism in the Domestic Minor Sex Trafficking of Black Girls: The Role of Public Health Critical Race Praxis [11]	United States	To describe public health critical race praxis and its application to addressing domestic minor sex trafficking (DMST) among Black girls.	Text and opinion: Critical analysis using Public Health Critical Race Praxis.	Sex trafficking	Girls	Black	Health systems, hospital programs, healthcare practitioners, and health researchers
Crane, P (2011) – Human Trafficking: What is the Role of the Health Care Provider? [60]	United States	To present a comprehensive overview of human trafficking and the related health care issues for trafficking victims, and to provide indicators, screening questions and therapeutic messages to healthcare providers as practice tools.	Text and opinion: Clinical recommendations using the stages of trafficking framework.	All/ Unspecified	All/ Unspecified	Racial/ethnic minority immigrants	Healthcare providers broadly
Davidtz, J (2022) – Cultural Considerations (chapter in: Sex trafficking: best practices for assessment and intervention) [45]	United States	To examine cultural considerations applicable to sex trafficking worldwide with an emphasis on multicultural victims detected in the United States.	Text and opinion: Clinical practice recommendations using a transcultural and trauma-informed framework.	Sex trafficking	All/ Unspecified	African American, American Indian/Alaska Native, Latin/ Hispanic, South/ Southeast Asian, and African	Healthcare professionals broadly, with a focus on mental health providers
Fukushima, A (2021) – Editorial: Anti-Trafficking Education: Sites of care, knowledge, and power [61]	Global, but with a U.S. focus	To discuss the relationship between human trafficking education and the systemic oppression of marginalized groups and to suggest ways forward for HT education.	Text and opinion: Editorial for an anti-trafficking education research collection.	All/ Unspecified	All/ Unspecified	Marginalized racial/ethnic groups, including Black, Indigenous, and Asian	Healthcare professionals and healthcare organizations
Gerassi, L (2018) – Direct Practice (chapter in: Sex trafficking and commercial sexual exploitation: Prevention, advocacy, and trauma-informed practice) [44]	Global, but with a United States focus	To provide an overview of practice theories and frameworks, to analyze the available evidence-based interventions, and to provide recommendations for customized strategies when providing services to particular subpopulations of trafficked and exploited individuals.	Text and opinion: Clinical recommendations informed by a critical review of the literature and several practice models/frameworks including harm reduction, cultural competency, strengths-based practice, and intersectionality.	Sex trafficking	All/ Unspecified	Ethnic minority immigrants and refugees, Native Americans, Black people	Health care providers generally
Gerassi, L (2020) – An Intersectional Content Analysis of Inclusive Language and Imagery Among Sex Trafficking-Related Services [20]	United States: a region of a northern, Midwestern (U.S.) state consisting of 17 counties and 3 tribes	To understand the social service landscape in a region of a Midwestern state, including the availability of sex trafficking-specific organizations, the extent to which organizations that encounter potentially sex trafficked individuals include website information about working with trafficking survivors, and the perceived inclusivity of organizations.	Quantitative research: Content analysis of 186 websites for three content areas: Sex trafficking indicators (language or symbols), LGBTQ+ Identities (language or symbols), and diverse racial and ethnic identities (language).	Sex trafficking	All/ Unspecified	Indigenous, people of color	Counseling, mental health & substance use treatment
Gerassi, L (2022) – Moving Toward Critical Consciousness and Anti-Oppressive Practice Approaches With People at Risk of Sex Trafficking: Perspectives From Social Service Providers [33]	United States: Midwest state	To understand the perceptions of racial disparities learned from sex trafficking education and the strategies used to address race and racism in social work practice.	Qualitative research: Semi-structured interviews with social workers (including counselors and therapists); uses a community-based approach framed by color-evasiveness vs. critical consciousness.	Sex trafficking	All/ Unspecified	People of color	Behavioral health providers (counselors and therapists)
Giordano, C (2008) – Practices of translation and the making of migrant subjectivities in contemporary Italy [52]	Italy	To examine the intersections between linguistic/cultural translation and forms of citizenship by focusing on the rehabilitation program for victims of human trafficking.	Case report: Ethnographic observation of a Nigerian woman who experienced trafficking and undergoes psychiatric treatment as part of her rehabilitation program.	Sex trafficking	Women	Nigerian	Psychiatry, ethno-psychiatry
Greenbaum, J (2021) – Child Labor and Sex Trafficking [63]	United States	To outline the practical aspects of working with trafficked and exploited children and to discuss rights-based, culturally sensitive, trauma-informed strategies for interacting with vulnerable patients.	Text and opinion: Clinical recommendations based on a trauma-informed, culturally sensitive, rights-based approach.	All/ Unspecified	Children/ youth	People of color	Healthcare providers who work with children
Harper, E (2013) – School-based Prevention of Commercial Sexual Exploitation: A Special Focus on the Needs of and Effective Services for At-Risk Urban African American Girls [29]	United States: low-income majority African American zip codes	To propose an evidence-based framework for school-based prevention of CSEC and to explore school-based mental health providers’ perceptions of low-income urban African American girls’ needs, as well as their perceptions and experiences related to girls’ resources and services.	*Qualitative research: In-depth interviews with mental health providers; ecological approach used to frame the research.	Sex trafficking	Girls	African American	School-based mental health providers
Koegler, E (2020) – Identifying service needs and service gaps for sexually exploited/trafficked persons in Missouri [47]	United States: Missouri	To map existing service provision within the state of Missouri and to identify geographic and/or demographic gaps in sex trafficking aftercare services by examining victims’ service needs, agencies’ ability to provide services, victim sub-populations that agencies can serve, and rural/urban availability of services.	Quantitative research: Cross-sectional electronic survey data from service providers (including healthcare providers) to human trafficking victims across Missouri.	Sex trafficking	All/ Unspecified	Tribal survivors	Medical professionals, counselors
Larson, A (2023) – Care, Self-Determination, and Safety: A Community-Centered Public Health Approach to Preventing Human Trafficking [57]	United States	To create a resource that introduces the public health approach to violence prevention and how to apply it to the prevention of human trafficking.	Text and opinion: Informational resource from an anti-trafficking organization that uses a public health approach and the social ecological model to outline drivers of trafficking and opportunities for intervention.	All/ Unspecified	All/ Unspecified	BIPOC	Healthcare and healthcare providers broadly
Lim, S (2023) – Factors Influencing Recovery and Well-Being Among Asian Survivors of International Criminal Sex Trafficking in an Urban U.S. City [29]	United States: New York City, New York	To qualitatively examine the factors at various levels of influence that impact the recovery and reintegration process of Asian criminal sex trafficking survivors in the United States from the perspective of survivors and front-line service providers.	Qualitative research: In-depth interviews with sex trafficking survivors and anti-trafficking service providers (including mental health counselors) working with East Asian clients; community-based participatory research and trauma-informed approaches.	Sex trafficking	Women	Korean, Chinese, and Korean Chinese	Mental health providers
MartinRogers, N (2020) – Missing and Murdered Indigenous Women Task Force: A Report to the Minnesota Legislature [32]	United States: Minnesota	To create recommendations for the Minnesota Commissioner of Public Safety, other state agencies, and community service organizations to reduce and end violence against Indigenous women, girls, and two spirit people.	Text and opinion with qualitative elements: Policy report informed by public hearings and community conversations (including input from survivors and family members), interviews with experts (including healthcare providers), and evidence gathering.	All/ Unspecified	Women, girls, and two spirit people	Indigenous	Healthcare broadly, mental health care, substance use treatment
Murdock, L (2022) – Youth Survivor Perspectives on Healthcare and Sex Trafficking [30]	United States: Orange County, California	To assess the perspectives of youth survivors of sex trafficking on healthcare to improve care for this population.	Qualitative research: One-on-one interviews and focus groups with people who experienced trafficking as children/ youth; grounded theory approach to analysis.	All/ Unspecified	Children/ youth	African American	Healthcare and healthcare providers broadly
Nagy, R (2020) – Human Trafficking in Northeastern Ontario: Collaborative Responses [50]	Canada: Northeastern Ontario	To present findings from participatory action research workshops including needs of trafficked women, gaps and barriers to service provision, to present a Service Mapping Toolkit grounded in Indigenous cultural practices and teachings, and in agency and self-determination, and to recommend principles for building collaborative networks.	Qualitative research: Participatory Action Research workshops with persons with lived experience of trafficking and regional service (including healthcare); research guided by indigenous and feminist methodologies (e.g., Two-Eyed seeing) and trauma-informed, violence-informed and harm reduction approaches.	All/ Unspecified	All/ Unspecified	Indigenous	Healthcare providers and health services organizations broadly, including mental health providers, addiction support services and AIDS services
Olson-Pitawanakwat, B (2021) – In Between the Missing and Murdered: The Need for Indigenous-Led Responses to Trafficking [49]	Canada: Toronto, Ontario	To provide guidance on tackling issues of colonization and structural racism at the root of gender-based violence, and to offer pathways forward at the grassroots and systemic levels using evidence from a qualitative study conducted with Indigenous trafficking survivors and professional social services providers.	Qualitative research: Interviews (indigenous storytelling format) with indigenous trafficking survivors and indigenous service providers who had worked with this population.	All/ Unspecified	Women, girls, two-spirit people, and transgender women	Indigenous	Healthcare broadly, including substance use treatment, hospital care, therapy, and indigenous healing practices
Ortega, J (2022) – Survivors of human trafficking (chapter in: Diversity in action: Case studies in cultural psychiatry) [43]	United States	To describe the structural risk factors that place people at risk of human trafficking, and to provide practice recommendations for working with diverse survivors of human trafficking.	Text and opinion: Clinical practice recommendations using a framework of trauma-informed and culturally responsive care	All/ Unspecified	All/ Unspecified	BIPOC	Clinicians and health systems broadly
Prakash, J (2022) – Human trafficking and the growing malady of disinformation [40]	United States	To describe mechanisms used by QAnon to spread disinformation, illustrate how these mechanisms adversely affect the anti-trafficking movement, and provide recommendations for the health sector to leverage education and advocacy to combat trafficking disinformation and address root causes of trafficking.	Text and opinion: Op-ed using framework of disinformation mechanisms.	All/ Unspecified	All/ Unspecified	People of color	Healthcare providers broadly
Prior, A (2023) – Forging help relationships with commercially sexually exploited youth: Perspectives of Israeli help providers [53]	Israel	To explore professional practices that help providers employ when forging relationships with commercially sexually exploited youth (CSEY) of diverse social backgrounds	Qualitative research: in-depth semi-structured interviews with Israeli help providers, analyzed using constructivist grounded theory approach	Sex trafficking	Children/ youth	Arab	Healthcare providers broadly, social workers
Rajan, I (2021) – An examination of racial minority immigrants and the trauma of human trafficking (chapter in: Trauma and racial minority immigrants: Turmoil, uncertainty, and resistance) [55]	Global, but with a U.S. focus	To contextualize trafficking from the perspective of intersectional feminism, provide key aspects of legal/mental health care barriers and challenges that trafficking survivors face, and highlight the need for helping professionals to attend to cross-cultural realities of human trafficking and culturally specific dynamics.	Text and opinion: Critical analysis piece using a framework of intersectional feminism and a resilience-based perspective to provide culturally adapted care recommendations.	All/ Unspecified	All/ Unspecified	Racial minorities	Mental health professionals
Robinson-Dooley, V (2013) – Trafficking and women of color: Hidden in plain sight (chapter in: Sex Trafficking: A Clinical Guide for Nurses) [42]	United States	To define “women of color,” discuss the vulnerabilities and risk factors that are unique to this population and require consideration in any efforts at treatment and rehabilitation, and to provide practice recommendations for social work and nursing.	Text and opinion: Clinical practice recommendations using the framework of culturally competent and multidisciplinary approaches.	All/ Unspecified	Women	People of color	Nurses and health systems broadly
Rollins, R (2017) – Who Is in Your Waiting Room? Health Care Professionals as Culturally Responsive and Trauma-Informed First Responders to Human Trafficking [58]	United States	To discuss and provide recommendations for practice-policy feedback loops that ensure evidence-based standards of care are effectively developed and applied by healthcare professionals when assisting potential victims of human trafficking.	Text and opinion: Opinion piece using a public health lens.	All/ Unspecified	All/ Unspecified	Racial and ethnic minorities	Health systems and healthcare professionals
Rothman, E (2018) – Commercial Sexual Exploitation of Children in the United States (chapter in: Violence and Trauma in the Lives of Children: Overview of Exposure) [35]	United States	To outline knowledge and methods for providers to address and intervene on sexual exploitation of children.	Text and opinion: Critical review of trauma-informed practices, with recommendations for specific racial/cultural groups.	All/ Unspecified	Children/ youth	Black, Latino, East Asian, South Asian, Native American	Healthcare providers broadly
Saewyc, E (2008) – The Hmong Youth Task Force: Evaluation of a Coalition to Address the Sexual Exploitation of Young Runaways [34]	United States: Midwest state	This paper documents the development, function, and accomplishments of the Hmong Youth Task Force (HYTF), which was created as a result of a community taking action to protect runaway youth at risk for trafficking.	Qualitative research: Semi-structured interviews with HYTF members (including healthcare providers) and review of written HYTF records; the Minnesota Public Health Wheel of Interventions Model was used as a framework to analyze HYTF functions and achievements.	Sex trafficking	Children/ youth	Hmong	Child abuse clinic
Salami’, T (2021) – Treatment considerations for foreign-born victims of human trafficking: Practical applications of an ecological framework [59]	United States	To review treatment guidelines for working with human trafficking victims using extant literature, guided by work at the Anti-Human Trafficking Program at Baylor College of Medicine in order to provide an integrative, organizing framework that can inform the management of mental health issues in foreign-born victims of human trafficking residing in the United States.	Text and opinion: Clinical practice recommendations informed by a critical review of the literature and framed using Bronfenbrenner’s Social Ecological Model.	All/ Unspecified	Adults	Racial and ethnic minorities	Behavioral Health
UK Black and Minority Ethnic Anti-Slavery Network (2021) – Promoting Racial Equality, Diversity and Inclusion: An Action Plan for the UK Modern Slavery and Human Trafficking Sector [51]	United Kingdom	To create a list of racial equality issues, recommendations, and progress indicators for the UK human trafficking response sector (NHS, DHSC and Public Health England), by looking at the health needs of victims and survivors from ethnic backgrounds.	Text and opinion: Policy report informed by policy analysis and organizational review.	All/ Unspecified	All/ Unspecified	Ethnic minorities	Human trafficking policies within health systems
Valandra (2018) – Afrocentric Intergenerational Assessment and Recovery from Sex Trafficking and Commercial Sexual Exploitation (chapter in: Social Work Practice with Survivors of Sex Trafficking and Commercial Sexual Exploitation) [39]	United States	To review research on the disproportionate representation of African Americans among sex trafficking and commercial sexual exploitation/prostitution victim/survivors; to analyze structural and individual risk factors affecting African American victim/survivors; and to promote culturally responsive, oppression-sensitive social work interventions across multiple levels of practice and policy.	Text and opinion: Critical research review and analysis using an Afrocentric intergenerational perspective.	Sex trafficking	All/ Unspecified	African American	Healthcare broadly
Villareal Armas, G (2010) – Cultural Competence in the Trauma Treatment of Thai Survivors of Modern-Day Slavery: The Relevance of Buddhist Mindfulness Practices and Healing Rituals to Transform Shame and Guilt of Forced Prostitution (chapter in: Mass Trauma and Emotional Healing Around the World) [56]	United States	This chapter examines the relevance of Buddhist mindfulness practices and healing rituals in the trauma treatment of Thai modern-day slaves.	Text and opinion: Clinical recommendations framed by the three-stage post trauma psychotherapy model.	Sex trafficking	Women	Thai	Mental health care
Walker, N (2023) – How Much is a Little Girl Worth? a Critical Qualitative Inquiry of the Systemic Dehumanization of Black Girls Through Domestic Minor Sex Trafficking (DMST) and How Schools Can Play a Pivotal Role in Prevention [35]	United States	To explore the self-identified and observed ways in which systems, especially schools, silence Black and Brown girls, as well as their recommendations for educators/schools and other systems to play a role in DMST prevention	*Qualitative research: Semi-structured interviews with Black girls and with women of color professionals who advocate for Black/ Brown girls, have worked with school systems, and are aware of DMST (includes 3 counselors, 1 therapist, and 1 pediatrician); framed by critical race feminism, intersectionality, and the Feminist Narrative Counseling Model	Sex trafficking	Girls	Black/African American	Behavioral health, pediatrics (within or in collaboration with schools)
Wallace, C (2022) – Global Perspectives on the Health and Social Impacts of Child Trafficking [54]	Global: all WHO regions and World Bank country income classifications	To obtain a cross-cultural understanding of stigma, discrimination, and barriers to healthcare, and to explore the relationship between stigmatization and health outcomes.	Qualitative research: Semi-structured interviews with child trafficking service providers (including healthcare); informed by the Health Stigma and Discrimination Framework.	Sex trafficking	Children/ youth	Ethnic minorities including Vietnamese, Hindu, and Kenyan	Physicians, nurse practitioners
Williamson, E (2020) – Featured counter-trafficking program: Love146 [36]	United States: Connecticut, U.S.	To describe the successes, challenges, and lessons learned by Love146 in facilitating U.S. survivor access to services that can improve medical and mental health outcomes.	Case report: Description and analysis of a domestic minor sex trafficking services organization.	Sex trafficking	Children/ youth	People of color	Medical and mental health services
Woo-Cater, S (2022) – Unearthing Historical Trauma to Advance Health Equity for Survivors of Human Trafficking [67]	United States	To explore how consequences of historical trauma perpetuate inequalities in communities at risk for human trafficking and assert that advancing healthcare for these patients necessitates a deep historical understanding	Text and opinion: critical analysis piece exploring historical traumas to provide education recommendations for healthcare professionals	All/ Unspecified	All/ Unspecified	People of color	Healthcare providers broadly

The greatest number of included sources (n = 24) were text and opinion. These included book chapters on healthcare practice (n = 9), published clinical recommendations (n = 4), critical analysis pieces (n = 4), opinion pieces (n = 4), task force reports with healthcare-related action items (n = 2), and an informational resource from an anti-trafficking organization (n = 1). Original research studies (n = 13) included qualitative research (n = 11), prevalence studies (n = 2), and a cross-sectional study (n = 1). Two of the qualitative research studies and the cross-sectional study were PhD dissertation research. Case reports (n = 2) included one patient case report and one organizational case report.

The earliest publication year for an included source was 2008, and the number of sources increased significantly in later years, with 9 sources published in 2022, and 4 sources published in 2023.

Quality of the sources was overall high for text and opinion sources, qualitative research, and case reports. For all text and opinion pieces, the source of opinion was clearly identified and had a standing in the field of expertise, the interests of people who experience trafficking was the central focus of the opinion, the conclusions were the result of a logical analytical process, and there were references to existing literature with any incongruence addressed. Within the included qualitative research studies, all were well-designed with congruence among their stated perspective, methodology, research question/objectives, data collection methods, representation and analysis of data, and interpretation of results. However, the included qualitative research studies frequently did not include a researcher positionality statement and did not comment upon the influence of the researcher on the research and vice versa (n = 6). Both case reports included a detailed description of the patient/organization, and they both outlined successes, challenges, and takeaways. The patient case report clearly described the patient’s condition, assessment, treatment, and post-treatment condition.

Due to notable quality issues in the quantitative research studies, we chose to include these sources while explicitly discussing their limitations..

#### Focus and scope of sources.

Most sources were geographically focused on the United States (U.S.), including original research with a U.S. population, clinical practice guidelines for U.S. healthcare providers, critical analysis focused on U.S. issues, policy analysis focused on a U.S. region, and a case report of a U.S. anti-trafficking organization [11,20, 28–48]. There were two Canadian sources, both qualitative research studies focused on indigenous populations [49,50]. One policy analysis focused on the United Kingdom [51]. One patient case report focused on Italy [52]. One qualitative study discussed ethnic minorities in Israel [53]. Another qualitative study recruited participants from multiple countries, and a few text and opinion pieces discussed global issues [54–56].

Most sources (n = 21) focused specifically on sex trafficking [11,20,28,29,31,33–37,39,44–48,52,53,55]. Only one source focused on labor trafficking [38], and the remaining sources (n = 18) included all types of trafficking [30,32,40–43,49–51,55,57–63]. Although the majority of sources (n = 19) were not focused on a specific gender or age demographic, there were 12 sources focused on women, girls, and/or gender minorities (transgender women and two-spirit people) [11,29,31,32,34,35,37,42,44,49,52,56] as well as 10 sources focused on children, youth, and/or girls [11,28–30,34–36,41,54,63].

Fifteen sources focused broadly on BIPOC or racial/ethnic minorities. 9 sources focused on Black or African American people [11,28–30,35,37,39,44], 4 sources focused on Indigenous or Native American people [20,32,49,50], 3 sources focused on East and Southeast Asian people (Korean, Chinese, Thai, and Hmong) [31,34,56], and 7 sources discussed multiple groups [41,43–45,48,54,61].

The geographic skew towards the United States may be partly explained by the inclusion of only sources written in English. However, other English-speaking countries and regions were not as well-represented. One potential explanation for this geographic bias is that U.S.-based researchers more commonly engage in explicit discussions of racism, whereas sources from other regions, especially Europe, may frame their discussions around migration, socioeconomic disparities, or other structural factors without explicitly using the term ‘racism [64,65].’ Additionally, we recognize that the conceptualization and language used to describe racial and ethnic discrimination vary across global contexts. While the U.S. has a long history of structural racism tied to Indigenous genocide, African enslavement, internment of Japanese Americans, and exploitation of immigrant labor, other countries may discuss related inequities using different frameworks, such as caste systems, colorism, or tribal affiliations, rather than explicitly naming racism. This variation in terminology and framing may have contributed to the geographic distribution of sources in this review. Future research could explore how different linguistic and cultural contexts shape discussions of racial and ethnic inequities in healthcare.

This review further highlights well-documented biases in anti-trafficking research and interventions. These biases include a focus on sex trafficking versus labor trafficking and a focus on women and children. Sources focusing on men, boys, and/or transgender men were notably absent. Black and/or African American populations were the most discussed racial/ethnic group within the review. This is partly explained by the significant bias towards sex trafficking, which is more often experienced by Black/African American people than by other ethnic groups. None of the sources focused specifically on Hispanic/Latino people, which could be due to the lack of focus on labor trafficking that more often affects this demographic group. Additionally, Black/African American and Indigenous populations might have been discussed more often in the context of racism and ethnic bias, whereas Hispanic/Latino and Asian ethnic groups may have been discussed more often in the context of immigration. Finally, the discussion of intersectionality related to LGBTQ+ identifying people of color in this review is limited to indigenous two-spirit and transgender women.

### Narrative review of quantitative findings

In this section we will summarize the quantitative findings via narrative review. Claggett (2014) examined psychology graduate students’ knowledge about the racial identity of sex trafficking victims via electronic survey [48]. In a sample (n = 52) of psychology graduate students where 6% reported prior training on the topic of human trafficking, only 7% of survey respondents correctly answered that a majority of sex trafficking victims within the U.S. are Black/African American. The majority of respondents answered Hispanic/Latino or Asian (specific proportion not reported) and 29% answered White/Caucasian. This study had several important limitations: (1) demographic data for participants was not collected, (2) the only description of sampling and recruitment is that participants were recruited via online social networks and email, (3) psychology graduate students are assumed to be future healthcare providers, (4) the association between participation in a human trafficking education program and knowledge about race was not explored, (5) the association between knowledge about race and ability to identify victims of human trafficking in case vignettes was not explored. Despite these limitations, Claggett (2014) suggests that future healthcare service providers studying psychology have misconceptions about race and trafficking risk, which may be amenable to education [48].

Gerassi et al. (2020) assessed the perceived inclusivity of social services organizations that potentially encounter sex trafficked individuals via a content analysis of social services organization websites (n = 186) in a region of a northern Midwest state [20]. Each organization’s website was examined for Spanish bilingual services, services for Native American populations, an antidiscrimination policy, and images of perceived people of color (POC). Among all counseling organizations (n = 34), 3 provided Spanish bilingual services, 1 provided services for Native American populations, none of the organizations had an antidiscrimination policy, and 7 had images of perceived POC on their websites. Among all mental health and substance use organizations (n = 20), 1 provided Spanish bilingual services, 1 provided services for Native American populations, 1 had an antidiscrimination policy, and 5 had images of perceived POC on their websites. This study had several important limitations: (1) website content does not necessarily reflect organizational culture or services, (2) organizations that are less well-resourced may not have websites, and (3) the narrow geographic scope limits generalizability. Despite these limitations, Gerassi et al. suggests that in this Midwest state, there is a lack of emphasis on services for Latino and Indigenous patients, a lack of explicit acknowledgement of antidiscrimination in practice, and a lack of POC accessing services and/or being included within advertising material [20].

Koegler et al. (2020) surveyed professionals in Missouri (including healthcare professionals) to assess perceived service needs and service availability for those who had experienced trafficking [47]. 61.5% of respondents (n = 40) indicated that their organization was able to serve “Tribal survivors of violence,” however, 38.5% (n = 25) indicated that they were unable to serve this population. This study had several important limitations: (1) there was no established sampling frame for human trafficking service providers in Missouri, (2) sampling occurred via non-probability methods (snowball sampling), (3) the narrow geographic scope limits generalizability, (4) healthcare professionals and healthcare service organizations were not analyzed separately. Despite these limitations, Koegler et al. suggests that service providers in Missouri are not adequately equipped to care for indigenous people who have experienced trafficking. Taken together, the findings from Gerassi et al. and Koegler et al. suggest that there may be significant gaps in service availability for BIPOC survivors in the mid-Western United States [20,47].

### Meta-aggregation and discussion of qualitative research, text and opinion, and case report findings

#### Racism within healthcare.

In this section we summarize qualitative research, case reports, and text and opinion pieces via meta-aggregation. Two categories - “healthcare provider bias and discrimination” and “structural and sociocultural barriers to care” – include findings that describe racism occurring within healthcare settings against people who experience trafficking.

The “healthcare provider bias and discrimination” category is focused on interpersonal racism and includes findings that describe providers’ implicit and explicit biases, stereotyping, and discriminatory actions. Within this category, three subcategories emerged: “false culpability,” “invisibility,” and “unequal care.” Selected source quotes exemplifying these subcategories can be found in Table 2.

**Table 2:** Select quotes, healthcare provider bias and discrimination.

Healthcare Provider Bias and Discrimination	
False Culpability	Many of the WOC advocates described how Black girls are often seen as older than they are and then [providers] respond to them accordingly. They shared that white girls are seen more as victims especially when trafficked by Black men, whereas Black girls are seen as running away or choosing “the life.” Journey, the LPC stated “that the world in general sees black and brown girls...as though they are older than what they are and so you’re taking away that innocence from them in a sense of expecting that they are.” [35]
	Kya [African American girl from case] might anticipate discrimination by healthcare staff, who might view her as a “troubled youth,” a “runaway,” a “fast” child (quick to engage in sexual activities) who identifies with gangs and “gets herself into trouble.” If she presents to a clinic or hospital she might be reluctant to disclose any personal information. Should staff harbor the views that Kya fears, their biases might prevent them from considering exploitation, asking appropriate questions, and offering critical services. [63]
Invisibility	Most of the white providers did not offer descriptions of racial demographics among clients and, when prompted further, typically pointed toward demographic trends of economic standing, poor family relationships, and/or runaway or homeless youth status. Only when prompted to specifically discuss the racial demographics of their caseload that they, in some cases, offered observations about the race trends of their caseloads... Indeed, providers’ attempts to avoid stereotyping, though possibly well-intentioned, only further erased race in a color-evasive narrative, which has been shown to potentially cause harm to people of color. [46]
	Black girls are not recognized as potential victims of DMST, and their victimization often goes unnoticed by those with the power to intervene. [11]
	Biases, assumptions, and stereotypes about victims from marginalized communities and immigrant populations may hinder identification. [59]
Unequal care	as our participants noted in discussions, emergency rooms are often the first or only point of contact that trafficked persons might have to seek support. Yet, sometimes there is overt racism and stigmatization of commercial sex in the delivery—or denial—of health care services. [50]
	A common theme found throughout survivors’ interviews was that of unequal treatment during their healthcare visits... Racial bias within the healthcare system impacts the care a patient receives, potentially leading to poorer patient outcomes and influencing the way individuals perceive and engage with healthcare in the future... Many survivors of human trafficking considered the care they received to have been affected by biased perceptions on the part of HCPs. [30]

“False culpability” describes how healthcare providers unjustifiably place blame on BIPOC patients who experience trafficking. Important findings within this subcategory include adultification, hypersexualization, and criminalization of this population. Adultification describes how providers view BIPOC youth as less innocent, more responsible for their actions, and therefore less deserving of help [11,35,41,46,63]. Hypersexualization and stereotypes of sexual deviancy was predominantly described as affecting women of color, with providers more likely to write off signs of sexual exploitation as promiscuity in this population [11,63]. Providers were also described as more likely to view patients of color as criminals who choose to become involved in human trafficking, drug use, and other criminalized activities [11,32,35,41,43,44,63].

“Invisibility” describes healthcare providers’ failure to see the unique challenges experienced by BIPOC patients who experience trafficking as well as a failure to address their specific needs. Sources described how stereotypes of false culpability, as outlined above, can obscure exploitation and struggles [11,35,59]. Another important finding was a “color-evasive” attitude from providers that downplayed the role of race [46].

“Unequal care” describes how biases, stereotypes, and discrimination – including issues outlined in *false culpability* and *invisibility* – lead to healthcare inequities. Findings described that BIPOC patients who experience trafficking are deterred from care, are denied care, and receive differential treatment [30,42,43,50,54,63].

The category “structural and sociocultural barriers to care” describes racism that is perpetuated through institutional norms and structural barriers to negatively impact the healthcare of BIPOC patients who have experienced trafficking. Within this category, four subcategories emerged: “carceral environments,” “BIPOC erasure,” “cultural barriers,” and “fear of arrest/deportation.” Selected source quotes exemplifying these subcategories can be found in Table 3.

**Table 3:** Select quotes, structural and sociocultural barriers to care.

Structural and Sociocultural Barriers to Care	
Carceral environments	She also encouraged that we consider how service environments can also create tension for youth when she stated that: With armed security or police there’s an adversarial relationship between many members of the African American community and the police. So what ways might we set up young people to already be tense or uncomfortable or feel criminalized as they’re coming into services for the intervention…Having an affirming or empowering environment as opposed to one where young people are initially criminalized and their behavior is being surveilled… (or) reacted to with force. I think removing that dynamic may also allow young people to feel more comfortable and express themselves. [28]
	It is important to recognize that while clinicians may view the therapeutic space as “safe,” the client may perceive that same space as “threatening.” In most treatment settings, imbalanced emphasis on “protection” of victims of trafficking results in involuntary, locked, and judiciary involvement that further interferes with efforts to “prepare” survivors by incorporating community, family, mentorship, and culturally responsive programs. The foundations of the current anti-trafficking movement in a criminal justice response have resulted in what Musto classified as “carceral protectionism.” At times trafficked people are treated as “victim-offenders,” instead of victims. This approach can mimic the pattern of control that occurred between a trafficked person and their trafficker. For example, victims of trafficking sometimes stay in shelters, but the shelters are locked down and have policies that resemble imprisonment. [43]
BIPOC Erasure	Most providers (n = 20) in our sample received trainings that, to their recollection, did not focus people of color’s disproportionate risk or vulnerability to sex trafficking. For example, Eliza, a white therapist, took two human trafficking courses at different points of her college and master’s education. When asked about whether the trainings she attended addressed the disproportionate risk of particular communities, such as people of color, in sex trafficking, she replied “Not a whole lot that I can remember.” When trainings discussed disproportionate impact, there was less of a discussion of structural racism and its impact on people of color, but rather a focus on their circumstances, such as runaway and homeless youths. [46]
	This widespread omission of labor trafficking in antitrafficking efforts reinforces structural inequities that we have seen through the United States’ history in responding to trafficking. The devaluing of people experiencing labor trafficking, who are likely to be people of color, including immigrants of color, has roots in racism and xenophobia....As emergency medicine scientists explore how we can best respond to trafficking, we must not perpetuate existing biases present in the larger antitrafficking response. [38]
	All the WOCAs stated that the fact that Black and Brown girls seem to be disproportionately targeted as the reason that people don’t care as much about the issue and specifically why especially the racial and gender disparities of DMST are not forefronted as much... [29]
Cultural barriers	Interviewees also commonly discussed that Western medical models, even if they are trauma-informed, do not provide the ongoing healing and support trafficked women need to escape prostitution and sex trafficking [32]
	…survivors also had difficulty disclosing their trafficking and current illicit massage business experiences to service providers. A provider talked about how there was no term to even describe “sex trafficking” in the Chinese language. Coupled with internalized stigma, women processed their trafficking experience in various ways, including not identifying as a survivor, reframing trafficking in a different and often more general manner (e.g., “something bad happened to me” as a euphemism for “rape” (ID009, Mental health counselor), and frequently shaming themselves for being exploited (e.g., “I brought myself into this”) (ID009, Mental health counselor). [31]
Fear of Arrest/ Deportation	Healthcare and other service providers, in efforts to support people whom they know or suspect are experiencing violence and exploitation, may feel compelled to involve law enforcement without the consent of the potential survivor. In a few states, there may even be mandatory reporting requirements for trafficking or other intersecting forms of violence, even for adults. The forms of immigration relief that are available to undocumented survivors of exploitation and violence often mandate cooperation with law enforcement, even at risk of harm to the survivor or their family. Non-consensual involvement of law enforcement has been associated with harmful safety and health outcomes and erodes trust in healthcare and other services as viable resources for people experiencing human trafficking, particularly those who are undocumented, BIPOC, LGBTQ, living in poverty, or in a criminalized economy. [57]
	Ethnic minority populations and undocumented immigrant groups, to which many human trafficking victims belong, may be particularly weary of seeking mental health services due to… fear of stigmatization, deportation, hospitalization, or incarceration… [59]
	NHS charging regulations [which require providers to determine if a patient is an “overseas visitor” for medical billing purposes] and information sharing with the Home Office negatively affects access to healthcare for unrecognised victims of modern slavery, particularly those from ethnic minority backgrounds. [51]

Findings within the “carceral environments” subcategory describe how healthcare environments potentially re-traumatize BIPOC patients who have experienced trafficking due to parallels with incarceration and/or traffickers’ methods of control [43]. This includes the presence of security or police in healthcare spaces, involuntary holds, and judicial involvement [28,43].

“BIPOC erasure” describes how deficits in healthcare providers’ training and knowledge related to human trafficking hinders their ability to adequately care for BIPOC patients. Important findings include limited training in human trafficking compared to issues that are prevalent in White populations, a lack of focus on racial dynamics and culturally relevant strategies during human trafficking trainings, and a lack of focus on labor trafficking [29,35,38,46].

“Cultural barriers” describes aspects of health systems and healthcare provision that impede access to adequate healthcare services for people of color. Findings highlighted linguistic limitations related to a lack of bilingual providers or insufficient interpreting services [31,44,52,54,60]. Sources also described a lack of culturally responsive or culture-specific services as well as an over-reliance on Western models of care [31,32,43].

“Fear of arrest/deportation” describes fears among BIPOC patients who have experienced trafficking, that engagement with the healthcare system will lead to punishment from law enforcement and judicial systems. Important findings within this subcategory included fears about healthcare providers reporting to or sharing information with police, child protective services, and/or immigration enforcement [28,51,57,59]. Additionally, sources described that these fears are driven by requirements to cooperate with law enforcement in order to access services [28,57].

Based on these findings, it is clear that racism continues to occur in healthcare settings, at multiple levels, and in ways that negatively impact the care of BIPOC patients who experience trafficking. Broadly, the evidence in this scoping review suggests two major threads within discrimination: BIPOC patients who experience trafficking are more likely to be treated as culpable or criminal, and their needs are more likely to be ignored by providers and systems. This multi-level racism leaves them with justified fears about healthcare institutions and persistent barriers to equitable care.

#### Healthcare practice recommendations.

Three categories - “broad recommendations,” “assessment,” and “treatment” – outlined best practices for providing equitable healthcare to BIPOC patients who have experienced trafficking. Findings within the “broad recommendations” category included recommendations for providers’ overall approach to the patient encounter, especially the patient-provider relationship. Subcategories within “broad recommendations” included “cultural responsiveness,” “understanding context,” “relationship-building,” “acceptance,” and “power/agency.” Selected source quotes exemplifying these subcategories can be found in Table 4.

**Table 4:** Select quotes, broad recommendations.

Broad Recommendations (for Healthcare Practice)	
Cultural responsiveness	When working with exploited children, the clinician can encounter a variety of cultural differences that impact provider-patient interactions. For example, a patient might assume a major power differential in his or her relationship with the pediatrician, expecting the clinician to dominate the conversation and make all treatment decisions. The interaction might become awkward when the physician takes a more egalitarian approach, using trauma-informed techniques to encourage the patient to ask questions, voice opinions, and participate in decisions. Gender roles and cultural taboos about sex and sexuality can be extremely important, and respecting a patient’s desire for an examiner of a particular gender is advisable whenever possible. Cultural views about sex outside of marriage, prostitution, and gender identity can have a profound effect on how a patient views his or her own situation and describes it to the clinician. Patients can have very different views regarding their life situation (fate versus divine punishment), the cause of their health problems, the acceptable ways to manifest illness, and the ways they expect clinicians to treat their health problems. Emotional distress can be manifest and interpreted in ways that are impacted by cultural views regarding mental health. Discussion of emotions might be taboo in certain cultures; some languages might lack words to describe certain emotions. [63]
	Equally important, the unique role of shame and its amplifying effects on stigma should be considered. Traffickers are very savvy and exploit these very deep-rooted cultural norms around shame as a way to exercise control over their victims; even post-trafficking, study results show that shame and internalized stigma continue to hinder survivor recovery in distinctive ways. Rather than taking a universalistic view, interventions that are cognizant of the specific cultural context of shame and stigma may show increased uptake, continuation, acceptability, and efficacy in this population. [31]
	A clinician’s familiarity with cultural and somatic idioms may aid their understanding of multicultural victims’ trauma-related symptoms. For example, study results indicated that PTSD criteria was most prevalent among women who narratively described experiences of severe sustos. Susto depicts a state of fear or fright, which could be underrated as a symptom of PTSD if one presumes the term to represent a surface-level concern. Therefore, an evaluator should not undermine the importance of semantics when presented with multicultural victim narratives that include cultural idioms. Ataques de nervios (i.e., attack of nerves) is a cultural syndrome commonly seen among Latin and Hispanic populations. Ataques de nervios may entail aggressive or violent outbursts, resembling malice intent, upon an individual’s experience of significant stress reaction. By appreciating culturally influenced beliefs and behaviors, mental health professionals enhance therapeutic approaches suitable for victims in distress. [45]
	The spiritually conservative values and morality demonstrated in African American communities make it critically necessary that practitioners frame discussions about sexual behavior within the context of promoting child and youth safety and protection to avoid any misinterpretation of “sex talk” as promoting or encouraging sex among minors. [39]
Understanding context	In order to fully understand how trafficked individuals experience their exploitation, one must understand the structural and cultural frame that trafficking occurs within. The experience of a trafficked individual sits at the nexus of multiple structural factors including socioeconomic status, family structure, race, gender, the criminal justice system, and experience of adverse childhood experiences. These factors contribute to an individual’s risk of trafficking, their ability to leave trafficking, and their recovery from the various physical and psychological sequelae of trafficking. [43]
	While Black, Indigenous, and other people of color, colonized or subjugated populations, and women and gender-nonconforming people do experience increased rates of violence, this violence is due to cultural, systemic, and structural norms, policies, and practices… Be aware of how focusing on individual-level “risk factors” can be pathologizing, might feel like prescribing future trauma, or can even lead to “pre-emptive” criminal legal responses... Risk is typically created by systemic failures rather than individual shortcomings. When we think about individual risk factors, we must always connect them back to the larger systems and policies that create those conditions. [56]
	Critically conscious and anti-oppressive approaches:... a few providers (n = 5) acknowledged the intersections between racism and classism, in accordance with a critically conscious approach, and the description of the overrepresentation of people of color among their clients. Jessica, a white counselor, suggested that “so many times women of color in our area are at poverty. Anybody that’s at poverty can be at a higher risk.”... she went on to describe this heightened vulnerability, [Women of color] are much more likely to be vulnerable because you’re a minority. There’s a lot of biases, maybe, that people have. Definitely because when we’re looking at the women of color in our area, many times they are earning the same amount. So now you’ve got a mom that has three or four children, and you’re going,“I know that she’s a prostitute.” I’m quoting, “And why is she doing that?” So we’re going, “Whoa, whoa, whoa, whoa, whoa, she is not a prostitute. She is being prostituted. Somebody’s exploiting her because she doesn’t have the income to take care of her children.” As described here, Jessica viewed the impact of structural oppression influencing the overrepresentation of women of color at risk of sex trafficking. [46]
	It is important to acknowledge the role of race when working with this population...too often we do not engage in conversations with youth regarding the implications that race has had in their risk for trafficking, their exploitation, and the services they have received. [36]
Relationship-building	The need to get to know youth and their personal experiences was coupled with the need to ensure safety. This theme was endorsed by both youth and content experts. I think that a lot of people (need to) tell their stories first…You all know we homeless so that’s why we’re here, but you don’t know our stories…like let me tell you a story first. Then, after they tell their story, then you can say, all right, so this is how STRIVE can benefit you. [28]
	Being relatable was emphasized by both experts and youth across settings. One youth articulated: You gotta relate to what people are goin’ through. You gotta be able to, I guess, like if you’re not from the hood you gotta be able to put on the shoes of somebody that is. You gotta try to see it from somebody else point of view regardless of what you’re comin’ from. [28]
	Just as survivors of other forms of trauma should be met with support and belief, persons who disclose experiences of discrimination, bias, oppression, and racism should be met with support and belief. Attempts to justify, explain, or deny the experiences of marginalized clients are sources of retraumatization. [62]
	Although participants perceived ethnic/gender similarities as facilitating relationships with girls, they indicated that ethnic or gender similarities are not necessary or sufficient for building strong relationships with girls… “Some people can relate better to the girls than others…if you’re real, they know it…I don’t care what your skin color is. If you’re coming off as a fake black person, they gonna call you fake. If you coming off as a fake white girl trying to be nice to black people, they gonna call you fake. Fake is fake…they call it out.”... “There are non- black people who have a desire to work with our populations, some more so than even our own counterparts… I think that if people’s motives are really genuine and they have an interest in learning about the population and how they can best help them and not just try to change a person because they think the person needs to be changed.” [29]
Acceptance	In addition to being prepared to meet their needs, expert panelist, Lisa poignantly reminded us of the need for a non-judgmental approach by stating, If we are not going to give them money then we need to be really careful about how we criticize the way they are getting money because it doesn’t mean they’re gonna stop getting money that way, it just means that they’re going to stop talking to us about it. [28]
	Positive regard. Participants (N = 7) reported that using positive regard… is important for working with girls this population. The positive regard code included strategies such as treating girls with respect; avoiding confrontation, derision, or sarcasm; and active listening to girls’ points of view when they display emotional or behavioral problems. For example, one participant stated, “That in-your-face kinda tactic doesn’t work a lot of times for low-income girls who have a lot of issues.” Another participant stated: They’re harder to reason with sometimes than boys… People who are more consistent with them and who show respect…they are more likely to accept advice from those people, even when they are being very stern with them. They need to have their perspective heard… they have to know that you at least respect that they have a point of view before they can hear an alternative. One participant reported that when girls are combative, that she uses a non-combative response as an opportunity to model effective communication skills. [29]
	Experts recommend using a “harm reduction” approach to working with Indigenous sex trafficking victims and survivors, both with regard to their involvement in sex work and addiction. This means “accepting sexually exploited youth and adults wherever they are in life and trying to improve their safety in non-coercive ways.” [32]
Power/ Agency	…it must be clear that the clinician is merely offering a suggestion, and, ultimately, it is the survivor’s choice to participate. [56]
	A second emergent consideration during the setting the stage phase was the high prevalence of misconceptions and alternative perspectives surrounding sexual exploitation. This is aligned with existing data that suggests that many victims of sexual exploitation do not identify as victims, nor do they label their relationships as exploitative. Content experts in the current study emphasized these misconceptions. Veronica noted the following: “you don’t want to marginalize them or turn them off to the study by initially declaring them exploited cause that’s not how they see themselves necessarily. Some will– some won’t.” Amy, a content expert colleague concurred by voicing the following perspective, “Some people will never identify as experiencing exploitation. And so changing that language would be helpful to get people engaged. [28]
	It is essential that clinicians and justice officials know there is more to culture than the experience of cultural oppression—that they recognize the cultural strengths of their clients, not just their pathologies or deficits. [37]
	Healthcare and social service providers should listen to the concerns and needs of patients and clients. This may sound simple but is often overlooked because the provider has a mental script that makes them think they already know what the patient/client needs. Many competent providers who respect people-centered care may shift into rescue narratives when they suspect human trafficking, forgetting that the values of nonjudgmental, people-centered care still apply... Do not assume you know what anyone’s concerns or needs are before they tell you…It also includes cultural humility and recognition of non-Western and Indigenous approaches to holistic healthcare, including for mental health. [57]

The “cultural responsiveness” subcategory included recommendations for tailoring one’s clinical practice to patients from racial/ethnic minority cultures. Findings centered on enhancing cultural knowledge, cultural awareness, and cultural humility to improve care for BIPOC patients who have experienced trafficking. Sources recommended enhancing cultural knowledge related to both illness framing (symptom expression, language used to express illness, healthcare expectations, cultural taboos around sexual health and mental illness) and broader culture (cultural values, norms, beliefs) to improve the quality of care [31,39,46,54,56,60,63]. The importance of providing care in the patient’s primary language was also emphasized [29]. Recommendations for practicing cultural humility included actively updating and adapting intervention strategies, accepting that cultural knowledge is never complete, asking questions rather than making assumptions, acknowledging one’s own cultural biases, and recognizing individuality within cultural groups [41,56,63].

“Understanding context” includes recommendations for developing an understanding of a patient’s life, journey, and social context, especially contextual factors that providers may not have experienced themselves. A key finding was that providers needed to better understand and recognize structural factors that impacted patients’ lives, especially structural racism, historical racial oppression, and the impact of intersectionality (poverty, gender, immigration status, etc.) [39,43,59]. A related finding was the importance of understanding “risk factors” and “vulnerability” as the result of structural forces rather than as individual attributes [11,42,43,57]. Other important recommendations included educating oneself on patients’ political and legal contexts (e.g., laws related to trafficking, political environment in a patients’ country of origin), understanding patients’ social context (family and community), and asking about patients’ individual experiences of racism, bias, and oppression [36,43,44,46,50].

“Relationship-building” includes recommendations for establishing a trusting and productive patient-provider relationship. Findings within this subcategory emphasized the importance of listening and relating to patients. Specific recommendations for listening included allowing patients space to tell their stories before providing recommendations, meeting disclosures with support and belief, and creating space to share trauma related to racial and intersectional oppression [28,31,35,44,50,57]. Recommendations pertaining to relatability included capitalizing on shared race/ethnicity, making a genuine effort to put oneself in a patients’ shoes, and caring on a more personal level [28,29,31,57,60].

“Acceptance” includes recommendations for providing care in a way that meets people wherever they are in their recovery process. Recommendations within this subcategory included using a non-judgmental approach and positive regard during patient interactions [28,29,32]. Sources also recommended harm reduction, especially with regard to criminalized forms of employment and substance use [28,31,32].

“Power/agency” includes findings that recommend shifting power and agency towards the BIPOC patient who has experienced trafficking. These recommendations included making the patient an equal partner in treatment decisions, allowing patients to self-identify, and harnessing patients’ existing strengths and resilience [28,31,42,44,56,59].

The “assessment” category includes recommended assessment strategies for meeting the needs of BIPOC patients who have experienced trafficking. Subcategories included “holistic assessment,” “cultural responsiveness,” and “safety.” Selected source quotes exemplifying these subcategories can be found in Table 5.

**Table 5:** Select quotes, assessment.

Assessment	
Holistic Assessment	The bio-psychosocial assessment is an intensive interview that gathers information about the client’s medical needs (bio), mental health concerns (psycho), and social networks and social supports (social). Recently, social workers have advocated for collecting data on clients’ faith or spiritual beliefs. An understanding of these areas are important to understanding your client as a holistic being. [42]
	To this end [helping clients form social connections] clinicians will first need to understand the social support needs of their clients and understand how these needs may materialize in a new cultural context. For example, clinicians may glean information about culturally salient community organizations, such as faith-based, LGBT, and ethnic group organizations from which their clients can derive benefit by first asking clients about their values and beliefs. Such organizations may provide a natural setting for clients to meet those with similar backgrounds and gain support. [59]
	Mental health providers should not only assess for traditionally recognized sex trafficking dynamics and consequences, but also for cultural meaning making and stigma, spirituality, social support, somatic and medical complaints, and cultural strengths. [37]
Cultural responsiveness	Another consideration is that most Southeast Asian individuals present their emotional distress as somatic complaints. However, somatic symptoms may be indicative of exposure to trauma. It would be wise for clinicians to ask questions about the client’s own insights about the presenting problems, past interventions, and expectations for treatment. [56]
	The presence of cultural mediators allows patients to speak in their mother tongue, and different interpretations of the patient’s suffering can thus circulate during the consultation. Mediators are asked to explain and clarify symptoms according to the cultural idioms of the country of origin of the patient and can also participate in the diagnosis and cure administered to the patient. Their role is to mediate between the cultural content of the patient’s idioms and the explanatory models of the doctors. This allows for a different telling and a different listening... Allowing for the migrants’ words to circulate in the clinical setting to talk about symptoms, to name suffering, is a way of creating a space of mediation where the unsaid of the migrant’s story can emerge and find its own articulation. [52]
Safety	Hospitals – sometimes people get hurt and go to the hospital and they don’t recognize the bigger context of what’s happening. They are fixing the broken arm but not asking how the break happened. They should do safety assessments. Are you safe at home? My experience is that when that’s done they do a terrible job. They are checking a box, not actually setting it up so anyone can answer in any authentic way. They are leaving the husband in the room, or they don’t ask like they’re interested. [32]
	A victim-centered approach should include an assessment of risk of ongoing threats of violence and exploitation over the course of multiple sessions. [62]

The “holistic assessment” subcategory includes recommendations for assessment topics and tools that more effectively map out the needs of BIPOC patients who have experienced trafficking. Important recommendations included assessing the extent and types of social support available to a patient, potentially using tools like the bio-psychosocial assessment, the genogram, or the eco-map [42]. Sources also recommended mapping patients’ values and beliefs, especially those related to culture and spirituality [42,59]. It was also recommended that providers assess health more holistically to include both mental and physical health [44].

Findings within the “cultural responsiveness” subcategory focused on adapting assessment to a patient’s specific culture. Recommendations included asking about a patient’s cultural frame, assessing level of acculturation, and potentially using cultural mediators during assessment [52,56,59].

The “safety” subcategory included recommendations for assessing and ensuring safety. Findings emphasized the importance of creating a safe space for assessment and doing safety assessments privately [32,42,44]. The “treatment” category includes recommended treatment strategies for providing equitable, high-quality healthcare to BIPOC patients who have experienced trafficking. This category includes three subcategories: “expanding strategies,” “holistic support,” and “cultural and linguistic adaptations.” Selected source quotes exemplifying these subcategories can be found in Table 6.

**Table 6:** Select quotes, treatment.

Treatment	
Expanding strategies	I do know that a lot of girls, and young ladies from a low socioeconomic area are not as, well, talkative and so talk therapy may not be the best for them. Teaching and giving them planning ideas…I guess you could say therapy or other modes of interventions. Social stories, using imagery, teachable moments, something visual, something that would be a little bit more meaningful for the student, to engage the student in the process. [29]
	Because sexual exploitation directly occurs to the body, the body is an integral part of the healing work that is needed. Twenty of the 23 girls at one trafficking recovery center named dance as their favorite activity of the center; scarf dancing, repetitive movement, and circle dancing was done to facilitate self-expression, confidence, comfort with the body, and social connection with the other girls. Various dance forms have emerged from and been made popular by African American girls and women. Building on this cultural resource within womanist, Black feminist, therapy can be an asset to the trauma-focused, client-centered therapeutic process that honors both the individual and their relationship with others. While Schrader and Wendland (2012) incorporated the teaching of traditional Vietnamese dance to the girls in their study, womanist therapist could incorporate teaching African dance, jazz dance, tap dance, hip hop dance, liturgical dance, or line dancing to African American girls and women. [37]
	Specific alternatives to traditional Western therapies were suggested for working with victims who emigrated from South and Southeast Asia, particularly the use of holistic healing modalities (e.g., spiritual rituals, massage therapy, acupuncture, deep breathing, meditation, herbal gardening, yoga) to assess the mind-body relationship across issues of mental health. [45]
	Culturally informed, adjunctive treatments such as expressive art therapy can support the healing process by helping survivors verbally express their trauma and make meaning of their experiences. For example, expressive writing, narrative journaling, poetry therapy, and art therapy are creative art interventions that aim to help victims transcend traumatization and thrive. Art therapy in particular has been found to promote biological as well as psychological change, to increase self-esteem, and to facilitate meaning making, the integration of right- and left-brain functions, as well as the integration of traumatic memories and experiences. Implementation of expressive art therapies, if culturally syntonic, can provide survivors with a cathartic experience. [44]
	…barriers to care and cultural stigmas associated with mental health services highlight the need for street-based interventions, particularly for survivors who are struggling with substance abuse. [37]
Holistic Support	The experience of being trafficked is mental, physical, spiritual, and emotional; thus, holistic support is necessary. Indigenous participants mentioned the importance of Grandmothers, Aunties, Uncles, and Elders in the community; of knowing the Grandfather teachings; of breaking the cycle of intergenerational trauma; of nurturing the wellness of individual, family, and community, including connections to the natural cycle; and the idea that everyone has a role in community. [50]
	Those providing brief interventions should consider maintaining an easily accessible list of multicultural referral contacts (e.g., therapists with bilingual abilities, legal aid, trauma-informed medical providers, sources for welfare services) for when a victim necessitates them. [45]
	Given the ways in which trauma and trafficking in particular can erode trust, reliable social support within one’s community context can be quite therapeutic. Among ethnically diverse trauma survivors, disclosure to one’s informal social networks, when it is met with positive reactions, can result in a decrease in traumatic stress. Culturally congruent, quality, affordable mental healthcare is not always available, particularly in low resourced countries and communities. Compassionate and well-informed emotional and instrumental support from family and friends can be a buffer from some of the negative consequences facing survivors of human trafficking. [62]
	Like the creative arts, spirituality provides resources for healing. Helping women develop their own understanding of a higher power may stimulate the creation of meaning, a sense of wholeness, and self-transformation. Acknowledging the importance of spirituality and including spiritual ideals in treatment have proven to be culturally syntonic, particularly for ethnic minority populations. Spiritual as well as religious beliefs are powerful agents of change for various ethnic minority groups, particularly African Americans, as they shape their understanding of justice, salvation, and coping with oppression. Religious beliefs can enhance an individual’s ability to cope with negative life events, and negative life events can lead to enhanced religious faith. For these reasons, the integration of spiritual and religious ideals in treatment with sex trafficked women and/or incarcerated women should be considered. [44]
	Victims of racism and ethnic bias are in danger of internalizing the negative stereotypes that exist about their communities and themselves. This internalization can result in shame and self-blame which both are related to self-harming behaviors. Positive racial and ethnic socialization on the other hand can create a buffer which allows people to create and maintain a positive view of themselves despite the negative messages with which they are bombarded. [62]
Cultural and Linguistic Adaptations	Ending the Game is a manualized group intervention developed by an African American sex trafficking survivor. Attending to the experiences of the majority of African American survivors who come to trafficking not through force or fraud but coercion, this intervention focuses on uncovering the common psychological coercion tactics that traffickers use to manipulate and exploit survivors. The intervention encourages the use of a survivor coleader, with an aim on addressing psychological coercion, disrupting the bond with the trafficker, and enhancing a sense of empowerment through healthy identity development and healthy coping strategies. The treatment consists of psychoeducation and enhancing of insight, particularly for survivors who were taught by the trafficker to conceptualize their exploitation as economic empowerment. The effects of the miseducation on the survivor’s sense of self, relationships, and future goals are explored. The outcome focus for this intervention is relapse prevention to counter the likelihood of survivors returning to traffickers based on cognitive distortions around the trafficker caring for them and/or economically empowering them. [37]
	With the goal of increasing the efficacy of prolonged exposure (PE), specifically for African Americans, Williams et al. developed a culturally adapted version of PE that incorporated race-related themes, such as racism, social support networks, faith-based coping, images of strength as potential barriers to trauma recovery, cultural mistrust, and racial and gender differences between the client and therapist, if present. Although there are no empirical investigations of culturally adapted PE for African Americans, case studies show promising outcomes. It is important to note that the Williams et al. PE adaptation was developed to target racism-related trauma. [37]
	A research study about the prostitution and trafficking of 105 Native Women produced by Minnesota Indian Women’s Sexual Assault Coalition and Prostitution Research and Education (2011) has found that... Many women expressed a need for counseling, health care, domestic violence shelters, rape crisis centers, homeless shelters, and substance abuse treatment centers that incorporated Native cultural traditions into the healing services provided [42]
	“The current medical model that is ‘trauma informed’ has been helpful helping medical organizations, nonprofits, and schools understand that trauma is a thing and we have to know how to recognize it. But, we still have a knee jerk reaction to send people to clinical environment. Sometimes that is a good thing, and sometimes it is not good because of the mistrust, lack of congruity with Indigenous values. How do we create responses that are healing informed, not trauma informed? These are traumatized people, and the best way to help them get settled and stay out of the workhouse, or get that job, stay in school, prevent diabetes … whatever it is, it’s our job to connect them with culturally meaningful and healing practices that can be normed in their families, peer groups and the organizations that serve them.” (Advocate, key informant) [32]
	Our study adds to the existing literature on recovery by identifying specific structural and cultural factors that may need to be addressed when working with Asian women. Our results indicated that these services would ideally be provided in the survivor’s primary language due to their limited English proficiency and programming would have to be flexible to accommodate women who may have full-time working hours. [31]

“Expanding strategies” includes findings that suggest incorporating treatment modalities that go beyond talk therapy and Western models of care in order to better serve BIPOC patients who have experienced trafficking. Several sources recommended expressive, experiential, or somatic therapies, including expressive arts (dance, music, visual arts, poetry), narrative therapy, mindfulness, yoga, and acupuncture [29,31,32,35,37,44,45,56]. Interventions beyond the healthcare space, were also recommended. These included street outreach, interventions led by community members, and informal opportunities for intervention in settings like education [29,32,37].

“Holistic support” includes findings that recommend a more holistic treatment lens to make care more effective for BIPOC patients who have experienced trafficking. Findings emphasized the need for more integrated care and the need to consider sociocultural factors during treatment. Sources recommended integrating treatment for physical health, mental health, and substance use, as well as connecting patients to a range of services. These services included those to meet basic needs (housing, food, healthcare), as well as education, legal support, and life skills training [28,45,50,59]. Treatment recommendations related to sociocultural factors included leveraging and enhancing social connections within family and community as part of treatment, incorporating spirituality/religion into treatment, encouraging positive racial/ethnic socialization, and exploring historical narratives of racial/ethnic resilience [39,42,44,50,59].

The “cultural and linguistic adaptations” subcategory includes recommendations for providing culture- and language-related adaptations to existing treatment modalities in order to make them more effective for BIPOC patients who have experienced trafficking. Sources recommended that cognitive behavioral therapies be adapted to a patient’s culture (e.g., with ethnic-specific materials or race-related themes) [29,37,59]. Another recommendation was that services be provided in the patient’s primary language or with cultural interpreters [31,62]. Findings also emphasized the importance of making sure that healing practices align with the patient’s cultural context, that BIPOC cultural services are made available as part of care, and that BIPOC knowledge is incorporated into services [32,37,43,49,50,56]. BIPOC leadership, and especially leadership from BIPOC survivors was another important finding [37].

The findings from this scoping review suggest that there are many promising practices for providing more equitable care to BIPOC patients who experience trafficking. While there is significant overlap with trauma-informed care principles, the findings of this review suggest a heightened need to consider issues of power, trust, and acceptance when working with this marginalized patient population. The findings support moving away from the more individualistic and narrowly biomedical tendencies of Western medicine and towards clinical practice that considers interlocking physical, social, emotional, spiritual, cultural, and community needs.

#### Anti-racist interventions for healthcare.

Two categories - “individual/interpersonal level interventions” and “health systems interventions” – discussed anti-racist interventions for healthcare settings and providers to promote racial equity when caring for people who have experienced trafficking. “Individual/interpersonal level interventions” includes findings that focus on interventions at the level of the healthcare provider to improve their interpersonal interactions with BIPOC patients who have experienced trafficking. Subcategories within “individual and interpersonal level interventions” included “critical reflection,” “education on care models,” “education on social context and oppression,” and “expanding basic education on trafficking.” Selected source quotes exemplifying these subcategories can be found in Table 7.

**Table 7:** Select quotes, individual/interpersonal level interventions.

Individual/Interpersonal Level Interventions	
Critical reflection	Cultural awareness in working with survivors of trafficking entails self-awareness and an examination of personal biases, such as those concerning race and ethnicity, and the tendency of clinicians who deny or minimize the realities of privilege and oppression to avoid topics such as racism, classism, sexism, and homophobia as they may elicit painful or difficult affect for the client and for the therapist. This self-reflection is necessary and cannot be replaced by any amount of clinical knowledge. [62]
	Oftentimes, clinicians are unaware of their own biases. Clinicians working with sex-trafficked individuals should commit to ongoing work to recognize and address their own implicit biases. Clinicians may consider starting by taking implicit association tests; tests validated to demonstrate where clinicians may have implicit biases. While more research must be done, some practices at reducing bias show some early promise. These strategies include exposing oneself to counter-stereotypical exemplars and ways to identify with the out-group. As clinicians become more aware of their own biases, they will become better equipped to work with a diverse population. [43]
	be cognizant of implicit bias and the degree to which race, ethnicity, or gender may be impacting interactions or decision making. [41]
	With all diverse populations, it is essential for practitioners to examine their own biases and personal reactions with regard to race, immigration, sexual orientation, gender, age, religion, and ability status. [46]
Education on Care Models	Further, there is a need to build professionals’ capacity to co-construct and implement creative, context- and culture-specific strategies and interventions for low-income African American girls in high need urban settings in the face of multiple barriers through explicit training. Providers will need a model for school-based mental health interventions that facilitates practitioners’ ability to collaborate with members of the local culture (e.g., teachers, other mental health professionals, and students) while constructing cultural knowledge for use during service provision. Existing models for developing and implementing participatory culture-specific school-based mental health interventions can enhance professionals’ capacity to implement cost-effective, efficacious, and sustainable collaborative mental health programs in high need urban schools. As a result of limited time and resources, strategic planning for professional development and collaborative efforts will be important. [29]
	Given the predominantly white social service workforce and overrepresentation of people of color among people who are at risk of sex trafficking, an effort to integrate critically conscious, AOP approaches, particularly among white providers, is crucial. [33]
	Overall, education on trafficking needs to incorporate the knowledge and experiences of the people most affected by trafficking and anti-trafficking, including those who endure the collateral damage caused by anti-trafficking interventions in diverse labour sectors, not only the sex trade. [61]
Education on Social Context and Oppression	For example, “In Their Shoes” is a simulated training experience for intimate partner/teen dating violence, which allows participants to walk through the choice points for economically disadvantaged survivors. The discussion guide instructs facilitators to “confront oppression in the group” by helping participants to understand that “the dynamics of oppression that support and uphold domestic violence, economic injustice, racism, xenophobia, able-bodyism, heterosexism, and all of the ways that privileged groups maintain their power and control over others.” Sex trafficking education must implement similar approaches... [46]
	Racial biases that disadvantage black CSEC survivors persist, so it is imperative that professionals who serve or treat CSE youth receive training on the consequences of systemic, institutionalized, and individual racism. [41]
	It is also vital that the history and present impacts of structural racism, anti-Blackness, colonialism, and the intersecting hierarchies of citizenship status, class, caste, gender, sexuality, and ability are acknowledged and addressed as part of the work of anti-trafficking education. [61]
	Health professionals should be trained on the diversity of people who experience trafficking, especially counter-stereotypical examples of trafficking, in order to combat structural and institutional biases that impact identification and intervention efforts for this population. [40]
Expanding Basic Education on Trafficking	…advocates also recommended training for school nurses, noting that survivors often have multiple doctors/hospital without any health professional screening for DMST by asking the right questions and knowing what to look for during the visits. According to the advocates, training for school nurses should include noticing sudden or subtle shifts in appearance over time, such as branding tattoos, bruising, and signs of addiction. [35]
	Provide training to law enforcement and emergency room staff about providing trauma-informed care for Indigenous women and girls who have experienced violence and sexual assault, including working with victims of trafficking and those who are being exploited by their own relatives or caregivers. [32]
	Providing training programs in collaboration with school personnel, first responders, and other community stakeholders about indicators of trafficking and how best to intervene will enhance clinicians’ ability to identify and treat persons who have been, or are being, trafficked. [59]
	Modern Slavery training should be included in mandatory safeguarding training for all NHS staff... All health professionals should undertake 2 yearly training on Modern slavery and human trafficking focusing on cultural intelligence, equality, diversity and inclusion... Monthly delivery of specialist events and workshops accessible to all health practitioners. Attendance based on learning needs aligning with client/user base. [51]

“Critical reflection” includes recommendations for HCPs to examine their thoughts, actions, and beliefs, and to intervene on those that are harmful to patients. The findings highlighted the need for healthcare providers to examine their personal biases and stereotypes related to race, ethnicity, intersectional identities, and privilege [29,41,43,44]. A related recommendation was for healthcare providers to reflect on how their personal values might conflict with those of their patient population [29]. The findings also emphasized the need for providers to subsequently examine the impact of biases on their patient interactions, clinical decision making, and care provision [43,44]. Sources described that this reflection process must be iterative and ongoing [43,50].

“Education on care models” includes recommendations for equipping service providers with care models that work for BIPOC patients. Findings within this subcategory highlighted models like trauma-informed care, strengths-based care, rights-based approaches, critically conscious approaches, and anti-oppressive practices [29,45,46,50,54,61]. Sources within this subcategory emphasized the importance of educating providers on care models that are based on experiential knowledge and BIPOC knowledge, in addition to teaching providers strategies that are tailored to specific cultural groups and contexts (e.g., low-income African-American girls) [23,44,61].

**“**Education on social context and oppression” includes findings that recommend healthcare providers learn about social context and systems of oppression as they apply to BIPOC patients who experience trafficking [34,35,38,40,45,54,59,61]. Key education topics recommended by sources within this subcategory included a foundational understanding of stigma, bias, discrimination, power, privilege, and oppression as well as more specific education on race, racism, intersectionality, and related concepts (i.e., colonialism and xenophobia). Sources recommended a focus on how these concepts are intertwined with trafficking.

“Expanding basic education on trafficking” emphasized the importance of expanding trafficking education to ensure that knowledgeable healthcare providers are accessible for BIPOC communities. Recommendations included mandatory trainings for all types of healthcare providers and training for school-based healthcare providers [26,29,45,59]. The importance of this training for dispelling myths and stereotypes was also emphasized [34].

The “health systems interventions” category includes anti-racist interventions at the level of health systems and healthcare institutions. Subcategories within this category included “representation and diversity,” “critical reflection,” “inclusive organizational culture,” and “inclusive policies.” Selected source quotes exemplifying these subcategories can be found in Table 8.

**Table 8:** Select quotes, health systems interventions.

Health Systems Interventions	
Representation and Diversity	Seventy-four percent of the clients Love146 has provided direct services to are children of color. As a field, it is important to acknowledge that most of the providers working with this population are white... As professionals, we must also proactively work to attract, recruit, hire, and promote staff who reflect the population we are serving. [30]
	An additional group of experts expressed their perceptions about the potential challenges associated with engaging African American youth in the STRIVE intervention: Veronica: I think you need therapists that are African American. I mean I think that would go over better with families. Again it decreases the stigma that they are working with someone who is African American and is in a healing profession. It would decrease some of the worries of being involved in a research study and distrust of researchers in general. [22]
	Some providers reported gaining valuable knowledge related to working with low-income African American students during practical experiences. For example, one provider discussed her experiences related to observing other professionals set high expectations for socioeconomically disadvantaged students: “Just to see African American women in the role…whether they were psychologists or educators, working in a low socioeconomic area, not accepting poverty, not accepting that as saying, “you can’t do it.” I didn’t see that at all. I just saw expectations all the way; and that was very much important to me, setting up expectations for students…the climate.” [23]
	Address systemic racism in all systems that interact with Indigenous women and girls (education, health care, housing, child welfare, law enforcement, criminal justice, etc.) by hiring more Indigenous staff… Create employment pipelines for Indigenous people to enter careers within the systems of education, health care, housing support, child welfare, law enforcement, and criminal justice. [26]
Critical Reflection	Mezzo-level intervention strategies also need to include the systemic review of organizational policies, practices, and processes within agencies that offer sex trafficking–specific services to identify potentially racist and oppressive practices that might act as a barrier for African Americans in accessing and engaging services. [33]
	The principle of disciplinary self-critique encourages DMST researchers and practitioners to question the norms and accepted practices in the anti–sex trafficking field that perpetuate inequity. One way to practice disciplinary self-critique is for researchers to be reflexive about existing racial power dynamics and to question who holds the power in determining the research agenda, program questions, and the interpretation of the results. [11]
	Systems looking to create programs to care for trafficked individuals may consider embarking on a Racial Equity Impact Assessment (REIA). Race Forward: The Center for Racial Justice Innovation has created a toolkit to assist organizations in undergoing a REIA. An REIA systematically evaluates how a proposed program may impact racial and ethnic groups. [37]
	It is important for anyone taking up the work of anti-trafficking education to reflect on why they or their organisation is interested in teaching about trafficking, what is given and gained by specific educational content, and what opportunities and constraints exist for ensuring an empowering and ethical experience for all involved... What does it mean to take on a ‘new’ population for care when medicine, science, law, and other institutions of biopower are also sites of control and violence? [61]
Inclusive Organizational Culture	It is also important to take action when witnessing bias and discrimination in the health-care setting, to protect patients and staff, and to eliminate any actions that can foster a culture of intolerance and stigmatization. [63]
	Create an organizational culture that does not tolerate bias and discrimination toward patients, and implement a system that allows anonymous patient or staff reporting of such behavior. [54]
Inclusive Policies	Redesign organizations policies, procedures to center survivors material needs and experiences of trauma, doing away with strict rules and policies that are pathologizing, infantilizing, and replicate patterns of power and control while excluding the most marginalized survivors. Ensure that your organization or health center has fair labor practices, pays a living wage, and is trauma-informed for staff. (Larson 2023)
	Health systems should develop policies that identify and respond to individuals who have experienced any form of human trafficking, inclusive of labor and sex trafficking. [40]
	Remove all barriers to free access to NHS health care including charging and information sharing with the Home Office for all. This is to allow access to healthcare for all victims of modern slavery and human trafficking, whether recognised or as yet unrecognised... Reversal of charging regulations and stop sharing information with Home Office… [51]
	Recruit more interpreters and make better use of existing interpreting services to help health practitioners better spot and understand the plight of potential victims of Modern Slavery and Human Trafficking. [51]

The “representation and diversity” subcategory includes recommendations for improving BIPOC representation and diversity within healthcare spaces in order to reflect the population being served and empower BIPOC communities as a whole. An important finding across sources was the need to actively recruit, hire, and promote BIPOC healthcare providers, healthcare staff, and healthcare leaders, especially those who have lived experience of trafficking [28,29,32,36,40,46]. Sources emphasized that a workforce with strong BIPOC representation and diverse perspectives facilitates care innovation, increases comfort for BIPOC patients, and helps to address institutional racism. Another finding recommended using materials that represent that cultural diversity of the patient population [44].

“Critical reflection” includes findings that recommend collective reflection on healthcare disciplines and healthcare institutions to elucidate ways in which they perpetuate racism. Topics for reflection included the biases within research frameworks and healthcare practice paradigms as well as the barriers that are perpetuated via organizational/institutional practices and policies [11,39,43,61].

“Inclusive organizational culture” includes findings that highlight the need to address factors within institutional culture that disadvantage BIPOC patients who experience trafficking. Recommendations emphasized the need for a healthcare environment that does not tolerate bias and discrimination, values differences, and supports avenues for accountability [32,35,41,54,63].

“Inclusive policies” includes findings that recommend policy changes to help healthcare institutions and health systems provide equitable care to BIPOC patients who experience trafficking. This subcategory emphasized centering the needs of BIPOC patients who experience trafficking when developing policies as well as making anti-racism and reducing barriers for BIPOC patients an explicit goal [35,57]. More specific policy recommendations included increasing access to interpreters, making culturally responsive care more widely available, including labor trafficking within health system responses, and ceasing to share information with immigration enforcement [34,44,45,57].

The findings of this scoping review show that there are potential anti-racist interventions for healthcare at multiple levels. The findings support critical assessment of bias and clinical practices at the individual level in addition to structural biases and harmful policies at the institutional level. These findings also suggest that care for BIPOC patients can be improved through healthcare provider education that is both expanded to improve access for BIPOC populations and more focused on providing care to suit this population. A final overarching action item is the need for enhancing diversity and inclusion through provider hiring, and by creating an organization whose culture and policies are intentionally structured to be inclusive of BIPOC patients and BIPOC providers.

#### Beyond healthcare.

Three categories - “research, intervention design, and evaluation,” “partnerships and coalition-building,” and “advocacy” – addressed the role of healthcare providers and healthcare institutions beyond providing clinical care. “Research, intervention design, and evaluation” includes findings related to healthcare providers in the role of researcher or program designer, as well as findings related to how research informs healthcare practice and interventions. Subcategories within this category included “research deficits,” “research impact,” “holistic interventions,” and “co-creation.” Selected source quotes exemplifying these subcategories can be found in Table 9.

**Table 9:** Select quotes, research, intervention design, and evaluation.

Research, Intervention Design, and Evaluation	
Research Deficits	Overall, very few programs or interventions for sex trafficking survivors have been formally evaluated, especially for those who were internationally trafficked, and future research efforts should aim to establish their efficacy. [31]
	The PHCRP framework encourages practitioners to critically evaluate the types of data and methods needed to assess these complex relationships. In other words, if we are to take seriously the measurement and conceptualization of intersectionality as it relates to Black girls’ vulnerability for DMST, then we must start by collecting data on race and ethnicity, in addition to data on gender and other relevant constructs. Hospital-based anti-trafficking programs that implement a multidisciplinary approach to health services are uniquely positioned to collect intersectional data encompassing both medical and social information… [11]
	Although African American girls and women are at increased risk for sex trafficking domestically, there has been little research focused on the development and provision of culturally congruent care. [37]
	Since CSE is a common risk for homeless youth, the paucity of data on the effects of CSE on the mental health of African American and sexual/gender minority children and adolescents reflects a significant scientific gap [28]
Research impact	The research partnership between the university public health nurse researcher and child abuse clinic nurse generated verifiable empirical evidence. This helped secure grant funding, provided information for community organizations and the educational video, and served as a catalyst for the local newspaper series. Results have been disseminated at national and international conferences, and were recently published in professional journals. [34]
	We also heard multiple discussions about the lack of human trafficking data specific to our region that might help secure funding. Yet, some participants also worried that lower statistics could be used against Northeastern Ontario funding applications. [50]
	There are several ways in which psychologists can expand their role to participate in advocacy practices specific to the needs of sex trafficked women in the judicial system. One of the critical steps of advocating on behalf of sex trafficked women is to investigate the interplay of trauma histories and contextual and historical factors (e.g., age, ethnicity, setting, racism, sexism), all of which have an impact on well-being. Research that examines these factors is critical to identifying the specific needs of the population. Once this information is collected, psychologists can disseminate information to leaders and partner with organizations that meet the needs of the specific interest areas. Specifically, psychologists should strive to provide and interpret data in a manner that demonstrates urgency for change, collaborate with meso-level stakeholders to develop a vision for implementing change, and develop a detailed plan for implementing the change process. Lastly, psychologists and stakeholders should be mindful of macro-systemic barriers and anticipate resistance. [44]
Holistic Interventions	The mental health system may consider whether less restrictive and culturally responsive community-based services are available for young people like Kerry. For example, a race-concordant outpatient provider delivering intensive case management services, peer mentorship services, and peer support groups focusing on distress tolerance, symptom self-management, racial injury, healthy relationships, education, and job skills may have been beneficial. [43]
	An example of Indigenous women taking up this work is the Ginoozi (“She Is Tall” in Anishinaa-bemowin) Sexual Violence Response Team (GSVRT) at the Native Women’s Resource Centre of Toronto (NWRCT, n.d.). The first of its kind in Canada, GSVRT uses a wholistic or wrap around approach to assisting women, girls, two-spirit, and trans women who are, or have been, trafficked. Helpers provide culturally safe services and preventative strategies that address the high risk of sexual violence and trauma that Indigenous women, girls, two-spirit, and trans women face....GSVRT’s blanket of services includes referrals to trauma therapy, addictions support, health care, access to Elders, traditional healers and ceremonies, housing, food, clothing, and personal care. All of these institutional supports provide a safe space and wholistic services that create a path for healing for women, girls, two-spirit and trans women who have experienced trafficking... the findings from the research that this article is founded on clearly illustrates that NWRCT is of help to the participants as it was the only agency referred to as helpful in the interviews. [49]
	Youth reinforced how we must be ready to assist in meeting their survival needs, as highlighted in the dialogue below: Interviewer: Let’s talk a little bit about young people who had to trade sex to survive. You end up sleepin’ with somebody ‘cause you needed a place to stay, you needed somethin’ to eat, you needed a ride, whatever the case might be...Could STRIVE help that person? Youth 1: Could it? If I was trading sex for a ride, would you be able to give me a free ride? If the answer is yes, then sure. If the answer is no, then I doubt it. [28]
	Given the clear need for care coordination and new services, the clinic developed a home visiting and case management service and received grant funding to support it. HYTF members have since gained additional funding, allowing establishment of empowerment groups, family support, early intervention with runaways, and multiagency collaboration. [34]
Co-Creation	In order to develop effective and appropriate strategies, the paid involvement of persons with lived experience in the collaborative network is crucial, i.e., survivor-champions, sex workers, or family members. The idea of nothing about us without us is key to developing policy frameworks and frontline supports, including peer outreach and support, that meet the needs of individuals in a manner that respects their autonomy, self-determination, and empowerment, and is without judgment. [50]
	The principle of voice, applied to DMST, is needed to address the lack of young Black female voices in DMST initiatives. Researchers need to ensure young Black females are given opportunities to participate in designing and evaluating policies and services. [11]
	Joy, the school counselor, recommended not only providing young people with support and activities, but listening to what they need in all these areas (“academically, socially, emotionally, arts”) and giving them opportunities to co-create them. [35]
	Establishing a youth advisory board for ongoing consultation, partnership, and accountability. In order to ensure that youth voices are heard, we intend to create space for youth involvement by developing a youth advisory board. Community advisory boards are foundational to community-based participatory research. Our findings suggest the need to have a continued connection with the youth we serve to ensure transparency, relatability, and cultural humility. [28]

“Research deficits” includes findings that describe how research shortcomings impact care for BIPOC patients who experience trafficking. Findings within this subcategory described that there is limited healthcare-focused research characterizing the problem of human trafficking in relation to race and intersectional identities [11,28,37]. Descriptive data, for example, is frequently not collected with race or intersectionality in mind. Sources also described a lack of research on the effectiveness of healthcare treatments for the population of BIPOC patients who experience trafficking [37]. Research measures are often not validated in BIPOC populations, and there is a lack of research on culturally congruent or expressive therapies that show promise for BIPOC patients who experience trafficking [11,59]. A final important finding is that very few human trafficking responses are evaluated with race in mind, and anti-racist healthcare interventions or anti-racist policies are rarely evaluated [31,46].

“Research impact” includes findings that touch on the broader impacts – both positive and negative – of research work. Sources emphasized that research evidence has a significant impact on program development and funding, as well as the ability to positively or negatively influence relationships with the community [28,34,44,50]. Findings highlighted the need to consider these potential impacts when undertaking research.

“Holistic interventions” includes findings that highlight the importance of healthcare-related interventions being embedded within a larger offering of services. Important services that were highlighted within this subcategory included basic needs (housing, food assistance, personal care items, transportation, and childcare), education and employment training opportunities, life skills teaching, legal assistance, case management, and home visits [28,31,37,49,62]. Sources also emphasized that holistic healthcare interventions should include access to culturally responsive care, alternative therapies, and substance use treatment [31,32,50]. Finally, sources highlighted the importance of community and social support, such as mentorship, peer support, access to community resources and community networks (e.g., indigenous community Elders), caregiver support, and community building [29,31,34,43].

“Co-creation” includes findings that emphasize the need for people who have experienced trafficking and racial oppression to be involved in research and intervention design. Sources highlighted the importance of involving those with lived experience in program design and evaluation, listening to their needs rather than assuming, centering BIPOC voices, and prioritizing experiential expertise over academic expertise [11,32,49,50,57]. Beyond listening, sources recommended providing opportunities for collaboration, reciprocal partnerships, co-creation, and leadership [28,32,37,57]. Findings also included the importance of paid involvement for those with lived experience, and the need to support education and career advancement for those with lived experience of trafficking and/or racism [50]. Another important finding was the need for accountability to people and communities who are affected by trafficking [28]. Sources recommended ongoing program evaluation by participants, consultations with key community members (e.g., Elders) and family members during program development, the use of youth and community advisory boards, and community engagement more broadly [28,37,50,51].

“Partnerships and coalition-building” includes recommendations for healthcare institutions and healthcare professionals to form partnerships and coalitions in order to better serve BIPOC patients who experience trafficking. Two subcategories emerged: “interprofessional partnerships” and “community partnerships.” Selected source quotes exemplifying these subcategories can be found in Table 10.

**Table 10:** Select quotes, partnerships and coalition-building.

Partnerships and Coalition-Building	
Interprofessional Partnerships	As part of primary and secondary prevention, it is important to involve teachers and school officials, other healthcare professionals, social workers, child services agents, and those working within the legal system. The mental health profession may be best suited to develop comprehensive treatment programs that use an ecological framework to work with victims. [59]
	Advocacy initiatives for the micro-level rehabilitation of sex trafficked women and girls may need to be innovative in order to transcend systemic barriers. These initiatives may… involve collaboration with various meso-level helping professionals and psycho-education for members of the judicial system in order to increase attention to the mental health needs of sex trafficked survivors. [44]
	Meetings also provided opportunities for learning about other organizations’ roles and existing services, improving formal and informal communication between systems and services, negotiating changes to procedures, and planning new services for sexually exploited girls. [34]
	The high rates of human trafficking within communities of color present an excellent opportunity for professionals, researchers, and policymakers who are knowledgeable about minority health disparities to contribute their expertise to human trafficking prevention and intervention strategies. [58]
Community Partnerships	If mainstream agencies are organizing the collaborative network, it is especially important to reach out to local First Nations, Friendship Centres, other Indigenous groups, and especially Elders, for inclusivity and perspective. [50]
	Additionally, mental health and justice professionals should learn how to make use of cultural community leaders, religious leaders, experts, and trained interpreters to serve as consultants and assist with the provision of culturally congruent communication and support. [44]
	Attendees concluded that a coalition had the best potential to serve the unmet needs of runaway and sexually exploited Hmong girls, because bringing together institutions and organizations that were mandated to respond to those needs, along with organizations and concerned community members who were committed to improving the lives of Hmong families and youth, could more quickly mobilize resources and make changes to institutional systems. [34]

“Interprofessional partnerships” includes findings that recommend for healthcare providers to partner with other professionals. Findings mentioned partnering with teachers and other education professionals, social workers, child services, researchers, other health professionals, the business community, legal professionals, and law enforcement [29,34,35,55,58,59,62]. Sources emphasized that these interprofessional partnerships provided opportunities for improved communication, development of collaborative policies (e.g., referral protocols), and continuity of care for people who have experienced trafficking [34,50,55,59].

“Community partnerships” includes findings that recommend for healthcare providers to partner with community members and community organizations. Sources recommended involving members and leaders of BIPOC cultural, religious, and community groups (e.g., first nations, Elders) in human trafficking responses, as well as grassroots community organizations that are involved in trafficking response already [34,43,49,50,62]. The importance of ensuring diversity and inclusion for BIPOC coalition members was also highlighted [34].

“Advocacy” includes recommendations for healthcare providers and/or healthcare institutions to become involved in advocacy work and education in order to best serve BIPOC patients who experience trafficking. Subcategories included “community engagement,” “political advocacy,” “supporting survivor advocacy,” and “community education.” Selected source quotes exemplifying these subcategories can be found in Table 11.

**Table 11:** Select quotes, advocacy.

Advocacy	
Community Engagement	Nurses and other professionals can foster grassroots efforts to combat public health problems at a variety of levels: initiating, leading, participating, and evaluating. Public health nurses have unique access and position within the community; they are viewed and respected as knowledgeable, approachable, trustworthy professionals that are altruistic in their actions related to creating community health and wellness. These assets and community rapport create the opportunity for coalition building, community organizing, and other effective public health strategies for mobilizing communities and systems change... As public health nurses, we should challenge ourselves to get involved in community organizing and coalition building as strategies for mobilizing communities and systems change. [34]
	Mezzo level… organize community stakeholders to hold public forums about mental health and substance abuse prevention; work with community stakeholders to develop comprehensive, culturally appropriate strategies to improve access to healthcare and behavioral healthcare [39]
Political Advocacy	Health professionals and health systems can advocate for policies that address social determinants of health that are also systemic vulnerabilities to human trafficking such as access to housing, social, legal, and/or employment support. Health systems should also advocate for an end to discriminatory practices against immigrants and communities of color. [40]
	Call or email your members of Congress or local elected officials to express your support for proposed policies that advance social and racial justice and public health and human rights approaches to labor exploitation or abuse of minors... Find opportunities in your professional communities (your workplace, listservs, social media, etc.) to share health equity policy demands, resources, and actions that move away from criminalization and other individual-level interventions. [57]
	Beyond creating clinical spaces that are culturally responsive, there has been a recent call to engage mental health clinicians in changing social norms and improving public policies. This work is necessary to improve the mental health of trafficked individuals, including those differentially affected by minoritized status or statuses. [43]
Supporting Survivor Advocacy	A Womanist ethic of therapeutic care also integrates empowerment that can translate into the opportunity for the formerly victimized to become role models and mentors for women who are currently rehabilitating their lives. [37]
	These [resistance] strategies can be empowering for the individual and can result in social change that reduces the occurrence of oppressive acts. These resistance strategies have included marches, rallies, voting, community organizing, protests, boycotts, filing complaints, pressing charges, pursuit of civil cases, and advocacy for just policies and procedures. For trafficking victims, this can also take the form of participating in organizations that combat human trafficking, raising awareness in the public, and voting for laws that protect victims of human trafficking. [62]
	Advocacy and community involvement may be particularly empowering for some victims. Thus, mental health professionals may wish to encourage these pursuits for clients who show interest in being active members of their community and provide support to others in need. In addition, psychological support groups for victims of trauma and human trafficking may also be suggested to build a sense of community. [59]
Education	Further, rather than “screening for trafficking,” providers can educate their patients about their rights as workers and connect them to resources because many victims may not self-identify. [40]
	Raising community awareness to prevent other young girls from running away was a key focus. The education subcommittee created a staff development video, an accompanying handout, and a comprehensive resource manual. The video included messages from school staff and local professionals, plus charts and data describing the seriousness of truancy and running away. According to one participant, “We had more data than we could put into the video... so we pulled that info into the resource manual, with prevention strategies, and what to look for and what to do when you see it... to empower them [teachers] to do as much as they can at the teaching level.” The video was shown to school district employees on a voluntary basis, and led to more referrals to the child abuse clinic. [34]
	They [health systems] can disseminate accurate information about trafficking exploitation and worker rights to counter disinformation. [40]

Findings within the “community engagement” subcategory emphasized that healthcare providers are in a unique position to mobilize communities and/or create coalitions, so recommended that providers take advantage of this position in order to advocate for BIPOC patients who experience trafficking [34,39]. “Political advocacy” includes findings that call for healthcare providers and health systems to advocate for policies to improve outcomes for BIPOC patients who experience trafficking and to prevent trafficking among this population. Sources recommended advocating on behalf of communities of color, advocating for policies that address social determinants of health, advocating for policies that ensure equal rights and protections for BIPOC, and advocating for accountability from discriminatory systems [39,40,43,45,54,57].

“Supporting survivor advocacy” includes findings that recommend for healthcare providers to encourage BIPOC patients who experience trafficking to engage in advocacy. Recommendations included supporting leadership and empowerment for this patient population, providing opportunities to become role models and mentors, and encouraging political advocacy [35,37,44,59].

“Community education” includes findings that suggest that providers engage in education to better serve BIPOC patients who experience trafficking. Recommendations included educating patients on their rights, raising community awareness about trafficking, and disseminating accurate information about trafficking [34,35,39].

Based on the findings in this section, healthcare providers have multiple avenues for preventing human trafficking and mitigating its negative effects in BIPOC populations. The findings placed major emphasis on the importance of community connections with regard to research co-creation, collaborative responses to trafficking, advocacy, and education. Survivor inclusion and leadership – in research, program design, and advocacy – is another actionable step. There is also a strong emphasis on healthcare provision being one element of a more coordinated, multi-faceted response. Finally, there are several identified areas for further research: describing the intersection of race and human trafficking as it applies to healthcare, further researching treatment effectiveness for BIPOC patients who experience trafficking, and assessing the effectiveness of anti-racist interventions within a healthcare setting.

### Summary

Quantitative studies highlighted disparities in service access for BIPOC individuals who have experienced trafficking. Qualitative research and case reports documented healthcare provider biases manifesting as victim-blaming, adultification, criminalization, and invisibility of BIPOC trafficking survivors. Structural barriers, including lack of culturally responsive training, immigration-related fears, and limited access to care, further perpetuated inequities. Text and opinion sources provided frameworks and recommendations for addressing these disparities.

Several key gaps emerged from our analysis. First, empirical research on racism’s impact within healthcare settings for trafficked individuals is limited, with much of the literature comprising opinion pieces and qualitative findings rather than large-scale quantitative studies. Second, there is a disproportionate focus on sex trafficking compared to labor trafficking, which disproportionately affects Hispanic/Latino and immigrant communities. Third, while Black and Indigenous populations were frequently discussed, there was a lack of focus on racialized subgroups such as Hispanic/Latino and Asian survivors. Fourth, healthcare provider training programs often fail to address structural racism explicitly, limiting their effectiveness in reducing bias.

Future studies should prioritize empirical investigations into the impact of racism on healthcare interactions for trafficked individuals, including mixed-methods and longitudinal research. Further, studies should explore labor trafficking survivors’ healthcare experiences to ensure inclusivity in anti-trafficking efforts. Additionally, research should evaluate the effectiveness of anti-racist interventions, such as implicit bias training and trauma-informed, culturally responsive care models, in improving health outcomes for BIPOC survivors. Addressing these gaps will be crucial for informing policy changes, provider education, and systemic reforms to promote equitable healthcare for trafficking survivors.

### Limitations

The findings of this scoping review are limited by the biases in the available evidence. As described previously, the sources included in this review tended to geographically focus on the United States, to focus on sex trafficking more often than labor trafficking, and to emphasize the needs of women and children. Thus, the findings of this review will be more applicable to these groups and their needs. Additionally, the use of a methodology that relies primarily on academic databases tends to represent patient experiences and needs through the lens of clinical researchers’ interpretations and clinical providers’ experiences. This issue is exacerbated by the fact that several included qualitative and quantitative research sources relied on healthcare providers’ experiences with patients and providers’ assessments of patient needs as a proxy for patients’ healthcare experiences and needs. Another important limitation of this review is that sources which alluded to race or ethnicity without explicitly mentioning racism, ethnic discrimination, or healthcare for a specific racial/ethnic group, could not be included in the review. This may have limited the scope of the evidence, especially for groups whose healthcare barriers tend to be framed through language and immigration issues.

## Conclusion

This scoping review aimed to answer the following questions: What does the existing literature say about the effect of racism in health care interactions among people who have experienced human trafficking? What potential anti-racist solutions have been identified? Based on this literature review, health care has the opportunity and responsibility to act, using the evidence at hand to address the gaps in care for BIPOC populations. These changes must occur synergistically at all levels, including the individual, interpersonal and structural levels to promote anti-racism and health equity. Furthermore, health professionals who are engaged in anti-trafficking work can be guided by the best-practices outlined in this literature review including through research, intervention design, and evaluation, partnerships & coalition-building, and advocacy. Finally, research, guided by lived experience experts, using community based participatory approaches needs to further advance our understanding of these critical questions for the diversity of populations of those experiencing human trafficking who interact with the health care setting.

## Supporting information

S1 Appendix Review protocol. (PDF)

S2 Appendix PRISMA-ScR checklist. (PDF)

S3 Appendix Example search strategy for ovid medline. (PDF)

S4 Appendix Meta-aggregation data for qualitative research, case reports, and text and opinion pieces. (PDF)
