# Supplementary material for: A scoping review of racism and anti-racist solutions in the health care of people who have experienced trafficking
Source: PLoS One. 2025 Jun 20;20(6):e0324795. doi: 10.1371/journal.pone.0324795 (PMC12180640; doi:10.1371/journal.pone.0324795)
Supplement: S1 Appendix — (PDF) [file pone.0324795.s001.pdf]

S1 Appendix. Review Protocol.

The following protocol was created using the JBI protocol template for scoping reviews.

A Scoping Review of Racism and Anti-Racist Solutions in the Health Care of People who have Experienced Trafficking

Introduction

Human trafficking is the recruitment, transport, transfer, harbouring, or receipt of individuals through force, fraud, or deception, for the purpose of labor exploitation, including sexual, physical, or domestic labor (UNODC). Though data collection on the extent of human trafficking is limited due to its hidden nature, the global prevalence of human trafficking is approximated at 27.6 million (ILO & WalkFree). While trafficking occurs across all races and ethnicities, African-Americans are disproportionately affected, making up nearly 40% of trafficking cases in the United States (BOJ 2011). Human trafficking has numerous health consequences, including traumatic injuries, sexually transmitted infections, and psychological symptoms. Consequently, an estimated 68% of individuals who have been trafficked come into contact with a medical professional (Chisolm-Straker). Health care providers therefore play a crucial role in identifying and supporting survivors of human trafficking. However, case reports indicate that providers consistently fail to recognize patients who have been trafficked (Lederer, Baldwin). This stems from both victims' fear of self-identification and, more importantly, from providers' lack of awareness and education on signs of trafficking. Racial and ethnic biases within the anti-trafficking movement can also perpetuate stereotypes, making health care providers less likely to recognize and provide proper care to trafficking survivors of color.

The existing evidence describing racism in the anti-trafficking movement, and specifically the impact of racism within health care settings, has yet to be systematically explored. In 2020, Lepianka and Colbert conducted a systematic review of literature documenting the health care needs of women who are trafficked for sex in the United States. However, the publications examined lacked information regarding the race and ethnicity of patients–data that is critical to understanding their experiences of navigating the health care system. Many other reviews concerning patients who have been trafficked focus primarily on educational resources, official protocols, or training materials available to health care providers (Hemmings 2016, Ricks 2022, Gerassi & Skinkis 2020, Talbott et al. 2020, Talbott et al. 2022, Ahn 2013, Hainaut et al. 2022). Therefore, there is a critical need to examine patient perspectives to better understand barriers in access to quality care. A 2020 qualitative study

conducted with adult women impacted by commercial sexual exploitation found that Black women perceived preferential treatment given to White women when accessing social services (Gerassi 2020). This study did not include social service providers within health care settings. Thus, although there is a wealth of literature documenting racial discrimination in health care, as well as in institutional responses to trafficking, the impact of racism on health care professionals' ability to combat trafficking remains unexplored.

A scoping review methodology was used to inform this project. As little is currently known about the impact of racism on the health care response to trafficking, this approach was chosen as it is beneficial for understanding the extent of the current literature base and guiding future research.

The objectives of this scoping review are to (1) characterize the existing literature on racism in health care among people who have experienced human trafficking, (2) inform anti-racist interventions and education for health care settings, and (3) identify evidence gaps and directions for future research.

Review question

The question for this scoping review was formulated using the population, concept, context framework. For this review, the population is people who have experienced human trafficking, the concept is racism/anti-racism, and the context is health care. This scoping review aims to answer the following questions: What does the existing literature say about the effect of racism in health care interactions among people who have experienced human trafficking? What potential anti-racist solutions have been identified?

Eligibility criteria

1. Population: people who have experienced human trafficking.

The trafficking experiences of the study population may be ongoing at the time of the study or they may have occurred prior to the study. This review will adhere to the international definition of human trafficking as outlined in Section I Article 4 of the United Nations Palermo Protocol.

> (a) "Trafficking in persons" shall mean the recruitment, transportation, transfer, harbouring or receipt of persons, by means of the threat or use of force or other forms of coercion, of abduction, of fraud, of deception, of the abuse of power or of a position of vulnerability or of the giving or receiving of payments or benefits to achieve the consent of a person having control over another person, for the purpose of exploitation. Exploitation shall include, at a minimum, the

exploitation of the prostitution of others or other forms of sexual exploitation, forced labour or services, slavery or practices similar to slavery, servitude or the removal of organs;

(b) The consent of a victim of trafficking in persons to the intended exploitation set forth in subparagraph (a) of this article shall be irrelevant where any of the means set forth in subparagraph (a) have been used;

(c) The recruitment, transportation, transfer, harbouring or receipt of a child for the purpose of exploitation shall be considered "trafficking in persons" even if this does not involve any of the means set forth in subparagraph (a) of this article;

(d) "Child" shall mean any person under eighteen years of age.

The following is a non-exhaustive list of the types of sources of evidence that meet the population criterion:

- Research studies with quantitative data and/or qualitative description ***from*** **people who have experienced human trafficking**.
    - e.g. % of people who have experienced trafficking who report a microaggression during an interaction with a health care provider
    - e.g. racial/ethnic discrimination during health care interactions, as described by people who have experienced human trafficking
- Research studies with quantitative data and/or qualitative description ***from*** **health care providers** concerning human trafficking survivors.
    - e.g. % of health care providers who believe that race/ethnicity is a risk factor for human trafficking
    - e.g. an exploration of racial biases present in health care providers' descriptions of people who have experienced trafficking
- Text and opinion pieces that focus on people who have experienced human trafficking.
    - e.g. a theory-driven discussion of racism against people experiencing trafficking, specifically within a health care setting
- Program evaluations with outcomes related to quality of care for people experiencing human trafficking.
    - e.g. an evaluation of anti-racist health care interventions, specifically with outcomes related to the care of people experiencing human trafficking.

2. Concept: racism/anti-racism

Sources of evidence should address racism and/or anti-racism to be included in the review.

For this review, **racism** will encompass discrimination, prejudice, stereotyping, or bias directed against one or more racial/ethnic subgroups of people who have experienced human trafficking. These subgroups include, but are not limited to:

- Black or African American people
- Hispanic or Latina/Latino people
- Indigenous, native, or aboriginal people
- Migrants, asylum seekers, refugees and immigrants
- Roma people

Religious groups will **not** be considered a racial/ethnic subgroup.

For this review, **anti-racism** will encompass any solutions to reduce racism as defined above.

The following is a non-exhaustive list of the types of sources of evidence that meet the concept criterion:

- Research studies with quantitative data and/or qualitative description of racism as reported by people who have experienced human trafficking.
- Research studies with quantitative data and/or qualitative description of racism against people who have experienced human trafficking as reported by health care providers.
- Text and opinion pieces that discuss racism in health care settings directed against people who have experienced human trafficking.
- Thought pieces that discuss anti-racist solutions for health care settings, with the aim of addressing racism against people who are experiencing trafficking.
- Research studies that evaluate anti-racist solutions for health care settings, with the aim of addressing racial/ethnic racism against people who are experiencing trafficking.

3. Context: health care, including behavioral health

Sources of evidence will be eligible for inclusion if they examine interactions in health care settings or evaluate programs in health care settings. These health care settings include, but are not limited to:

- Inpatient care
- Outpatient/ambulatory care - private practice, community health centers

- Emergency care
- Urgent care
- Telehealth
- Mental health care
- Substance use disorder treatment
- Health services in educational settings
- Inpatient rehabilitation
- Nursing homes and assisted living facilities
- Home health care
- Pharmacies
- Laboratory services

Sources of evidence will also be eligible if they examine health care providers working in the above settings, including, but not limited to:

- Physicians
- Nurses
- Nurse practitioners
- Physician assistants
- Pharmacists
- Dentists
- Physical/occupational therapists
- Social workers in health care settings
- Dieticians in health care settings
- Midwives

Sources of evidence that discuss health care interactions with people other than health care providers, such as receptionists or medical billing staff, are also eligible for inclusion in the review.

Text or opinion pieces that discuss racism and/or anti-racist solutions in these types of settings *or* for these types of providers are eligible for inclusion in the review.

4. Published in English

Sources of evidence for this review will be in English as dictated by the language competencies of the reviewers.

5. Published from January 2003 to January 2024

This broad date range encompasses the most active period of human trafficking research

and academic discussion. A broad range has been chosen due to the limited evidence available.

Types of Sources

This scoping review will consider both experimental and quasi-experimental study designs including randomized controlled trials, non-randomized controlled trials, before and after studies and interrupted time-series studies. In addition, analytical observational studies including prospective and retrospective cohort studies, case-control studies and analytical cross-sectional studies will be considered for inclusion. This review will also consider descriptive observational study designs including case series, individual case reports and descriptive cross-sectional studies for inclusion.

Qualitative studies will also be considered that focus on qualitative data including, but not limited to, designs such as phenomenology, grounded theory, ethnography, qualitative description, action research and feminist research.

Text and opinion papers will also be considered for inclusion in this scoping review.

Methods

The proposed scoping review will be conducted in accordance with the PRISMA-ScR methodology for scoping reviews.

Search strategy

The search strategy will aim to locate both published and unpublished studies. An initial search of Web of Science, PubMed, and CINAHL was undertaken to identify articles on the topic of human trafficking, racism/anti-racism, and health care as well as systematic reviews that included human trafficking, racism, anti-racism, or health care as search terms. The text words contained in the titles and abstracts of relevant articles, the index terms used to describe the articles, and the search terms used in systematic reviews on related topics were used to develop a full search strategy for Web of Science, PubMed, CINAHL, PsychINFO, and MEDLINE. The search strategy will be adapted for each included database. Additional databases and gray literature sources may be added as determined by the research team. The reference list of all included sources of evidence will be screened for additional studies.

Study/Source of Evidence selection

Following the search, all identified sources of evidence will be collated and uploaded into Covidence and duplicates removed. Titles and abstracts will then be screened by one independent reviewer for assessment against the inclusion criteria for the review. Potentially

relevant sources will be retrieved in full and their citation details imported into the Zotero. The full text of selected citations will be assessed in detail against the inclusion criteria by two independent reviewers. Reasons for exclusion of sources of evidence at full text that do not meet the inclusion criteria will be recorded and reported in the scoping review. Any disagreements that arise between the two reviewers at each stage of the selection process will be resolved through discussion, or with the lead investigator as a third reviewer. The results of the search and the study inclusion process will be reported in full in the final scoping review and presented in a Preferred Reporting Items for Systematic Reviews and Meta-analyses extension for scoping review (PRISMA-ScR) flow diagram.

Data Extraction

Data will be extracted from papers included in the scoping review by two independent reviewers. Due to the expected heterogeneity of the sources of evidence, a single data extraction form cannot be developed in advance of evidence selection. It is anticipated that the data extracted will include specific details about type(s) of trafficking experienced, demographic characteristics of the participants, racial/ethnic groups discussed, type of health care setting or health care provider, themes identified, theories cited, and other outcome data reported by the sources of evidence.

Any disagreements that arise between the reviewers will be resolved through discussion, or with an additional reviewer. If appropriate, authors of papers will be contacted to request missing or additional data, where required.
