# Supplementary material for: A scoping review of racism and anti-racist solutions in the health care of people who have experienced trafficking
Source: PLoS One. 2025 Jun 20;20(6):e0324795. doi: 10.1371/journal.pone.0324795 (PMC12180640; doi:10.1371/journal.pone.0324795)
Supplement: S3 Appendix — (PDF) [file pone.0324795.s003.pdf]

S3 Appendix. Example Search Strategy for Ovid Medline.

Ovid Medline Search April 11, 2024

1. (((human* or sex* or labo?r or victim* or survivor* or child*) adj2 (traffick* or slave* or exploitation OR enslave*)) or (traffick* adj2 (worker* OR people or person? or m#n or wom#n or labo?r)) or (("transactional sex" or "sex work*" or prostitut* OR "commercial sex*") adj2 (minor? or child OR children OR childhood OR youth* OR teen* OR adolescen*)) or ("domestic work*" or "domestic servi*" or "forced labo?r" or "domestic slave*" or "modern slave*" OR DMST OR CSEC)).ti,ab,kf,kw. OR exp Enslavement/ or exp Human Trafficking/ or exp Enslaved Persons/ or (exp Sex Work/ AND (exp Child/ OR exp Adolescent/))

2. (anti-oppress* OR race OR racis* OR racial* OR anti-racis* OR antiracis* OR equit* OR inequit* OR ethnic* OR Black* OR "african american*" OR afro* OR hispanic* OR latino* OR latinx OR latina* OR indigenous OR "native american*" OR "american indian*" OR "first nation*" OR aborigin* OR amerindian* OR "pacific islander*" OR arab? OR arabic* OR persian* OR ((people OR person? OR m?n OR wom?n) adj1 colo?r) OR asian* OR roma? OR roman? OR "white suprem*" OR "white complicit*" OR immigrant* OR migrant* OR emigrant* OR foreigner* OR xenophobi* OR "asylum seek*" OR refugee* OR (cultural* adj3 (competen* OR proficien* OR humil* OR sensit*)) OR microaggress* OR anti-arab* OR anti-black* OR anti-indigenous* OR anti-brown* OR anti-asian* OR bigot* OR whiteness OR intersectional* OR minority OR minorities OR minoritized OR ((prejudic* or stereotyp* OR discrimnat* OR bias* OR stigma*) adj3 (race OR racial* OR racis* OR Black OR ethnic* OR "african american" OR anti-black OR antiblack OR colo?r*))).ti,ab,kf,kw. OR (exp Racial Groups/ OR exp Racial Factors/ OR exp Race Relations/ OR Prejudice/ OR exp Racism/ OR exp Bias, Implicit/ OR Social Justice/ OR exp Civil Rights/ OR Human Rights/ OR exp Cultural Competency/ OR exp Population Groups/ OR exp Ethnicity/ OR exp "Emigrants and immigrants"/ OR exp "Emigration and immigration"/ OR Xenophobia/ OR exp Vulnerable Populations/)

3. (hospital* OR clinic* OR inpatient* OR outpatient* OR "public health" OR "global health" OR ((medical OR health* OR care) adj2 (facilit* OR center OR centre OR provider OR service* OR office$ OR practitioner* OR urgent OR ambulatory)) OR (emergency adj2 (room OR medic* OR care* OR center OR centre)) OR "waiting room*" OR "social work*" OR (mental adj2 health*) OR psycholog* OR psyciatr* OR therap* OR ((victim* OR surviv* OR social*) adj2 (support* OR advoca* OR welfare OR service*)) OR p?ediatric* OR gyn?ecol* OR obstetric* OR doctor* OR physician* OR counsel* OR nurse? OR midiwi* OR "primary care" OR "general practi*" OR "family practi*" OR "internal medicin*" OR dentist* OR pharmacist OR pharmacy OR abortion OR pregnan* OR contracept* OR "family planning" OR prenatal OR neonatal OR postnatal OR postpartum OR safeguard* OR intervention* OR treatment* OR treating OR "trauma informed" OR "waiting room*" OR rehab*).ti,ab,kf,kw. OR (Health Facilities/ OR exp Academic Medical Centers/ OR exp Ambulatory Care Facilities/ OR exp Birthing Centers/ OR exp Dental Facilities/ OR exp Hospital Units/ OR exp Hospitals/ OR exp Pharmacies OR exp Physicians' Offices/ OR exp Waiting Rooms/ OR exp Health Personnel/ OR exp Public Health/ OR exp Health Services Accessibility/)

4. AND/1-3

5. Filter to publication years and language.
