# Supplementary material for: A scoping review of racism and anti-racist solutions in the health care of people who have experienced trafficking
Source: PLoS One. 2025 Jun 20;20(6):e0324795. doi: 10.1371/journal.pone.0324795 (PMC12180640; doi:10.1371/journal.pone.0324795)
Supplement: S4 Appendix — (PDF) [file pone.0324795.s004.pdf]

S4 Appendix. Meta-aggregation Data for Qualitative Research, Case Reports, and Text and Opinion Pieces.

Racism Within Healthcare

Healthcare Provider Bias & Discrimination

Subcategory: (False) Culpability

Implicit and explicit racism, other biases, and victim shaming in... health care... deter Indigenous women, girls, and two spirit people and their communities from seeking help when they need it. Shaming women who have been trafficked – whether for the trafficking itself or for the drug use that often accompanies trafficking – leads to ongoing traumatization. (MMIW)

Kya [African American girl from case] might anticipate discrimination by healthcare staff, who might view her as a “troubled youth,” a “runaway,” a “fast” child (quick to engage in sexual activities) who identifies with gangs and “gets herself into trouble.” If she presents to a clinic or hospital she might be reluctant to disclose any personal information. Should staff harbor the views that Kya fears, their biases might prevent them from considering exploitation, asking appropriate questions, and offering critical services. (Greenbaum 2021)

"...it got to a serious point where she even called the place where I was at because she wanted to take my baby. Like thinking that my daughter wasn't in a safe environment because… definitely one racially profiled my child's father being African American, being dark-skinned…you know. Knowing of my history of trafficking." (Murdock 2022)

"Many of the WOC advocates described how Black girls are often seen as older than they are and then [providers] respond to them accordingly. They shared that white girls are seen more as victims especially when trafficked by Black men, whereas Black girls are seen as running away or choosing “the life.” Journey, the LPC stated “that the world in general sees black and brown girls...as though they are older than what they are and so you’re taking away that innocence from them in a sense of expecting that they are.” (Walker 2022)

Similarly, individuals with certain identities may face barriers on the basis of providers’ perceptions of particular identities. This includes responses within social services finding service providers who are more likely to assume that Black women have their own resources for leaving an abusive partner, stymied in inaccurate stereotypes of the “strong Black woman.” (Gerassi 2018)

Due to biases toward individuals who are sex-trafficked as well as against individuals from BIPOC groups, patients might be viewed by providers as “bad people” who have chosen to engage in “deviant” behavior. (Ortega 2022)

Providers are often trained to notice certain criteria in order to identify trafficking victims, including tattoos, carrying condoms, appearing “fearful” or “paranoid,” deferring to another person before speaking, dressing “sexy,” and/or missing documentation. While these things may be common among people experiencing certain kinds of trafficking, they also can be indistinguishable from other life experiences or demographics. We may see similar behaviors among people with limited English proficiency, housing-insecure people, people living in poverty, those with mental health conditions, sex workers, queer and transgender people, neurodivergent individuals, and more. Relying solely or uncritically on vague identifiers has unintended consequences, such as making false determinations of trafficking based on a biased profile… or discouraging people from seeking out services... Many of these “red flags” are also based on racist stereotypes and colorism. These kinds of “red flags” have been harmful to those in interracial relationships or in mixed-race or adoptive children, such as when people falsely accuse someone’s significant other or friend or parent or relative or guardian of trafficking based solely on racist stereotypes. (Larson 2023)

Stereotypes about black women and girls being immoral, sexually deviant, and hypersexual intensified perceptions that black girls who engaged in commercial sex were willful criminals rather than vulnerable victims. (Rothman 2018)

Another aspect of adultification is the widely held assumption that Black girls are ready for and engaged in sexual activity in early adolescence, which can often lead to the perception that Black girls simply choose to engage in sex work instead of recognizing their marginalization and vulnerability to exploitation... On the other hand, if they are recognized as victims, then they are blamed for their victimization (ie, the adultification of Black girls). This flawed knowledge, if left unchallenged, influences how services are provided… (Cook 2022)

"black individuals who have been exploited are less likely than their white counterparts to receive helping services when they come to the attention of social service providers or law enforcement as CSEC victims, and more likely to be perceived and treated as delinquent juveniles." (Rothman 2018)

Subcategory: Invisibility

Most of the white providers did not offer descriptions of racial demographics among clients and, when prompted further, typically pointed toward demographic trends of economic standing, poor family relationships, and/or runaway or homeless youth status. Only when prompted to specifically discuss the racial demographics of their caseload that they, in some cases, offered observations about the race trends of their caseloads... Indeed, providers' attempts to avoid stereotyping, though possibly well-intentioned, only further erased race in a color-evasive narrative, which has been shown to potentially cause harm to people of color. (Gerassi 2018)

...individuals of color report feeling invisible and isolated when accessing services, because they often experience barriers that remain reinforced through judgment, discrimination, and stigmatization. (Gerassi 2018)

Black girls are not recognized as potential victims of DMST, and their victimization often goes unnoticed by those with the power to intervene. (Cook 2022)

Biases, assumptions, and stereotypes about victims from marginalized communities and immigrant populations may hinder identification. (Salami' 2021)

"since they are hyper-sexualized compared to their white peers, they are often too easily dismissed for their perceived actions or attitude, "'cause a lot of times, especially black and brown girls, manifestations of anxiety and depression can come off as anger issues or attitude issues... And that could just be dismissed." She shared that some of her teenage patients who went to the emergency room (ER) with anxiety attacks "were kind of just dismissed, like, 'Oh, you're fine.' And then I see them in the office, and I realize that they're not fine. And really, they didn't have that safe space to be heard in an ER setting." (Walker 2022)

Subcategory: Unequal care

"In general, BIPOC receive lower-quality healthcare and are more likely to be diagnosed with severe mental health disorders. Unconscious racism and a lack of cultural awareness among service providers can lead to discrimination, the provision of lower-quality services, and early termination of services for BIPOC." (Ortega 2022)

"as our participants noted in discussions, emergency rooms are often the first or only point of contact that trafficked persons might have to seek support. Yet, sometimes there is overt racism and stigmatization of commercial sex in the delivery—or denial—of health care services." (Nagy 2020)

"A common theme found throughout survivors' interviews was that of unequal treatment during their healthcare visits... Racial bias within the healthcare system impacts the care a patient receives, potentially leading to poorer patient outcomes and influencing the way individuals perceive and engage with healthcare in the future... Many survivors of human trafficking considered the care they received to have been affected by biased perceptions on the part of HCPs." (Murdock 2022)

"Among the most important rights of any patient is to receive optimal health-care that is free of bias and discrimination. This is of particular importance when working with exploited children, who often face stigma and bias at many levels. Trafficking and exploitation disproportionately affect persons of color, those identifying as LGBTQ, and children living on the

street. Cultural biases against these groups, as well as against non–US citizens and those living in poverty, can have a profound negative effect on the quality of services available to exploited children." (Greenbaum 2021)

“Shula, who works at a support center for transgender women, described the complex situation faced by Israeli Palestinian transgender CSEY who are both banished from their communities, and suffer institutional discrimination: Most of the young women we see are transgender women from the Arab sector, because for them, coming out [as transgenders] usually involved violence…Sometimes there are also language barriers, or racism that they’ve encountered in the system. So, it’s like, who can say that Shula isn’t guilty of that, too? So in these instances, forming a connection is harder […] and they feel like no one in the world will ever help them—especially in a country of Jews, where they feel unwelcomed as it is." (Prior et al. 2023)

Structural & Sociocultural Barriers to Care

Subcategory: Carceral environments

She also encouraged that we consider how service environments can also create tension for youth when she stated that: With armed security or police there’s an adversarial relationship between many members of the African American community and the police. So what ways might we set up young people to already be tense or uncomfortable or feel criminalized as they’re coming into services for the intervention…Having an affirming or empowering environment as opposed to one where young people are initially criminalized and their behavior is being surveilled… (or) reacted to with force. I think removing that dynamic may also allow young people to feel more comfortable and express themselves. (Bounds 2020)

It is important to recognize that while clinicians may view the therapeutic space as “safe,” the client may perceive that same space as “threatening.” In most treatment settings, imbalanced emphasis on “protection” of victims of trafficking results in involuntary, locked, and judiciary involvement that further interferes with efforts to “prepare” survivors by incorporating community, family, mentorship, and culturally responsive programs. The foundations of the current anti-trafficking movement in a criminal justice response have resulted in what Musto classified as “carceral protectionism.” At times trafficked people are treated as “victim-offenders,” instead of victims. This approach can mimic the pattern of control that occurred between a trafficked person and their trafficker. For example, victims of trafficking sometimes stay in shelters, but the shelters are locked down and have policies that resemble imprisonment. (Ortega 2022)

Subcategory: BIPOC erasure

Participants… reported that they have had limited explicit training… related to contextually or culturally relevant services for low-income African American girls in urban settings. According to these service providers, their graduate training was limited to general cultural diversity classes or that they received their training for working with low-income African American girls in urban settings through field experiences... (Harper 2013)

Most providers (n = 20) in our sample received trainings that, to their recollection, did not focus people of color’s disproportionate risk or vulnerability to sex trafficking. For example, Eliza, a white therapist, took two human trafficking courses at different points of her college and master’s education. When asked about whether the trainings she attended addressed the disproportionate risk of particular communities, such as people of color, in sex trafficking, she replied “Not a whole lot that I can remember.” When trainings discussed disproportionate impact, there was less of a discussion of structural racism and its impact on people of color, but rather a focus on their circumstances, such as runaway and homeless youths. (Gerassi 2018)

This widespread omission of labor trafficking in antitrafficking efforts reinforces structural inequities that we have seen through the United States’ history in responding to trafficking. The devaluing of people experiencing labor trafficking, who are likely to be people of color, including immigrants of color, has roots in racism and xenophobia. ...As emergency medicine scientists explore how we can best respond to trafficking, we must not perpetuate existing biases present in the larger antitrafficking response. (Burns 2021)

All the WOCAs stated that the fact that Black and Brown girls seem to be disproportionately targeted as the reason that people don't care as much about the issue and specifically why especially the racial and gender disparities of DMST are not forefronted as much... (Walker 2022)

Subcategory: Cultural barriers

Aspects of health systems and healthcare provision that impede access for survivors of specific cultural groups and create a lack of culturally responsive healthcare services for BIPOC patients who have experienced trafficking.

Interviewees also commonly discussed that Western medical models, even if they are trauma-informed, do not provide the ongoing healing and support trafficked women need to escape prostitution and sex trafficking (MMIW)

These differences in treatment of BIPOC people impacted by sex trafficking and commercial sexual exploitation in the USA are due in part to historical and contemporary traumas of racism and racialized practices, policies, and ideologies. Treatment settings are often ill-equipped to manage… patients whose values and culture deviate from dominant cultural groups. (Ortega 2022)

…survivors also had difficulty disclosing their trafficking and current illicit massage business experiences to service providers. A provider talked about how there was no term to even describe "sex trafficking" in the Chinese language. Coupled with internalized stigma, women processed their trafficking experience in various ways, including not identifying as a survivor, reframing trafficking in a different and often more general manner (e.g., "something bad happened to me" as a euphemism for "rape" (ID009, Mental health counselor), and frequently shaming themselves for being exploited (e.g., "I brought myself into this") (ID009, Mental health counselor). (Lim 2023)

Institutional factors were focused around the types and quality of support services, including the existence or absence of culturally appropriate and/or trauma-informed services. Some factors that impeded receipt of support services included lack of culturally competent providers who can communicate in the survivor's language; service providers located far from survivors' residence or work requiring lengthy travel; and lack of trauma-informed medical providers. At the time of the study, there seemed to be two trauma-informed medical providers in Manhattan specifically working with sex trafficking survivors. Unfortunately, despite the trauma-informed provision of care, the medical provider did not have capacity to provide care in the survivors' languages. One provider commented: "I think we've had mixed reviews [from survivors], because it's never natural to talk to someone about such personal things with kind of the filter of the translators." (ID 002, Attorney) (Lim 2023)

Subcategory: Fear of law enforcement: arrest/deportation

Fears among BIPOC patients who have experienced trafficking, that engagement with the healthcare system will lead to incarceration, deportation, or involvement with child protective services.

Healthcare and other service providers, in efforts to support people whom they know or suspect are experiencing violence and exploitation, may feel compelled to involve law enforcement without the consent of the potential survivor. In a few states, there may even be mandatory reporting requirements for trafficking or other intersecting forms of violence, even for adults. The forms of immigration relief that are available to undocumented survivors of exploitation and violence often mandate cooperation with law enforcement, even at risk of harm to the survivor or their family. Non-consensual involvement of law enforcement has been associated with harmful safety and health outcomes and erodes trust in healthcare and other services as viable resources for people experiencing human trafficking, particularly those who are undocumented, BIPOC, LGBTQ, living in poverty, or in a criminalized economy. (Larson 2023)

Family regulation systems are overly relied upon by healthcare and social service providers. Organizational policies frequently encourage mandatory reporters to err on the side of making a cautionary report, rather than exploring creative ways to support the person's or family's needs. Poverty is often equated with neglect, which has a disproportionate impact on families of color. Healthcare and education systems have biased and harmful mandatory reporting practices that inflate child welfare cases and removals. These removals have created lasting intergenerational trauma among Indigenous communities. The Sixties Scoop, which started in the 1950s and continued into the early 1990s, for example, saw thousands

of Indigenous children stolen from their families by child welfare departments in Canada and adopted out to white families, including families in the U.S. The impacts are devastating to this day. Removals also disproportionately impact Black mothers and families, as well as migrant families. (Larson 2023)

Ethnic minority populations and undocumented immigrant groups, to which many human trafficking victims belong, may be particularly weary of seeking mental health services due to… fear of stigmatization, deportation, hospitalization, or incarceration… (Salami' 2021)

NHS charging regulations [which require providers to determine if a patient is an "overseas visitor" for medical billing purposes] and information sharing with the Home Office negatively affects access to healthcare for unrecognised victims of modern slavery, particularly those from ethnic minority backgrounds. (BASNET)

Similarly, Nina from the expert panel highlighted that trusted adults may be reticent to participate in the intervention because of concerns about maintaining privacy, "Black people don't want people coming in and getting into their business." (Bounds 2020)

Healthcare Practice Recommendations

Broad Recommendations

Subcategory: Cultural responsiveness

When working with exploited children, the clinician can encounter a variety of cultural differences that impact provider-patient interactions. For example, a patient might assume a major power differential in his or her relationship with the pediatrician, expecting the clinician to dominate the conversation and make all treatment decisions. The interaction might become awkward when the physician takes a more egalitarian approach, using trauma-informed techniques to encourage the patient to ask questions, voice opinions, and participate in decisions. Gender roles and cultural taboos about sex and sexuality can be extremely important, and respecting a patient's desire for an examiner of a particular gender is advisable whenever possible. Cultural views about sex outside of marriage, prostitution, and gender identity can have a profound effect on how a patient views his or her own situation and describes it to the clinician. Patients can have very different views regarding their life situation (fate versus divine punishment), the cause of their health problems, the acceptable ways to manifest illness, and the ways they expect clinicians to treat their health problems. Emotional distress can be manifest and interpreted in ways that are impacted by cultural views regarding mental health. Discussion of emotions might be taboo in certain cultures; some languages might lack words to describe certain emotions. (Greenbaum 2021)

Afrocentrists offer practitioners several salient culturally relevant factors for consideration when developing and implementing treatment interventions in working with African American families… peoples of African descent are not a homogenous monolithic group but may share some commonalities with respect to their overall worldview… common ancestral past and cultural factors and traditions… can help explain some of the ways in which Black families have responded to the traumatic conditions of enslavement, colonization, resettlement, and contemporary challenges… an Afrocentric explanation of social problems may not be applicable in all situations or social conditions because it is not a universal panacea for all issues affecting the lives of African Americans. Afrocentrists emphasize understanding the historical roots of Black helping networks and traditions and the cultural values of Black family life, including the value of oral traditions, eldership, Black spirituality, and collectivism. (Valandra 2018)

Equally important, the unique role of shame and its amplifying effects on stigma should be considered. Traffickers are very savvy and exploit these very deep-rooted cultural norms around shame as a way to exercise control over their victims; even post-trafficking, study results show that shame and internalized stigma continue to hinder survivor recovery in distinctive ways. Rather than taking a universalistic view, interventions that are cognizant of the specific cultural context of shame and stigma may show increased uptake, continuation, acceptability, and efficacy in this population. (Lim 2023)

A clinician's familiarity with cultural and somatic idioms may aid their understanding of multicultural victims' trauma-related symptoms. For example, study results indicated that PTSD criteria was most prevalent among women who narratively described experiences of severe sustos. Susto depicts a state of fear or fright, which could be underrated as a symptom of PTSD if one presumes the term to represent a surface-level concern. Therefore, an evaluator should not undermine the importance of semantics when presented with multicultural victim narratives that include cultural idioms. Ataques de nervios (i.e., attack of nerves) is a cultural syndrome commonly seen among Latin and Hispanic populations. Ataques de nervios may entail aggressive or violent outbursts, resembling malice intent, upon an individual's experience of significant stress reaction. By appreciating culturally influenced beliefs and behaviors, mental health professionals enhance therapeutic approaches suitable for victims in distress. (Davidtz 2022)

Individuals working with Native American populations have been urged to closely examine provider requirements for the health care of Native Americans so as to recognize cultural differences in symptom expression and language, and symptomatic patterns among such populations with mental illness. (Gerassi 2018)

The spiritually conservative values and morality demonstrated in African American communities make it critically necessary that practitioners frame discussions about sexual behavior within the context of promoting child and youth safety and protection to avoid any misinterpretation of "sex talk" as promoting or encouraging sex among minors. (Valandra 2018)

Sue and Sue also described how it would be advantageous for a clinician to remain "in the process of actively developing and practicing appropriate, relevant, and sensitive intervention strategies and skills in working with his or her culturally different client." In developing innovative practices, it is beneficial to explore past and other germane research for inspiration. (Villareal Armas)

Professionals should also be willing to speak directly about cultural issues and ask questions with respect and humility. These efforts will support the building of trust and the survivors' sense that they are being valued, respected, and heard. They can also bring about greater disclosures that are less retraumatizing, and thus facilitate case preparation to address human trafficking as well as recovery and prevention efforts. (Bryant-Davis 2017 #2)

Participants (n = 5) reported that language (level 3) is an important aspect of culture that facilitates establishing rapport with girls or caregivers during direct services. Service providers reported that they sometimes "code switch" or "use the slang" to more closely match girls' communication style or are more cognizant of language used when conversing with caregivers. (Harper 2013)

Further information about cultural beliefs and practices can come from speaking with leaders and others in the community, talking to staff at relevant community service organizations, such as those serving refugees and asylum seekers, or seeking online resources such as A Practical Guide to Implementing the National CLAS Standards (2016). (Greenbaum 2021)

In order to adhere to cultural competence, Sue and Sue advocated for an emic model, which takes into account the diversity of individuals and their cultural backgrounds. As opposed to the etic or culturally universal perspective, the emic perspective is culturally specific. With an emic perspective, it becomes clear that this is not one therapeutic orientation that will work for everyone. Rather, it is important to keep the individuality of each client in mind. (Villareal Armas)

Therapists should also be comfortable with a range of affective responses including but not limited to mourning one's losses, anger/outrage, and somatic complaints which are more often endorsed by culturally marginalized survivors. (Bryant-Davis 2017)

Discussion of certain topics, such as sex or illness, may be taboo with others outside their culture. The HCP must have the humility to accept and acknowledge that there may be much about the victim's culture they do not understand, and that the impact of such taboos may be significant in that culture. (Crane 2011)

Health care providers should remember that every culture has a distinct framework or perspective about mental health and, as a result, distinct beliefs about the benefits of seeking mental health services. Counseling, in general, is a predominantly Western practice; in some cultures folk healing, healing rituals, and secret societies are the commonly accepted forms of health care provision. (Rajan 2021)

Mental health care providers should familiarize themselves with the beliefs, values, and practices of the various cultural contexts of their patients, so that they are able to provide culturally competent care. (Rajan 2021)

Practitioners are well advised to understand the cultural background of the people whom they are most likely to serve and to seek information about the implications of the cultural beliefs on risk for CSEC and interventions for CSEC survivors. (Rothman 2018)

Agencies and organizations working with Latina youth should have a thorough understanding of marianismo and machismo, both the expectations set forth by them as well as the results of adhering to those values." (Rothman 2018)

Be cautious of cultural viewpoints relevant to care (e.g., Vietnamese preference for "healing practices" over "therapy") (Davidtz 2022)

When working with Thai women who have endured sexual slavery, it would be prudent to consider the impact of their sociocultural background on their traumatic experiences and worldviews… It has been well documented that, due to their spiritual beliefs and sociocultural background, these women feel shame and believe that they are reprehensible because of their misfortunes. By having knowledge about these beliefs, clinicians may gain more insight into the mentality of Thai survivors of sex trafficking. (Villareal Armas 2010)

it has been argued that the Latino cultural values of *machismo* (i.e., suggesting men are courageous, honorable, virile providers with authority over women), *marianismo* (i.e., dividion of labor according to traditional gender norms), *mujer decent* (i.e., remaining a virgin until marriage), and *familismo* (i.e., the duty to preserve the honor of the family) are so central that Latino survivors of CSEC may face particularly challenging struggles related to stigma and reintegration into the family. In her 2012 report, Sarah Flinn writes, "It is important to remember that these cultural values do have an effect on Latina youth in situations of sexual exploitation, although that effect caries from case to case. (Rothman 2018)

We encourage clinicians to take advantage of resources available through the APA such as its Guidelines for Providers of Psychological Services to Ethnic, Linguistic, and Culturally Diverse Populations. Through the Council of National Psychological Associations for the Advancement of Ethnic Minority Interests, APA also publishes a handbook entitled The Psychological Treatment of Ethnic Minority Populations. (Salami' 2021)

Culturally competent and linguistically appropriate social and health services… (Lim 2023)

Cultural responsiveness refers to healthcare services that are respectful of, and relevant to, the health beliefs, health practices, culture, and linguistic needs of diverse consumer/patient populations and communities. (Ortega 2022)

Recommendations for working with sensitive topics such as mental and sexual health require a cultural lens that reflects an understanding of the population being served (Bounds 2020)

It [cultural competency] is the way patients and doctors can come together and talk about health concerns without cultural differences hindering the conversation, but enhancing it. (Robinson-Dooley 2013)

Cultural competence is crucial in the treatment of emancipated Thai MDS. In general, failure to practice in a culturally competent manner may lead to secondary & tertiary traumas. According to Sue and Sue, "A culturally competent helping professional is one who is actively in the process of being aware of his or der own assumptions about human behavior, values, biases, preconceived notions, personal limitations, and so forth... One who actively attempts to understand the worldview of his other culturally different client... One who is in the process of actively developing and practicing appropriate, relevant, and sensitive intervention strategies and skills in working with his or her culturally different client." (Villareal Armas)

For a psychologist from the Western world, it may be difficult to understand the self-sacrificing and self-blaming tendencies of Thai MDS. It is important to acknowledge these cultural biases and to progress with an empathetic attitude. It might be iatrogenic and damage rapport if the clinician challenges the survivor's self-blame. (Villareal Armas)

it is important to keep in mind an emic model of cultural competence, which takes into account within-group differences. Through interactions with each female client, the clinician may get a sense that one former sexual slave may not be receptive to these mindfulness practices whereas another survivor would be. (Villareal Armas)

When clinicians practice cultural competence, the worldviews of MDS are taken into consideration, and their voices are heard. (Villareal Armas)

It is also critical that they develop culturally congruent responses to trafficking victims in order to enhance trust and respect in ways that will foster retention and rehabilitation. (Bryant-Davis 2017 #2)

In an effort to assuage some of these fears [stigmatization, deportation, hospitalization, incarceration – re: ethnic minority patients], therapists should adopt a culturally sensitive and trauma-informed approach to clinical care. A culturally sensitive approach takes into consideration the unique and diverse backgrounds of clients. (Salami' 2021)

Diversity is just one challenge a psychologist faces while working with this marginalized population [modern-day slaves], which is composed of individuals from various countries worldwide. Furthermore, due to the complexities of modern-day slavery, cases are highly individualized. Although there are common themes, each individual typically has a unique, intricate history of captivity. When working with MDS, it is important to diagnose and formulate a treatment plan with the individuality of each client in mind. Adhering to cultural competence in trauma therapy is a wise first step. (Villareal Armas)

Subcategory: Understanding context

Further, therapists should aim to be informed about sociopolitical factors that may be particularly relevant for foreign-born victims, such as immigration laws and visa programs, poverty and homelessness, cultural and institutional racism, and issues of safety and security. The sociopolitical climate may impact service utilization if clients come to view treatment centers as a microcosm of the larger society, and fear discrimination or other negative consequences such as deportation when using services. (Salami' 2021)

In order to fully understand how trafficked individuals experience their exploitation, one must understand the structural and cultural frame that trafficking occurs within. The experience of a trafficked individual sits at the nexus of multiple structural factors including socioeconomic status, family structure, race, gender, the criminal justice system, and experience of adverse childhood experiences. These factors contribute to an individual's risk of trafficking, their ability to leave trafficking, and their recovery from the various physical and psychological sequelae of trafficking. (Ortega 2022)

Importance is also placed on the historical oppression and trauma of slavery, the continued economic, social, and political marginalization experienced by Blacks, and the persistence of this marginalization through pervasive racial disparities and poverty. It is critical to understand how ongoing exclusionary and discriminatory policies and practices have affected the well-being of family life across generations and have led to the physical and mental health disparities in African American communities today (Valandra 2018)

Further, the exploration of difficult traumatic memories and ongoing traumatic circumstances, including those related to social oppression (e.g., racism, sexism, homophobia, classism, ableism), should be initiated by the practitioner in the context of a safe and collaborative relationship. (Bryant-Davis 2017)

While Black, Indigenous, and other people of color, colonized or subjugated populations, and women and gender-nonconforming people do experience increased rates of violence, this violence is due to cultural, systemic, and structural norms, policies, and practices… Be aware of how focusing on individual-level "risk factors" can be pathologizing, might feel like prescribing future trauma, or can even lead to "pre-emptive" criminal legal responses... Risk is typically created by systemic failures rather than individual shortcomings. When we think about individual risk factors, we must always connect them back to the larger systems and policies that create those conditions. (Larson 2023)

Recognize that Black girls are not at risk of DMST because of their race, but because of a racialized social order that places Black girls at the margins of society and increased vulnerability to DMST... Incorporate an antiracist lens by acknowledging that Black girls are chronically racialized and experience racism in the context of DMST (eg, history of exoticism of Black female bodies including the oversexualization of Black girls in contemporary media) (Cook 2022)

Human trafficking is widely complicated by the displacement and vulnerability caused by regional wars, conflicts, and natural disasters… Working with human trafficking survivors who are Asian immigrants, or immigrants from other regions necessitates attention to the specific country of origin, a complete trauma history, exposure to cultural oppression, and utilization of cultural resources. (Rajan 2021)

The authors of the study stress that the women's strengths as well as their vulnerabilities must be seen in the context of a history of colonial harm on Native people, racism, poverty, and a lack of housing, lack of equitable health care, and lack of job/educational opportunities. (Robinson-Dooley 2013)

Critically conscious and anti-oppressive approaches:... a few providers (n = 5) acknowledged the intersections between racism and classism, in accordance with a critically conscious approach, and the description of the overrepresentation of people of color among their clients. Jessica, a white counselor, suggested that "so many times women of color in our area are at poverty. Anybody that's at poverty can be at a higher risk."... she went on to describe this heightened vulnerability, [Women of color] are much more likely to be vulnerable because you're a minority. There's a lot of biases, maybe, that people have. Definitely because when we're looking at the women of color in our area, many times they are earning the same amount. So now you've got a mom that has three or four children, and you're going," I know that she's a prostitute." I'm quoting, "And why is she doing that?" So we're going, "Whoa, whoa, whoa, whoa, whoa, she is not a prostitute. She is being prostituted. Somebody's exploiting her because she doesn't have the income to take care of her children." As described here, Jessica viewed the impact of structural oppression influencing the overrepresentation of women of color at risk of sex trafficking. (Gerassi 2018)

The ideal clinical encounter is one in which both the provider and the patient are keenly aware of the impact of racism and bias on mental health, and they are both able to freely discuss the experience and consequences of racism. (Ortega 2022)

It is important to acknowledge the role of race when working with this population...too often we do not engage in conversations with youth regarding the implications that race has had in their risk for trafficking, their exploitation, and the services they have received. (Williamson 2020)

Every person's subjective experience of violence and exploitation is complex, and we need to consider the person in the context in which they have been living... this necessitates decolonial trauma and violence-informed supports, that are culturally relevant and sensitive to harm reduction approaches. Together, these approaches understand trauma not as an isolated event, but as embedded in colonial violence and other forms of marginalization. (Nagy 2020)

Human trafficking is a complex subject that is impacted by a variety of cultural and structural factors. Clinicians who are aware of these complexities can greatly improve the care of sex and labor trafficked patients, particularly those of BIPOC groups. (Ortega 2022)

Attending to sociocultural context and systemic barriers are key aspects of womanist therapy. These factors and the literature on vulnerabilities to sex trafficking point to the need for womanist therapist to attend to prevention and intervention around child abuse, poverty, gang involvement, need for love and acceptance, curiosity about sex work, desire for fast money, substance use, gaps in foster care, attention to families in crisis, youth with academic difficulties, and capacity to distinguish boyfriends from traffickers. (Bryant-Davis 2019)

Culturally contextualized treatment of African American sex trafficking victims requires a recognition of the potential impact of oppression, namely intergenerational trauma, historical trauma, race-based traumatic stress, and posttraumatic slave syndrome. (Bryant-Davis 2019)

A womanist, or Black feminist, framework for addressing the sex trafficking of African American girls and women requires attending to the sociocultural factors and realities of intersectional oppression that increases the risk for trafficking, as well as the cultural strengths that can be utilized to address prevention and intervention strategies. (Bryant-Davis 2019)

South Asian trafficking survivors face the realities of poverty, risk of HIV, lack of social support, untrustworthy migration facilitators who are actually traffickers, violence, cultural and religious beliefs that support the exploitation, and sex tourism that consistently seeks out children at younger ages. One survivor describes her experience in these words: "My stepmother never wanted me to go to school. I was a good student and yet I never got a chance to go to school after she married my father. I had two younger sisters and my new mother gave birth to a son that year. She told me that I was the eldest and since my father had lost crops twice in the season, he would like me to take up some job. I knew how to read and write, so I at that time she was asking for my support. I also heard my father talk to the landlord about getting me work in a nearby city. But, I had no idea that they were selling me for money. When I went to Mumbai with six other girls, we were asked to do all kinds of jobs for no money. Money was directly sent to my family." This survivor's narrative supports the need for an intersectional feminist frame that attends to her multiple oppressed identities, gender roles, family roles, duty/obligation, exploitation, and secrecy. While these oppressive realities must be acknowledged, a practitioner working with this survivor would also need to acknowledge and integrate her individual and cultural strengths. (Rajan 2021)

The experience of a trafficked individual sits at the nexus of multiple structural factors, all of which contribute not only to an individual's risk of being trafficked but also to the ability of that individual to leave trafficking, and the way in which they recover from the various physical and psychological sequelae of trafficking. In order to have some understanding of how trafficked individuals experience their exploitation, one must understand the structural and cultural frame that trafficking occurs within. Health professionals who embrace an intersectionality lens commit to understanding the systems, structures, policies, and practices that put individuals based on their intersecting identities, at increased risk for discrimination, prejudices, and oppression. These factors will impact the way that a trafficked patient presents clinically and will also affect how we manage their psychiatric needs. Figure 3.1 depicts several structural factors that intersect to affect human trafficking survivors in the USA: the criminal justice system, socioeconomic status, family structure, race, gender, and experience of adverse childhood experiences. (Ortega 2022)

These guidelines highlight the importance of attending to clients' context, including their cultural background, socioeconomic status, social support network, and the realities of oppression, stigma, and discrimination. (Bryant-Davis 2017 #2)

The consideration of structural oppression should be part of every component of assessment and intervention, ranging from the establishment of safety in one's immediate circumstances, the processing of traumatic clinical material, to establishing new and safer connections. (Bryant-Davis 2017)

With respect to legal aid, therapists should become more educated about their clients' legal rights by working with local legal resources. To support this initiative, the Office of the High Commissioner for Human Rights (OHCHR) provides a useful fact sheet explaining how human trafficking is a human rights violation. (Salami' 2021)

Understanding the cultural and structural factors that have positioned a youth to be trafficked may help providers have a more complete and trauma-informed understanding of why youth are trafficked. (Ortega 2022)

Subcategory: Relationship-building

The need to get to know youth and their personal experiences was coupled with the need to ensure safety. This theme was endorsed by both youth and content experts. I think that a lot of people (need to) tell their stories first…You all know we homeless so that's why we're here, but you don't know our stories…like let me tell you a story first. Then, after they tell their story, then you can say, all right, so this is how STRIVE can benefit you. (Bounds 2020)

The same participant talked about the strong bonds that she developed with providers at the anti-trafficking organization: "I have been coming here for so long and I feel supported and welcomed by [the service providers]. I feel like someone is there for me like family. I feel that way here." (ID 003, Survivor, Korean Chinese) The strength of those bonds may be the result of providers' trauma-informed approach. A mental health counselor shared how she is careful not to push women to share their stories and how she places importance on person-centered care and respecting women's autonomy: "Well, I think it was that many of them denied engaging in sexual behaviors still, still to the very end, "I didn't do any of that stuff

[forced sexual activity].” I think it is always tricky for a counselor because it’s our job to believe them. … I think especially with clients who have experienced sexual trauma, and trafficking, even admitting those things… is very challenging. Even saying, things like being raped or addressing even like what it is that happened is also very hard… it’s important to be aware of because clients do experience a lot of these things without verbalizing it." (ID 006, Mental health counselor) (Lim 2023)

Just as survivors of other forms of trauma should be met with support and belief, persons who disclose experiences of discrimination, bias, oppression, and racism should be met with support and belief. Attempts to justify, explain, or deny the experiences of marginalized clients are sources of retraumatization. (Bryant-Davis 2017)

Healthcare and social service providers should listen to the concerns and needs of patients and clients. This may sound simple but is often overlooked because the provider has a mental script that makes them think they already know what the patient/client needs. Many competent providers who respect people-centered care may shift into rescue narratives when they suspect human trafficking, forgetting that the values of nonjudgmental, people-centered care still apply... Do not assume you know what anyone’s concerns or needs are before they tell you. This includes being careful to avoid medical and institutional fatphobia that has historically targeted and alienated Black and Indigenous survivors, in favor of weight-neutral approaches that focus on overall health. It also includes cultural humility and recognition of non-Western and Indigenous approaches to holistic healthcare, including for mental health. (Larson 2023)

Being relatable was emphasized by both experts and youth across settings. One youth articulated: You gotta relate to what people are goin’ through. You gotta be able to, I guess, like if you’re not from the hood you gotta be able to put on the shoes of somebody that is. You gotta try to see it from somebody else point of view regardless of what you’re comin’ from. (Bounds 2020)

Shared ethnicity and/or gender. Participants (N = 7) reported shared ethnicity and/or gender (level 2), that is, being an African American or a woman as a main facilitator of relationships with girls or their caregivers… “I’m aware of what happens in the black community…either through my own experiences or relatives’ experiences or just talking with other people about what’s wrong in the world.” … “I just think there’s a sense of comfort…even if it was something as simple as the girls wanted to have a two-minute conversation about…hair. “How did you do your little girl’s hair today?”…They can relate to that…a level of comfort that’s difficult to explain. (Harper 2013)

Lina who works at a shelter for at-risk Arab girls suggested that as an Arab woman, she understands the specific challenges that her clients face: ‘They find it easier to talk to me, because we come from the same culture. If you want to express yourself, it’s easier to do so in your first language. There are nuances and words that you can’t…express, even I find it difficult to express some things in Hebrew. There’s also the cultural context that I can relate to…’ (Prior et al. 2023)

Although participants perceived ethnic/gender similarities as facilitating relationships with girls, they indicated that ethnic or gender similarities are not necessary or sufficient for building strong relationships with girls… “Some people can relate better to the girls than others…if you’re real, they know it…I don’t care what your skin color is. If you’re coming off as a fake black person, they gonna call you fake. If you coming off as a fake white girl trying to be nice to black people, they gonna call you fake. Fake is fake…they call it out.”... “There are non- black people who have a desire to work with our populations, some more so than even our own counterparts… I think that if people’s motives are really genuine and they have an interest in learning about the population and how they can best help them and not just try to change a person because they think the person needs to be changed.” (Harper 2013)

In terms of approaches, we argue for relational approaches that are tailored to the specific needs of trafficked persons, their families, and community. By relational, we mean service provider approaches that honour self-determination and agency, are based in respect and non-judgement, and encourage service providers to act as allies in achieving change, rather than imposing “expert” solutions… (Nagy 2020)

Sahl and Knoepke note that youth who have been victimized by CSE often encounter multiple systems (i.e. juvenile justice, child welfare, community mental health). These systems tend to be prescriptive in their approach and fail to consider the youth’s voice. When youth voices are not considered in systems of care, there is a greater likelihood that youth will not adhere to treatment, run away and be mistrustful of providers. (Bounds 2020)

In particular, we must connect with youth in ways that resonate with the contexts of their lives (Bounds 2020)

Setting the stage refers to the initial phase of the relationship where building rapport and acknowledging experiences of structural violence are essential. (Bounds 2020)

There was also a theme amongst the girls about needing their educators (teachers, counselors, and administration) to care more about them on a personal level than just academics–through both individual interactions and structural support. On an individual level, Aziza shared that most young people, at least girls, just want educators "to know who they are and what they're dealing with without preconceived notions, and how you can provide the best education for them emotionally and academically." (Walker 2022)

It is also critical that clinicians build a strong therapeutic alliance and create a sense of safety for clients in order to promote recovery, rehabilitation, and, ultimately, reintegration. (Bryant-Davis 2017 #2)

Taking the time to build rapport is critical. For many, cultural, power, class, and racial differences exist prior to the encounter. Self-protective mechanisms lead to distrust of authority figures and in turn to defensive reactions… Many small steps are needed to build trust, such as open-ended questions, few interruptions, and a private area to talk. Often more than one visit is needed, and the victim may need to be told to return to the clinic to reevaluate a health care issue when the HCP strongly suspects trafficking and further assessment and questioning is desired to get a patient to open up. (Crane 2011)

Subcategory: Acceptance

In addition to being prepared to meet their needs, expert panelist, Lisa poignantly reminded us of the need for a non-judgmental approach by stating, If we are not going to give them money then we need to be really careful about how we criticize the way they are getting money because it doesn't mean they're gonna stop getting money that way, it just means that they're going to stop talking to us about it. (Bounds 2020)

Positive regard. Participants (N = 7) reported that using positive regard… is important for working with girls this population. The positive regard code included strategies such as treating girls with respect; avoiding confrontation, derision, or sarcasm; and active listening to girls' points of view when they display emotional or behavioral problems. For example, one participant stated, "That in-your-face kinda tactic doesn't work a lot of times for low-income girls who have a lot of issues." Another participant stated: They're harder to reason with sometimes than boys… People who are more consistent with them and who show respect…they are more likely to accept advice from those people, even when they are being very stern with them. They need to have their perspective heard… they have to know that you at least respect that they have a point of view before they can hear an alternative. One participant reported that when girls are combative, that she uses a non-combative response as an opportunity to model effective communication skills. (Harper 2013)

Experts recommend using a "harm reduction" approach to working with Indigenous sex trafficking victims and survivors, both with regard to their involvement in sex work and addiction. This means "accepting sexually exploited youth and adults wherever they are in life and trying to improve their safety in non-coercive ways." (MMIW)

Finally, two mental health counselors working with Chinese survivors shared the many ways in which they use a harm reduction approach in their service provision because they were starkly aware that at least some of the Chinese survivors continued to be involved in commercial sex by providing sexual services at illicit massage businesses. (Lim 2023)

Employ non-judgmental, trauma and violence-informed approaches, and harm reduction…

This approach [harm reduction for indigenous sex trafficking victims] encourages service providers to build relationships and trust with the victims… accept and expect the process of recovery is more often a "one step forward, two steps back" path versus a linear path forward; and do all of this with a culture of grace, understanding, and respect for the person. (MMIW) [also Multidisciplinary care & collaboration]

Clinicians who work with trafficked individuals should work to have a nonjudgmental stance. (Ortega 2022)

All systems: Remove shame around discussing healthy relationships, consent, abuse, and sex trafficking (Walker 2022)

With respect to addressing linguistic and communication barriers, it is important that practitioners utilize language that underscores the reality of exploitation, as negative messages blaming the survivor for her "role" in the abuse may have become internalized. (Bryant-Davis 2017)

Subcategory: Power/Agency

The survivor must be an equal partner in treatment plan development. (Robinson-Dooley 2013)

…it must be clear that the clinician is merely offering a suggestion, and, ultimately, it is the survivor's choice to participate. (Villareal Armas)

To allow for greater trust and understanding between therapist and client, clinicians should address hierarchical structures that manifest in the therapy room as a result of historical inequalities between groups, and build a collaborative therapeutic alliance. (Salami' 2021)

A second emergent consideration during the setting the stage phase was the high prevalence of misconceptions and alternative perspectives surrounding sexual exploitation. This is aligned with existing data that suggests that many victims of sexual exploitation do not identify as victims, nor do they label their relationships as exploitative. Content experts in the current study emphasized these misconceptions. Veronica noted the following: "you don't want to marginalize them or turn them off to the study by initially declaring them exploited cause that's not how they see themselves necessarily. Some will– some won't." Amy, a content expert colleague concurred by voicing the following perspective, "Some people will never identify as experiencing exploitation. And so changing that language would be helpful to get people engaged. (Bounds 2020)

It is essential that clinicians and justice officials know there is more to culture than the experience of cultural oppression—that they recognize the cultural strengths of their clients, not just their pathologies or deficits. (Bryant-Davis 2017 #2)

Most importantly, any type of programming should consider harnessing women's sense of resilience and existing skillsets. (Lim 2023)

A feminist, multicultural approach should also involve a formulation of goals and planning of treatment and other interventions that promote racial minority women's empowerment in a society that may continue to oppress women and racial and sexual minorities both socially and economically. (Bryant-Davis 2017)

Being aware of individual self-identification is also an important part of working toward cultural competence. Though it may be obvious to us as service providers that trafficked women are "victims," rarely does a WOC self-identify as such. Working from a culturally competent strength perspective, it is empowering to ask women how they identify and then use that term/label in all future communications. (Robinson-Dooley 2013)

Womanist therapists centralize and celebrate the experiences and perspectives of African American women; they internalize and demonstrate respect for the dignity and empowerment of African American girls and women, which transcends well beyond tolerance, tokenism, pity, idealization, or dehumanization. (Bryant-Davis 2019)

Ask if she would prefer to speak with a Spanish-speaking woman or another woman of color. Do not assume you are the best person for her to speak with. (Robinson-Dooley 2013)

Cultural humility also includes… addressing the power imbalances between clinician and client. (Ortega 2022)

It [cultural humility] also entails a clinician's validation of an individual's own understanding of themselves… (Davidtz 2022)

Power and control issues must be "checked at the door" and a commitment to leveling the playing field must be agreed upon by the team. (Robinson-Dooley 2013)

Clinicians should provide care grounded in culturally responsive and trauma-informed approaches. Steps to providing this care include.... reducing the power imbalances in the therapeutic encounter, talking openly with patients about race and experiences of racism… (Ortega 2022)

Explain what is being done/asked and the reasons. Do not assume that your client understands tests and requests that may seem standard to you. (Robinson-Dooley 2013)

Assessment

Subcategory: Holistic assessment

The bio-psychosocial assessment is an intensive interview that gathers information about the client's medical needs (bio), mental health concerns (psycho), and social networks and social supports (social). Recently, social workers have advocated for collecting data on clients' faith or spiritual beliefs. An understanding of these areas are important to understanding your client as a holistic being. (Robinson-Dooley 2013)

To this end [helping clients form social connections] clinicians will first need to understand the social support needs of their clients and understand how these needs may materialize in a new cultural context. For example, clinicians may glean information about culturally salient community organizations, such as faith-based, LGBT, and ethnic group organizations from which their clients can derive benefit by first asking clients about their values and beliefs. Such organizations may provide a natural setting for clients to meet those with similar backgrounds and gain support. (Salami' 2021)

Mental health providers should not only assess for traditionally recognized sex trafficking dynamics and consequences, but also for cultural meaning making and stigma, spirituality, social support, somatic and medical complaints, and cultural strengths. (Bryant-Davis 2019)

The genogram is a family diagram developed from information collected from the client about their family history… Building a useful genogram requires the collection of a detailed history and nature of relationships from the individual. The client is asked to share the names and background of family members, the nature of the relationship to that family member (divorce, marriage, etc.), and the emotional context of the relationship (tense, close relationship, abusive, stressful, etc.)... Patterns emerge about the function of family for the client… The detailing of the familial relationships in their lives provides a "picture" of how the client interacts with multiple systems and how the client functions within the family environment. This information and diagram will often provide a display of family patterns that assist in explaining family functioning. This information is important to begin any treatment planning for a WOC and trafficking victims. (Robinson-Dooley 2013)

The Eco-map… is focused on the client's perception of the important people, agencies, and social networks in his/her life. This information is collected in the assessment process with the client. People and social networks are displayed in circles that are filled in by the client. The strength of these relationships is displayed with lines between the circles (strong, detached, fragmented, etc.)... Eco-map development can assist with determining the social interactions and social functioning of the client. "Ecomaps provide an aerial view of the external influences at play on people involved in a genogram, hence they are a tool useful for depicting relationships of families and groups." (Robinson-Dooley 2013)

Subcategory: Cultural responsiveness

Another consideration is that most Southeast Asian individuals present their emotional distress as somatic complaints. However, somatic symptoms may be indicative of exposure to trauma. It would be wise for clinicians to ask questions about the client's own insights about the presenting problems, past interventions, and expectations for treatment. (Villareal Armas)

The presence of cultural mediators allows patients to speak in their mother tongue, and different interpretations of the patient's suffering can thus circulate during the consultation. Mediators are asked to explain and clarify symptoms according to the cultural idioms of the country of origin of the patient and can also participate in the diagnosis and cure administered to the patient. Their role is to mediate between the cultural content of the patient's idioms and the explanatory models of the doctors. This allows for a different telling and a different listening... Allowing for the migrants' words to circulate in the clinical setting to talk about symptoms, to name suffering, is a way of creating a space of mediation where the unsaid of the migrant's story can emerge and find its own articulation. (Giordano 2008)

We recognize that there are many cultures in which the concept of the self does not include individuality and boundedness. Thus, assessing an individual's level of acculturation is strongly recommended. When assessing a client's level of acculturation, we recommend the use of acculturation measures that have been developed for the client's racial or ethnic group. These include but are not limited to: the Multidimensional Measure of Cultural Identity for Latinos, the Acculturation Rating Scale for Mexican Americans-II, the Native American Acculturation Scale, the Asian American Multidimensional Acculturation Scale, and the African Self-Consciousness Scale. (Salami' 2021)

Subcategory: Safety

Hospitals – sometimes people get hurt and go to the hospital and they don't recognize the bigger context of what's happening. They are fixing the broken arm but not asking how the break happened. They should do safety assessments. Are you safe at home? My experience is that when that's done they do a terrible job. They are checking a box, not actually setting it up so anyone can answer in any authentic way. They are leaving the husband in the room, or they don't ask like they're interested. (Attorney, key informant) (MMIW)

A victim-centered approach should include an assessment of risk of ongoing threats of violence and exploitation over the course of multiple sessions. (Bryant-Davis 2017)

Create a safe environment so that the woman can share her history and her story (allowed her to cover her exposed breast, not assuming it was okay because she was "a prostitute")... Assess safety immediately... Appropriately attending to her obvious/presenting injuries. (This could have provided an opportunity to build rapport/trust.) (Robinson-Dooley 2013)

Treatment

Subcategory: Expanding strategies

…barriers to care and cultural stigmas associated with mental health services highlight the need for street-based interventions, particularly for survivors who are struggling with substance abuse. (Bryant-Davis 2019)

I do know that a lot of girls, and young ladies from a low socioeconomic area are not as, well, talkative and so talk therapy may not be the best for them. Teaching and giving them planning ideas…I guess you could say therapy or other modes of interventions. Social stories, using imagery, teachable moments, something visual, something that would be a little bit more meaningful for the student, to engage the student in the process. (Harper 2013)

Mindfulness-based stress reduction (MBSR) has yielded promising outcomes among low-income African American women with histories of IPV and PTSD. MBSR addresses trauma-related symptoms by cultivating mindfulness skills and enhancing awareness of internal experiences (e.g., physiology, cognitions, emotions) among trauma survivors. MBSR can be delivered in a group format, in a community setting, or by a layperson without formal mental health training, thus potentially reducing stigma barriers to care for low-income interpersonal trauma survivors. Specific outcomes that have been observed in predominantly African American women samples who received MBSR include significant reductions in PTSD symptoms, increased self-awareness, self-acceptance, self-empowerment, nonreactivity, self-care, compassion, belonging, and decreased arousal and distress. (Bryant-Davis 2019)

Due to limited time and resources, providers will need to take advantage of natural opportunities to enhance girls' mental health and resiliency. Some natural opportunities lie in informal direct services; that is, using every encounter with girls, caregivers or other school personnel to address girls' mental health needs. For example, one of the providers in this study reported that when girls are combative, she responds with a non-combative response as a tool for social-emotional learning. Other natural resources can be promoted by including information about the provision of context- or culture-specific services for low-income urban African American girls in professional training for school-based mental health services providers. Once trained, school-based mental health providers could train or consult with teachers or other stakeholders to develop and implement contextually relevant mental health strategies and interventions to use on a daily basis. (Harper 2013)

Womanist, or Black feminist, treatment models are open to the use of the expressive arts as a culturally congruent approach to healing and empowerment. While there are not published peer-reviewed articles focused on the use of expressive arts with African American sex trafficking survivors, there are a number of studies with domestic and international interventions that have found the use of music therapy and dance therapy as effective tools for working with sex trafficking survivors. Although Schrader and Wendland... is primarily an examination of the expressive arts therapy process, they note that the participants who were Cambodian were more engaged and able to explore trauma material and access their affective responses with the expressive art than with the traditional talk interventions. Children who had been sexually exploited were given music therapy, along with traditional group therapy and individual therapy. Music therapy consisted of singing individually and in unison, as well as music listening, lyric analysis, and song writing. The purpose of these activities was to decrease isolation, decrease stress in the body, increase affect expression, increase comfort with asserting one's self and using one's voice, improve healthy immune functioning through the positive physiological changes, confront difficult topics in a less threatening way, develop positive coping, elicit difficult memories, shift focus to the future, gain self-awareness and self-worth, and increase relaxation. Some of the activities included playing instruments, singing songs about their rights, and creating songs about their good qualities. These songs were recorded and then played back for the girls to hear. These are activities that would be culturally congruent in womanist, Black feminist, oriented therapy with African American girls and women who have traditionally used music for self-expression, coping, and connection with others. (Bryant-Davis 2019)

Because sexual exploitation directly occurs to the body, the body is an integral part of the healing work that is needed. Twenty of the 23 girls at one trafficking recovery center named dance as their favorite activity of the center; scarf dancing, repetitive movement, and circle dancing was done to facilitate self-expression, confidence, comfort with the body, and social connection with the other girls. Various dance forms have emerged from and been made popular by African American girls and women. Building on this cultural resource within womanist, Black feminist, therapy can be an asset to the trauma-focused, client-centered therapeutic process that honors both the individual and their relationship with others. While Schrader and Wendland (2012) incorporated the teaching of traditional Vietnamese dance to the girls in their study, womanist therapist could incorporate teaching African dance, jazz dance, tap dance, hip hop dance, liturgical dance, or line dancing to African American girls and women. (Bryant-Davis 2019)

There is a growing interest in interventions that employ non-Western approaches such as narrative therapy as well as non-verbal approaches such as experiential body-oriented interventions (e.g., group-based art and theater-based interventions) to address complex trauma. Group-based or peer-led approaches have been shown to be particularly useful in addressing stigma and should be considered in program development. (Lim 2023)

Informants gave multiple examples of effective Indigenous healing, such as land based practices, traditional ceremonies, medicine bags, and traditional activities for youth... "Fond du Lac organized an MMIW powwow, a day where people are acknowledging it is happening. Years ago, there was no acknowledgement. There are tables with resources. It's packed. They hold it in January. Most powwows are in summer; everyone comes. And the entire community is engaged in cultural practices, which is healing." (Direct service provider, key informant) (MMIW)

Specific alternatives to traditional Western therapies were suggested for working with victims who emigrated from South and Southeast Asia, particularly the use of holistic healing modalities (e.g., spiritual rituals, massage therapy, acupuncture, deep breathing, meditation, herbal gardening, yoga) to assess the mind-body relationship across issues of mental health. (Davidtz 2022)

Culturally informed, adjunctive treatments such as expressive art therapy can support the healing process by helping survivors verbally express their trauma and make meaning of their experiences. For example, expressive writing, narrative journaling, poetry therapy, and art therapy are creative art interventions that aim to help victims transcend traumatization and thrive. Art therapy in particular has been found to promote biological as well as psychological change, to increase self-esteem, and to facilitate meaning making, the integration of right- and left-brain functions, as well as the integration of traumatic memories and experiences. Implementation of expressive art therapies, if culturally syntonic, can provide survivors with a cathartic experience. (Bryant-Davis 2017 #2)

Due to their focus on compassion and renewal, rituals like Touching the Earth have great potential to address psychological distress specific to Thai women, such as shame and guilt. (Villareal Armas)

Mental health [treatment recommendation:] School-based trauma-informed yoga therapy or dance-based program for girls who've experienced sexual trauma (Walker 2022)

A recent systematic review of the literature concluded that mindfulness-based approaches, such as MSBR, to treat PTSD are promising (Bryant-Davis 2019)

Qualitative studies of Expressive Arts have highlighted their therapeutic effects within marginalized communities and with trauma survivors. Artistic pathways can aid survivors in speaking about traumatic experiences that have felt unspeakable. Folk songs, dance, and art have been used to assist with affect recognition, affective expression and regulation, communication, and coping. (Bryant-Davis 2017)

When practicing mindful breathing, one is fully conscious of one's inhalation and exhalation. Observing the breath, one returns to the present moment, which can be a helpful practice for MDS who are experiencing dissociative symptoms. Rather than merely having the intention of living in the now, the breath gives one a concrete focus, which is in the present… Because slow breathing has calming physiological effects on the body, this practice is an ideal intervention for emotionally distressed and traumatized individuals. (Villareal Armas)

Thich Nhat Hanh has also recommended the use of *gathas*, or mindfulness verses. For instance, during mindful breathing, one could mentally affirm, "Breathing in, I know that I am breathing in. Breathing out, I know that I am breathing out."... Regardless of their locations, clients would be able to return to mindful breathing and the mental recitation of *gathas*. In addition to reciting *gathas* during mindful breathing, movement can be coordinated with the gathas during walking meditation... By focusing on tactile sensations in the present moment, this practice may be ideal for moments of dissociation. (Villareal Armas)

Beginning Anew describes a metaphorically cleansing practice during which one bathes in the water of compassion. The Beginning Anew practitioner expresses regret for past acts and the intention of behaving subsequently in a different manner. The resolution for change is in itself healing. Thich Nhat Hanh states, "It means expressing our regret for mistakes we have made... coupled with a deep and transforming determination to act differently from now on. Because we know that we can act differently, we do not need to feel guilt." (Villareal Armas)

Based on Beginning Anew, Thich Nhat Hanh developed a practice called Touching the Earth. This transformative ritual involves prostrations done in conjunction with guided meditations, which take the format of a dialogue with the Buddha. The practitioner addresses the Buddha through a script, a bell is invited, and then the practitioner touches the ground while prostrating. During the prostrations, the practitioner symbolically offers his or her negativity to the earth. It is believed that, just as the earth absorbs garbage as compost that nourish flowers and plants, the earth absorbs and then transforms the practitioner's suffering and affiliations. Some of the guided mediations describe the compassionate nature of the earth and how the practitioner must emulate its compassionate nature - to have compassion towards one's self and others. The fundamental premise of this ritual is that compassion has transformed power. With compassion, one develops insight on how to gain freedom from suffering. (Villareal Armas)

One variation of Touching the Earth incorporates a guided meditation entitled The Earth as a Solid Place of Refuge. The meditation script encourages the practitioner to perceive the earth as a sanctuary. This perception would be beneficial for former MDS. In general, past research has shown that fear is a predominant feeling for individuals suffering from trauma. A Thai woman can use this meditation to create an atmosphere of safety by taking refuge in the solidity and compassionate

nature of the earth. After experiencing betrayal by loved ones and procurers, she can rely on the stability of the earth and allow the earth to absorb her suffering and to transform it. (Villareal Armas)

Another variation of Touching the Earth includes a guided meditation entitled The River of Life, which involved the visualization of unity with the practitioner's ancestors. During the guided meditation script the practitioner pictures the blood of her ancestors coursing through her own veins. Again, the bell is invited and the practitioner prostrated while offering past negative experiences with these family members to the earth. This is very compatible with Herman's Stage 3, which focuses on reestablishing relationships. (Villareal Armas)

The use of culturally relevant expressive arts, such as dance and music, was proposed as effective in engaging trafficking victims who identified as African American. Other creative strategies, such as sand tray or animal-assisted sessions, were also recommended. (Davidtz 2022)

Safe spaces... included creating spaces where girls have the opportunity to explore their identities, more fully know their worth, truly be truly heard, heal, ask for/receive support, effect change (if they want to), dream and find their passions. Hope, the social worker also talked about “creating that safe space in an unsafe space” for some of her students, which looked like creating safe words or questions in virtual breakout groups for them to let her know they really needed to come to school and see her during the pandemic... Journey, the LPC, stated that “there’s something magical about girls being together in a group and being able to just share their experiences and being able to share just their thoughts around things” in both removing shame and raising awareness to things they have become desensitized to. (Walker 2022)

Subcategory: Holistic support

The experience of being trafficked is mental, physical, spiritual, and emotional; thus, holistic support is necessary. Indigenous participants mentioned the importance of Grandmothers, Aunties, Uncles, and Elders in the community; of knowing the Grandfather teachings; of breaking the cycle of intergenerational trauma; of nurturing the wellness of individual, family, and community, including connections to the natural cycle; and the idea that everyone has a role in community. (Nagy 2020)

Given the ways in which trauma and trafficking in particular can erode trust, reliable social support within one’s community context can be quite therapeutic. Among ethnically diverse trauma survivors, disclosure to one’s informal social networks, when it is met with positive reactions, can result in a decrease in traumatic stress. Culturally congruent, quality, affordable mental healthcare is not always available, particularly in low resourced countries and communities. Compassionate and well-informed emotional and instrumental support from family and friends can be a buffer from some of the negative consequences facing survivors of human trafficking. (Bryant-Davis 2017)

While meaning making can be a challenge in the aftermath of the trauma of human trafficking, the belief that there is a Higher Power who believes them, loves them, and wants good outcomes for them can be a source of strength. Additionally for those who engage in spiritual and religious coping strategies, the use of meditation, prayer, mindfulness, spiritual counseling, engagement with a supportive faith community, use of religious and/or cultural rituals, and reading of spiritual and religious text can be helpful particularly in times of distress. (Bryant-Davis 2017)

Like the creative arts, spirituality provides resources for healing. Helping women develop their own understanding of a higher power may stimulate the creation of meaning, a sense of wholeness, and self-transformation. Acknowledging the importance of spirituality and including spiritual ideals in treatment have proven to be culturally syntonic, particularly for ethnic minority populations. Spiritual as well as religious beliefs are powerful agents of change for various ethnic minority groups, particularly African Americans, as they shape their understanding of justice, salvation, and coping with oppression. Religious beliefs can enhance an individual’s ability to cope with negative life events, and negative life events can lead to enhanced religious faith. For these reasons, the integration of spiritual and religious ideals in treatment with sex trafficked women and/or incarcerated women should be considered. (Bryant-Davis 2017 #2)

Those providing brief interventions should consider maintaining an easily accessible list of multicultural referral contacts (e.g., therapists with bilingual abilities, legal aid, trauma-informed medical providers, sources for welfare services) for when a victim necessitates them. (Davidtz 2022)

Recognizing the higher rates of substance abuse among persons with PTSD and the tendency of traffickers to facilitate substance dependence among their victims, interventions with African American trafficking survivors should attend to both trauma symptoms and substance abuse. An evidence-based intervention that addresses both and has demonstrated effectiveness with African American girls and women is Seeking Safety. Seeking Safety is a manualized intervention based on five central ideas: (a) safety as the priority, (b) integrated treatment of PTSD and Substance Use/Dependence (SUD), (c) a focus on ideals, (d) four content areas: cognitive, behavioral, interpersonal, and case management, and (e) attention to therapist processes. Session content is structured to engage in themes relevant to both PTSD and SUD and to learning a specific CBT skill. (Bryant-Davis 2019)

as well as education and community referrals for lifeskills training and addressing immediate needs (content)...A clinician should be prepared with resources and referrals that not only include ways youth can get their basic needs met but also how to navigate services and systems that help them secure jobs and ultimately survive....To address this need, we plan to create a resource and referral list that would assist in addressing survival needs as they arise. We also see STRIVE as a potential entryway to other mental health services such as family therapy and therefore we will need to be prepared to facilitate ongoing family-focused treatment. (Bounds 2020)

The obvious overlaps in the above approaches identified here include a rejection of Eurocentric knowledges centered on individualism. Instead, a positioning of individuals in their relationships to families and communities allows for envisioning their physical, mental, psychological, spiritual, and social well-being, not in isolation but in an enabling structural environment where coalitions of service providers are well-resourced and equipped with the present toolkit. (Nagy 2020)

Learning about one's heritage including their contributions to society which are often dismissed can be psychologically, socially, and academically advantageous. (Bryant-Davis 2017)

Victims of racism and ethnic bias are in danger of internalizing the negative stereotypes that exist about their communities and themselves. This internalization can result in shame and self-blame which both are related to self-harming behaviors. Positive racial and ethnic socialization on the other hand can create a buffer which allows people to create and maintain a positive view of themselves despite the negative messages with which they are bombarded. (Bryant-Davis 2017)

African American women endorse higher rates of religiosity than both White women and African American men. African American women have reported use of positive religious coping with various traumas… Promising practices with African American female survivors of sex trafficking may attend to the victim's holistic health, including physical, psychological, and spiritual needs. (Bryant-Davis 2019)

Following Renee Linklater, we argue that understanding the concept of resiliency is integral to decolonizing trauma practices. She wrote, "Resiliency focuses on the strengths of Indigenous peoples and their cultures, providing a needed alternative to the focus on pathology, dysfunction and victimization." Decolonizing trauma practices have much in common with Indigenous healing paradigms, such as an emphasis that individual healing is "grounded in social healing," and an emphasis on holistic healing. Moreover, decolonizing approaches not only recognize the harms of settler colonialism but systematically work toward repair and redressing such harms. For example, this could mean working toward the implementation of the recommendations of the Truth and Reconciliation Commission of Canada (2015) and the National Inquiry on MMIWG2S (2019). (Nagy 2020)

The Eco-map provides an opportunity for the client to discuss and describe those multiple external influences that affect their daily lives. The external influences then give the practitioner and the client an opportunity to process the nature of their influence. Most importantly, this tool is done from the client's perspective and often this pictorial view of the external influences can bring some realizations to the client that they were not able to talk about in the past. (Robinson-Dooley 2013)

This approach [harm reduction for indigenous sex trafficking victims] encourages service providers to… make a range of services available to meet victims' needs (housing, food, health care, financial, social interaction, etc.) (MMIW)

In order for any practice techniques to be effective, there must be a multidisciplinary approach to treatment. The foundation of this treatment must be culturally competent and be a combination of efforts by social workers and nurses.

This effort must involve a commitment to cultural competence, a focus that is "woman centered," and use of an arsenal of tools that are effective for building a multidisciplinary team approach to treatment. (Robinson-Dooley 2013)

[In the context of HCPs outside of behavioral health] Finally, it is recommended that a behavioral health referral be a standard of practice, especially for practitioners who might be working in isolation and serving WOC. This referral would involve sending the client to an agency or practitioner for a mental health assessment to determine the client's psychological needs. We must not let the "stigma" of mental health prevent us from providing the assessment and treatment we anticipate women who have been trafficked will need. (Robinson-Dooley 2013)

School counselors: connecting young people to resources at their fingertips: bookmarks with teen dating and domestic violence hotline. (Walker 2022)

Connecting victims with social workers allows immigrant human trafficking victims to be equipped with available information and resources regarding housing, transportation, education, childcare, employment, and immigration that may allow for a smoother transition into the new community. These resources may help immigrants build a sense of autonomy and stability, and may lead to greater levels of economic self-sufficiency. Through the U.S. Department of Health and Human Services (HHS), there are six major US welfare programs to which clients can be referred: Temporary Assistance for Needy Families (TANF), Medicaid, Supplemental Nutrition Assistance Program (food stamps), Supplemental Security Program (SSI), Earned Income Tax Credit (EITC), and Housing Assistance. Providers are encouraged to become familiar with these social services. Other services can be found through the Office of Trafficking in Persons (OTIP), a part of HHS which aims to prevent human trafficking though public awareness, providing better avenues for victim identification and assistance, and working to help rebuild the lives of victims. Furthermore, the U.S. Department of Justice (DOJ) has designed and implemented a number of programs intended to better identify victims, provide services for victims, train stockholders, and hold traffickers accountable. (Salami' 2021)

Experts stressed the need to be prepared to navigate the multiplicity and complexity of needs inherent in working with vulnerable youth. Stephanie stressed the following: Because of how intense this target population that you are looking to work with (is), are you considering other organizations that are working with this target population that can be that added support? (Bounds 2020)

The Grady Nia Project is a manualized, culturally informed, empowerment-focused psychoeducational group intervention that was designed to reduce suicidal ideation, depression symptoms, PTSD symptoms, and general psychological distress among suicidal African American women with histories of interpersonal trauma. The manualized group intervention consists of a weekly check-in and assessment of suicidality, psychoeducation related to intimate partner abuse, and creating a safety plan. The group adopts a womanist orientation with emphasis on spirituality, identity, community, and empowerment. Compared to treatment as usual, the Nia intervention has shown reductions in all targeted psychological domains and reductions remained at 6- and 12-month follow-up. Further research evidence suggests that existential well-being mediates treatment effects of Nia on suicidal ideation and depressive symptoms. (Bryant-Davis 2019)

Normalize mental health and substance abuse treatment… encourage the use of culturally sensitive treatment; identify and promote proactive mental health and substance use allies within intergenerational family networks; …support the use of family group conferencing in addressing mental and behavioral health within intergenerational family networks (Valandra 2018)

Provide her with resources that she may utilize immediately and/or in the future. Provide them in a form that she can keep safe, secure, and secret if needed. (Robinson-Dooley 2013)

Providers must also recognize the inherent risk of reintegrating their clients into their ethnic and/or religious communities... We encourage providers to be attentive to clients' safety concerns and to be curious about current relationships and potential contact with traffickers. Great care should also be taken when linking victims of human trafficking with community organizations, as many victims of human trafficking were trafficked by members of their own communities. Some victims continue to live in fear of being recaptured and punished for escaping, and choose not to socialize with members of their ethnic and/or religious community. Providers must approach this issue with caution and carefully find the balance between helping clients reduce their sense of social isolation and helping them decrease their fears and risks of being re-victimized. Finally, it should be noted that some victims have also been perpetrators of abuse and may be afraid

that group members will retaliate against them for their previous actions, or publicly identify them as perpetrators. Thus, therapists should take care to listen to their clients regarding such fears and provide referrals that will bolster rather than weaken social support. (Salami' 2021)

Provide culturally relevant supports that draw on appropriate knowledges… Commit to providing 24/7, flexible, and individually tailored support for several years for each trafficked person… Provide support that is relational and holistic. Building healthy relationships within families, communities, and between service providers and trafficked persons is key to support and healing. (Nagy 2020)

Culturally congruent womanist therapy with African American sex trafficking survivors addresses the consequences of trauma, while also attending to systemic factors, empowerment, expressive arts, spirituality, cultural strengths, and social networks. (Bryant-Davis 2019)

Subcategory: Cultural and linguistic adaptations

When possible, therapists should use treatments that have been specifically adapted to their client's ethnocultural group. For instance, Hays and Iwamasa provide a very useful handbook that highlights the utility of developing and using culturally responsive forms of CBT. They also elicited the help of experts in cultural psychology to describe the use of CBT with immigrants and specific ethnic minority groups... Researchers have also adapted CBT for use with traumatized refugee and ethnic minority populations with a high degree of effectiveness. (Salami' 2021)

Ending the Game is a manualized group intervention developed by an African American sex trafficking survivor. Attending to the experiences of the majority of African American survivors who come to trafficking not through force or fraud but coercion, this intervention focuses on uncovering the common psychological coercion tactics that traffickers use to manipulate and exploit survivors. The intervention encourages the use of a survivor coleader, with an aim on addressing psychological coercion, disrupting the bond with the trafficker, and enhancing a sense of empowerment through healthy identity development and healthy coping strategies. The treatment consists of psychoeducation and enhancing of insight, particularly for survivors who were taught by the trafficker to conceptualize their exploitation as economic empowerment. The effects of the miseducation on the survivor's sense of self, relationships, and future goals are explored. The outcome focus for this intervention is relapse prevention to counter the likelihood of survivors returning to traffickers based on cognitive distortions around the trafficker caring for them and/or economically empowering them. (Bryant-Davis 2019)

Participants also reported using ethnic-specific materials such as African American women's magazines during self-esteem groups, or taking girls on field trips to African American cultural centers. (Harper 2013)

With the goal of increasing the efficacy of prolonged exposure (PE), specifically for African Americans, Williams et al. developed a culturally adapted version of PE that incorporated race-related themes, such as racism, social support networks, faith-based coping, images of strength as potential barriers to trauma recovery, cultural mistrust, and racial and gender differences between the client and therapist, if present. Although there are no empirical investigations of culturally adapted PE for African Americans, case studies show promising outcomes. It is important to note that the Williams et al. PE adaptation was developed to target racism-related trauma. (Bryant-Davis 2019)

A research study about the prostitution and trafficking of 105 Native Women produced by Minnesota Indian Women's Sexual Assault Coalition and Prostitution Research and Education (2011) has found that... Many women expressed a need for counseling, health care, domestic violence shelters, rape crisis centers, homeless shelters, and substance abuse treatment centers that incorporated Native cultural traditions into the healing services provided (Robinson-Dooley 2013)

"The current medical model that is 'trauma informed' has been helpful helping medical organizations, nonprofits, and schools understand that trauma is a thing and we have to know how to recognize it. But, we still have a knee jerk reaction to send people to clinical environment. Sometimes that is a good thing, and sometimes it is not good because of the mistrust, lack of congruity with Indigenous values. How do we create responses that are healing informed, not trauma informed? These are traumatized people, and the best way to help them get settled and stay out of the workhouse, or get that job, stay in school, prevent diabetes … whatever it is, it's our job to connect them with culturally meaningful and

healing practices that can be normed in their families, peer groups and the organizations that serve them." (Advocate, key informant) (MMIW)

Our study adds to the existing literature on recovery by identifying specific structural and cultural factors that may need to be addressed when working with Asian women. Our results indicated that these services would ideally be provided in the survivor's primary language due to their limited English proficiency and programming would have to be flexible to accommodate women who may have full-time working hours. (Lim 2023)

Predominantly, what stands out about helpful services for these research participants was access to Indigenous based resources, including ceremonies. For the most part, Indigenous Peoples want services that are led by Indigenous Peoples, communities, and agencies based on traditional values, knowledges, and practices. (Olson-Pitawanakwat 2021)

Additionally, the use of culturally contextualized narrative therapy can empower African American girls and women who are survivors of both the historical trauma of slavery and its vestiges, as well as the contemporary reality of sex trafficking. The historical trauma of post-traumatic slave syndrome may be addressed in part by examining the narratives of slaves with attention to coping, resiliency, and resistance strategies that were adopted and may be adopted today. To address contemporary racism, Emotional Emancipation Circles have been developed for African Americans as support groups where African Americans gather to tell their stories of racial trauma, disrupt beliefs in White superiority and Black inferiority, and create new narratives of worth, value, identity, and creating healthy, affirming relationships. Along with historical narratives of enslaved Africans and contemporary narratives of racism, African American girls and women who exit sex trafficking may benefit from exploring the narrative of their violation and survival and the narratives of group members and more advanced survivors. The use of narrative treatment is congruent with African American culture as African American church tradition historically used testimony services in which members shared their stories of survival and triumph. Additionally, the African American hip hop psychology adopts the strategy of rapping in which the creation and dissemination of survival narratives is both culturally accepted and celebrated. Outcomes associated with the use of narrative interventions include a greater sense of community, increased sense of self-worth, value, and humanity, and decreased isolation and shame. (Bryant-Davis 2019)

Participants also reported using ethnic-specific strategies or interventions during service provision. For instance, participants reported informal and formal efforts to address ethnic awareness such as talking to girls or their caregivers about prejudice and discrimination... Many of these efforts were informal and addressed during individual or group services targeting other issues. For instance, one participant reflected: One of the things I always do with the parents is ask them to talk with their children about what is appropriate and not appropriate when stopped by the police… I think our parents need to say, "You know, this is what you do as a black person when a police officer approaches you." …Being able to tell them, "When you're competing against another race…you need to come in there five steps ahead" …knowing what's appropriate behavior for us. I tell our kids, "The same rules of engagement do not apply to all people." (Harper 2013)

In terms of useful evidenced-based treatments for treating the psychological sequelae of human trafficking, we recommend Cognitive Therapy, Cognitive Processing Therapy, Cognitive Behavioral Therapy, and Prolonged Exposure, especially those forms of therapy that have been adapted to the needs of specific ethnocultural groups. (Salami' 2021)

Project Prevent is a 6-week after-school prevention program, serving primarily African American preadolescent girls and aiming at fostering positive identity that counters sexualized role models that are depicted in the media. Attendees learn about critical thinking, healthy coping strategies, and media literacy with the aim of promoting positive images for the self, sexuality, and relationships. This program aims to use psycho-social positive advancement as a method to counter vulnerability to sex trafficking. The intervention is a manualized group treatment focused on restructuring cognitions to reduce African American girls increased risk for sexually transmitted infections and sex trafficking. (Bryant-Davis 2019)

It is more effective to address shame and guilt through interventions that are compatible with the background and beliefs of [Thai] MDS, such as empathetic listening… listening to the trauma stories of Thai MDS is crucial to their healing and recovery. To cultivate a safe environment in which a survivor feels safe enough to reveal her traumatic experiences, it is crucial to develop rapport through empathetic listening. By sharing their trauma history, Thai MDS no longer carry their burden alone. The effect of social support has healing effects. (Villareal Armas)

It is equally important to tailor treatment to the needs of ethnically diverse survivors in ways that address gender-related concerns. Therapy approaches for female sex trafficking victims should aim to empower women and girls; to accomplish this objective, attention must be given to improving body image and self-esteem and to developing adaptive coping strategies. (Bryant-Davis 2017 #2)

Indigenous healing paradigms help revive culture, while Indigenous harm reduction is a process of integrating cultural knowledge and values into strategies and services to tackle the effects of colonialism (Nagy 2020)

Additionally language barriers can limit an understanding of legal processes, and mental health professionals and language and cultural interpreters can play a critical role in facilitating informed, appropriate access to legal resources. (Bryant-Davis 2017)

Due to feelings of guilt and shame, it would be helpful to share with these women Thich Nhat Hanh's words: "Because we know that we can act differently, we do not need to feel guilt." Knowing that they can begin anew may also be helpful to alter the survivor's internalization of society's perception of them as sullied through sexual slavery. Hopefully, through these practices, these Thai women will gain insights, including that their enslavement was not their fault. (Villareal Armas 2010)

There is a dire need to develop therapeutic programs specifically designed for sex trafficking survivors. This may be accomplished by adapting already-existing models such as Prolonged Exposure (PE) in ways that are culturally syntonic. Developing and implementing culturally syntonic interventions would involve recognition of cultural variables such as language, socioeconomic status, and environmental factors. (Bryant-Davis 2017 #2)

...key principles to a trauma-informed approach... (6) cultural, historical, and gender issues, moving past racial, cultural stereotypes, and biases, offering gender-responsive services, supporting healing through cultural connections, recognizing and addressing historical trauma and historical narratives, and responding to the racial, ethnic, and cultural needs of individuals (Ortega 2022)

"agencies where I can do ceremonies and I still talk to my therapist." ... Every time I do one [of my traditions], it helps me heal myself… being two-spirit in the city, I can attend the female circles and the male circles and have that balance. (Olson-Pitawanakwat 2021)

integrating Indigenous approaches into the solutions (Olson-Pitawanakwat 2021)

Interest-based services. Some providers discussed interest-based services to address culture. Interest-based services referred to incorporating girls' main interests during direct service provision. For instance, one participant shared: I think the content and the materials that I use with the girls are very much age-appropriate…and it reflected their interests. When I realize how much these girls are into media and how much media impacts these girls, that's how we chose to work with these girls, using what they enjoy…If they enjoy looking at an iPad or you're pulling up something on YouTube, that's what you do if you want to be able to reach them. If they enjoy looking at a magazine that's for them and written by them, that's what you need to present. (Harper 2013)

Antiracist Interventions for Healthcare

Individual/interpersonal level interventions

Subcategory: Critical Reflection

Cultural awareness in working with survivors of trafficking entails self-awareness and an examination of personal biases, such as those concerning race and ethnicity, and the tendency of clinicians who deny or minimize the realities of privilege and oppression to avoid topics such as racism, classism, sexism, and homophobia as they may elicit painful or difficult affect for the client and for the therapist. This self-reflection is necessary and cannot be replaced by any amount of clinical knowledge. (Bryant-Davis 2017)

Oftentimes, clinicians are unaware of their own biases. Clinicians working with sex-trafficked individuals should commit to ongoing work to recognize and address their own implicit biases. Clinicians may consider starting by taking implicit association tests; tests validated to demonstrate where clinicians may have implicit biases. While more research must be done, some practices at reducing bias show some early promise. These strategies include exposing oneself to counter-stereotypical exemplars and ways to identify with the out-group. As clinicians become more aware of their own biases, they will become better equipped to work with a diverse population. (Ortega 2022)

be cognizant of implicit bias and the degree to which race, ethnicity, or gender may be impacting interactions or decision making. (Rothman 2018)

Additionally, the results of this study support a need for providers to further examine their own biases related to ethnicity, socioeconomic status, gender, and so forth and how these biases may perpetuate negative stereotypes and expectations. For instance, some participants' language concerning gendered aggression (e.g., girls "wanting" to fight boys or "allowing" boys to be physically aggressive with them) suggests possible gender biases that may warrant further exploration. (Harper 2013)

We argue that it is vitally important for service providers to continually assess their strategies and policies as well as to evaluate their personal biases and privileges. (Nagy 2020)

Examining personal biases. Some participants (n = 3) discussed examining personal biases (level 3) as a cultural facilitator to meeting girls' needs during direct services. For instance, one participant reported: Ethically, we are bound to be aware, you know, of different cultural, the whole term "cultural competence" and ethically we are also bound to be aware of what makes us uncomfortable as mental health providers and to know when we may need to bow out of a particular situation because our values conflict with the particular girls that we might be working with. Similarly, another provider reported: It would have been very, very easy for me to act totally disgusted when we were showing them some music videos because we let the girls choose which videos they wanted to look at. I mean, it's just important for me and for my intern not to be judgmental, but to be a facilitator—to help them develop a better understanding of their thoughts and actions, not to impart my beliefs and to change their mind about something. (Harper 2013)

With all diverse populations, it is essential for practitioners to examine their own biases and personal reactions with regard to race, immigration, sexual orientation, gender, age, religion, and ability status. (Gerassi)

Moreover, cultural humility and responsiveness give us a frame for working with all populations. This frame involves taking an attitude of lifelong learning that includes frequent self-reflection, evaluation, and critique. (Ortega 2022)

....committing to a lifelong process of recognizing and minimizing one's own implicit and explicit biases, (Ortega 2022)

Also, it is important that providers are aware of their potential implicit bias and how it may impact the care that they provide. (Ortega 2022)

In order to be effective in working with diverse populations, Sue and Sue encouraged clinicians to gain self-knowledge. They explained that understanding individuals from diverse backgrounds requires book learning, self-exploration, and more interactions with multicultural people. Through this independent study, clinicians will most likely experience intense feelings, since the topic of diversity usually elicits strong emotions. Sue and Sue advocated for acknowledgement of what arises through introspection and other explorations. They also emphasized the importance of open dialogue in developing cultural competence. (Villareal Armas 2010)

Subcategory: Education on care models

Further, there is a need to build professionals' capacity to co-construct and implement creative, context- and culture-specific strategies and interventions for low-income African American girls in high need urban settings in the face of multiple barriers through explicit training. Providers will need a model for school-based mental health interventions that facilitates practitioners' ability to collaborate with members of the local culture (e.g., teachers, other mental health

professionals, and students) while constructing cultural knowledge for use during service provision. Existing models for developing and implementing participatory culture-specific school-based mental health interventions can enhance professionals' capacity to implement cost-effective, efficacious, and sustainable collaborative mental health programs in high need urban schools. As a result of limited time and resources, strategic planning for professional development and collaborative efforts will be important. (Harper 2013)

Given the predominantly white social service workforce and overrepresentation of people of color among people who are at risk of sex trafficking, an effort to integrate critically conscious, AOP approaches, particularly among white providers, is crucial. (Gerassi)

Overall, education on trafficking needs to incorporate the knowledge and experiences of the people most affected by trafficking and anti-trafficking, including those who endure the collateral damage caused by anti-trafficking interventions in diverse labour sectors, not only the sex trade. (Fukushima 2021)

...and a need to equip providers with strengths-based strategies for enhancing girls' resiliency and resistance. (Harper 2013)

Improvement of training in trauma and transculturally informed models of care… (Davidtz 2022)

Educate health professionals… on… the Health, Stigma and Discrimination framework, and the rights-based approach to patient or client care… Educate health professionals on rights-based and trauma-informed care… (Wallace 2022)

destigmatizing interventions… could include increasing trauma-sensitive and trafficking-specific training for providers. (Wallace 2022)

This toolkit not only touches upon relevant knowledges and necessary programs, but also highlights especially the importance of experiential and Indigenous knowledges… the importance of having culturally sensitive trauma and violence-informed training. (Nagy 2020)

Subcategory: Education on Social Context and Oppression

For example, "In Their Shoes" is a simulated training experience for intimate partner/teen dating violence, which allows participants to walk through the choice points for economically disadvantaged survivors. The discussion guide instructs facilitators to "confront oppression in the group" by helping participants to understand that "the dynamics of oppression that support and uphold domestic violence, economic injustice, racism, xenophobia, able-bodyism, heterosexism, and all of the ways that privileged groups maintain their power and control over others." Sex trafficking education must implement similar approaches... (Gerassi 2018)

Racial biases that disadvantage black CSEC survivors persist, so it is imperative that professionals who serve or treat CSE youth receive training on the consequences of systemic, institutionalized, and individual racism. (Rothman 2018)

It is important that this training also foster an understanding of such key terms as "power," "privilege," "racism," "sexism," "heterosexism," "oppression," and "stigma" (Bryant-Davis 2017 #2)

It is also vital that the history and present impacts of structural racism, anti-Blackness, colonialism, and the intersecting hierarchies of citizenship status, class, caste, gender, sexuality, and ability are acknowledged and addressed as part of the work of anti-trafficking education. (Fukushima 2022)

Educate health professionals… on human trafficking dynamics, harmful effects of stigma and bias, cultural factors affecting views on human trafficking, mental and medical health, and health care… health effects of stigma, bias and discrimination. (Wallace 2022)

Biases, assumptions, and stereotypes about victims from marginalized communities and immigrant populations may hinder identification. Thus, psychoeducation and training are crucial to help dispel misconceptions. (Salami' 2021)

Health professionals should be trained on the diversity of people who experience trafficking, especially counter-stereotypical examples of trafficking, in order to combat structural and institutional biases that impact identification and intervention efforts for this population. (Prakash 2022)

...it is essential that sex trafficking... trainings address race and racism (Gerassi 2018)

Specifically, therapists need to be trained to identify intersectional identity markers for themselves and for the African American girls and women they serve. (Bryant-Davis 2019)

This training would be ongoing and bidirectional and would include psycho-educational components on human trafficking and its social and individual consequences, as well as components that describe the context of intersecting identity markers, such as race, ethnicity, gender, and socioeconomic status. (Bryant-Davis 2017 #2)

destigmatizing interventions… could include… educating professionals at large on the social and cultural factors that influence stigmatization. (Wallace 2022)

Understanding human trafficking and violence against women in general necessitates a framework that makes central the intersection of gender, race, class, and sexuality and how it perpetuates the victimization of women. Such a framework would facilitate the development of culturally and gender-sensitive training for judicial and mental health professionals. (Bryant-Davis 2017 #2)

…education is needed on legal rights and arrests processes. (Lim 2023)

it is essential that sex trafficking... trainings… implement an AOP practice approach that is tailored toward the racial identity of the provider (i.e., language used to address race may differ for a provider of color as compared to a white provider). (Gerassi 2018)

Modern Slavery Training Standards Framework and training curriculum for health professionals should include mandatory training on cultural intelligence, intersection of trafficking, race, religion, disability, gender and sexuality, equality, diversity and inclusion. Design and delivery of specialist events exploring country/culture/community specific modern slavery and human trafficking topics of benefit to health practitioners and wider partners. (BASNET)

Subcategory: Expanding basic education on trafficking

…advocates also recommended training for school nurses, noting that survivors often have multiple doctors/hospital without any health professional screening for DMST by asking the right questions and knowing what to look for during the visits. According to the advocates, training for school nurses should include noticing sudden or subtle shifts in appearance over time, such as branding tattoos, bruising, and signs of addiction. (Walker 2022)

– Provide training to law enforcement and emergency room staff about providing trauma-informed care for Indigenous women and girls who have experienced violence and sexual assault, including working with victims of trafficking and those who are being exploited by their own relatives or caregivers. (MMIW)

Providing training programs in collaboration with school personnel, first responders, and other community stakeholders about indicators of trafficking and how best to intervene will enhance clinicians' ability to identify and treat persons who have been, or are being, trafficked. (Salami' 2021)

Modern Slavery training should be included in mandatory safeguarding training for all NHS staff... All health professionals should undertake 2 yearly training on Modern slavery and human trafficking focusing on cultural intelligence, equality, diversity and inclusion... Monthly delivery of specialist events and workshops accessible to all health practitioners. Attendance based on learning needs aligning with client/user base. (BASNET)

There is also a need for training on specifically what DMST is, so that they can both educate young people about it, as well as know the warning signs. (Walker 2022)

Systems like law enforcement, health care, child welfare, schools, jails/prisons and re-entry, and housing often do not intervene adequately to prevent sex trafficking or to support Indigenous people to safely exit that life. Providers in these fields need better training on how to recognize and appropriately address sex trafficking. (MMIW)

Pediatrician/medical profession [recommendations from advocates]: ➔ Train school nurses, ER, school psychologists on red flags from a health perspective. (Walker 2022)

School Counselor Training. Four of the advocates recommended the need for more specialized training for school counselors around the crisis of DMST. They cumulatively suggested the following as need for training for school counselors: ● To be able to teach young people through seminars about what safety looks like and how to be more aware of their surroundings ● (Along with administration) How to handle situations that don't sexualize, criminalize, and/or unnecessarily push especially girls into the child welfare or juvenile justice system. The school counselor advocate shared the necessity of having those hard conversations both in Master's programs and in school-level policies/training. ● Intersection of teen dating violence & sex trafficking (One advocate shared finding from a particular study that stated 80% of school counselors had never had any training in teen dating violence, even though 60% of girls reported experienced abuse/violence in their relationships) (Walker 2022)

Disinformation spread in this manner [co-opting] additionally presents changes for anti-trafficking educators who must respond to these efforts without providing conspiracies a larger platform and more legitimacy. Unfortunately, there is a wealth of evidence in rumor psychology to support that increased exposure afforded to disinformation, even if successful in discrediting, can mean inaccurate beliefs "still tend to persist, but in a weakened state." In other words, so long as the QAnon beliefs or conspiracies are commented upon, they have the potential to gain traction and be integrated within the audience's mental models regarding human trafficking as well as our sociopolitical climate. (Prakash 2022)

Clinician educators should avoid sources that incorporate sensationalized language within discourse regarding human trafficking (e.g., avoid imagery of victim in restraints). Information alleging complex conspiracy theories without supporting evidence should be avoided. Medical educators must also be cautioned against using information disseminated by organizations that (1) promote racial, ethnic, or religious stereotypes and (2) claim to "rescue" trafficked "victims." (Prakash 2022)

Health Systems Interventions

Subcategory: Representation & diversity

Seventy-four percent of the clients Love146 has provided direct services to are children of color. As a field, it is important to acknowledge that most of the providers working with this population are white... As professionals, we must also proactively work to attract, recruit, hire, and promote staff who reflect the population we are serving. (Williamson 2020)

An additional group of experts expressed their perceptions about the potential challenges associated with engaging African American youth in the STRIVE intervention: Veronica: I think you need therapists that are African American. I mean I think that would go over better with families. Again it decreases the stigma that they are working with someone who is African American and is in a healing profession. It would decrease some of the worries of being involved in a research study and distrust of researchers in general. (Bounds 2020)

Some providers reported gaining valuable knowledge related to working with low-income African American students during practical experiences. For example, one provider discussed her experiences related to observing other professionals set high expectations for socioeconomically disadvantaged students: "Just to see African American women in the role…whether they were psychologists or educators, working in a low socioeconomic area, not accepting poverty, not accepting that as saying, “you can't do it.” I didn't see that at all. I just saw expectations all the way; and that was very much important to me, setting up expectations for students…the climate." (Harper 2013)

Address systemic racism in all systems that interact with Indigenous women and girls (education, health care, housing, child welfare, law enforcement, criminal justice, etc.) by hiring more Indigenous staff… Create employment pipelines for Indigenous people to enter careers within the systems of education, health care, housing support, child welfare, law enforcement, and criminal justice. (MMIW)

At the most basic level, the offices of judicial employees should represent diversity in staffing and leadership positions and should utilize art, magazines, books, and supplies for clients’ children that are culturally diverse and reflective of the various backgrounds of the survivors. (Bryant-Davis 2017 #2)

Increasing diversity of providers is helpful in providing diversity of perspectives within one environment, particularly among services that target minority populations. (Gerassi 2018)

First, it is vital that those with lived experience, from a diversity of demographics, shape health sector efforts on trafficking. (Prakash 2022)

Subcategory: Critical reflection

Mezzo-level intervention strategies also need to include the systemic review of organizational policies, practices, and processes within agencies that offer sex trafficking–specific services to identify potentially racist and oppressive practices that might act as a barrier for African Americans in accessing and engaging services. (Valandra 2018)

The principle of disciplinary self-critique encourages DMST researchers and practitioners to question the norms and accepted practices in the anti–sex trafficking field that perpetuate inequity. One way to practice disciplinary self-critique is for researchers to be reflexive about existing racial power dynamics and to question who holds the power in determining the research agenda, program questions, and the interpretation of the results. (Cook 2022)

Systems looking to create programs to care for trafficked individuals may consider embarking on a Racial Equity Impact Assessment (REIA). Race Forward: The Center for Racial Justice Innovation has created a toolkit to assist organizations in undergoing a REIA. An REIA systematically evaluates how a proposed program may impact racial and ethnic groups. (Ortega 2022)

Examine how institutional level factors... perpetuate the cycle of violence and trauma for Black girls (Cook 2022)

Examine current DMST theoretical frameworks for the presence or absence of antiracism principles (Cook 2022)

It is important for anyone taking up the work of anti-trafficking education to reflect on why they or their organisation is interested in teaching about trafficking, what is given and gained by specific educational content, and what opportunities and constraints exist for ensuring an empowering and ethical experience for all involved... What does it mean to take on a ‘new’ population for care when medicine, science, law, and other institutions of biopower are also sites of control and violence? (Fukushima 2021)

Another principle, critical approaches, challenges researchers and practitioners to move beyond the status quo of anti-trafficking efforts and to investigate how personal and institutional biases affect knowledge and knowledge production. An example of how critical approaches are applied in DMST can be seen in an article by Gerassi, in which the authors—a researcher, an organizer, and a practitioner—provide critical reflections on their discourses, assumptions, and actions,

especially naming the unacknowledged bias that was occurring in an anti-trafficking task force in both leadership and service delivery. (Cook 2022)

Identify and challenge personal, institutional, and societal racial biases and how they influence research, practice, and policymaking (eg, Black girls do not fit the purview of DMST victims) (Cook 2022)

Subcategory: Inclusive Organizational Culture

Create an organizational culture that does not tolerate bias and discrimination toward patients, and implement a system that allows anonymous patient or staff reporting of such behavior. (Wallace 2022)

It is also important to take action when witnessing bias and discrimination in the health-care setting, to protect patients and staff, and to eliminate any actions that can foster a culture of intolerance and stigmatization. (Greenbaum 2021)

All systems: Accountability: not turning a blind eye from the biggest to smallest situations that silence Black and Brown girls/women (Walker 2022)

By understanding, valuing, and incorporating the cultural differences of America's diverse population and by examining one's own health-related values and beliefs, health care organizations, practitioners, and advocacy groups can support a health care system that responds appropriately to, and directly serves, the unique needs of populations whose cultures may be different from the prevailing culture. (Robinson-Dooley 2013)

Address systemic racism in all systems that interact with Indigenous women and girls (education, health care, housing, child welfare, law enforcement, criminal justice, etc.)... by providing training and education to reduce bias among professionals working in these systems, and by demanding accountability to eliminating bias. (MMIW)

Subcategory: Inclusive Policies

Redesign organizations policies, procedures to center survivors material needs and experiences of trauma, doing away with strict rules and policies that are pathologizing, infantilizing, and replicate patterns of power and control while excluding the most marginalized survivors. Ensure that your organization or health center has fair labor practices, pays a living wage, and is trauma-informed for staff. (Larson 2023)

Health systems should develop policies that identify and respond to individuals who have experienced any form of human trafficking, inclusive of labor and sex trafficking. (Prakash 2022)

Similarly, addressing all forms of structural oppression to achieve health equity requires envisioning new systems and structures rather than simply trying to get more people access to historically oppressive systems. Because oppression creates different circumstances for different people, health equity is inseparable from racial and gender justice. (Larson 2023)

GP practices should commit to taking steps to tackle the barriers faced by migrants from ethnic minority backgrounds in accessing healthcare... Everyone living in UK should be registered with GP regardless of immigration status. (BASNET)

Remove all barriers to free access to NHS health care including charging and information sharing with the Home Office for all. This is to allow access to healthcare for all victims of modern slavery and human trafficking, whether recognised or as yet unrecognised... Reversal of charging regulations and stop sharing information with Home Office… (BASNET)

Systemic changes must also occur to improve the cultural responsiveness of care for individuals experiencing trafficking…

Work must be done to eliminate structural racism embedded in existing mental health services

Recruit more interpreters and make better use of existing interpreting services to help health practitioners better spot and understand the plight of potential victims of Modern Slavery and Human Trafficking. (BASNET)

Improve provision of free specialist culturally appropriate, trauma informed services for all survivors of modern slavery... [goal:] All survivors of modern slavery and human trafficking are able to access timely, appropriate and free specialist trauma informed services. (BASNET)

The NHS should launch an annual target to end race disparity within the service. (BASNET)

I would add guidance counselors that I guess are more involved and easier to contact, (Walker 2022)

Moreover, decolonizing approaches not only recognize the harms of settler colonialism but systematically work toward repair and redressing such harms. For example, this could mean working toward the implementation of the recommendations of the Truth and Reconciliation Commission of Canada (2015) and the National Inquiry on MMIWG2S (2019). (Nagy 2020)

Expand access to Sexual Assault Nurse Examiner (SANE)/forensic exams in tribal and rural areas; consider more regional partnerships across health systems and with tribes to expand access to nurses with this training and expertise. (MMIW)

Healthcare Provider Role Beyond Healthcare Provision

Research, Intervention Design, & Evaluation

Subcategory: Research Deficits

Although African American girls and women are at increased risk for sex trafficking domestically, there has been little research focused on the development and provision of culturally congruent care. (Bryant-Davis 2019)

Next steps should include further exploration of intersecting stigmas and testing of stigma-based interventions by measuring stigma reduction and psychosocial, mental, and physical wellbeing. (Wallace 2022)

Since CSE is a common risk for homeless youth, the paucity of data on the effects of CSE on the mental health of African American and sexual/gender minority children and adolescents reflects a significant scientific gap (Bounds 2020)

Despite the frequent use and effectiveness of cognitive therapies in the treatment of various disorders among European Americans/Caucasians, we recognize that there is limited research on the effectiveness of empirically supported treatments among immigrant populations. (Salami' 2021)

Overall, very few programs or interventions for sex trafficking survivors have been formally evaluated, especially for those who were internationally trafficked, and future research efforts should aim to establish their efficacy. (Lim 2023)

The PHCRP framework encourages practitioners to critically evaluate the types of data and methods needed to assess these complex relationships. In other words, if we are to take seriously the measurement and conceptualization of intersectionality as it relates to Black girls' vulnerability for DMST, then we must start by collecting data on race and ethnicity, in addition to data on gender and other relevant constructs. Hospital-based anti-trafficking programs that

implement a multidisciplinary approach to health services are uniquely positioned to collect intersectional data encompassing both medical and social information… (Cook 2022)

Moreover, as noted by Williams et al. (2014), most PTSD assessment measures have not been validated with primarily African American samples; therefore, it remains unknown whether existing measures accurately capture PTSD in minority populations. Validation of assessment measures with African American samples is an important avenue for future research. (Bryant-Davis 2019)

At the onset, the authors acknowledge that psychologically focused human trafficking research is quite minimal, and that within this limited literature there is a dearth of attention given to the intersection of racism, ethnic bias, and human trafficking. It is worth noting that the lack of empirical data concerning these issues parallels the experiences of racially, ethnically, and socioeconomically marginalized women more broadly in psychological research. (Bryant-Davis 2017)

With regard to racial and ethnic demographics and human trafficking, very little discourse has occurred and even less research exists. (Rollins 2017)

future research should explore how these dynamics emerge in other geographic contexts, particularly in locations that are more ethnically and racially diverse. (Gerassi 2018)

Future work should use an intersectional approach to examine how providers address race, gender, sexual orientation, and (dis)ability status, for people with intersecting, oppressed identities. (Gerassi 2018)

…there is a paucity of literature on the interface between race and treatment outcomes for PTSD… (Bryant-Davis 2019)

…more research evidence is needed to enhance understanding regarding the effectiveness of mindfulness-based interventions for PTSD... Given the paucity of studies using MBSR with predominantly African American women with histories of interpersonal violence, it will also be important for future research to examine the efficacy of MSBR in this population… research is needed to examine the extent to which this culturally adapted version of PE will result in positive outcomes for PTSD related to interpersonal trauma… Future randomized controlled trials with predominately African American women samples are needed to more fully understand the efficacy of MBSR and promising culturally adapted interventions for African American women who have histories of interpersonal trauma. (Bryant-Davis 2019)

Future work should examine the extent to which such differences in practice approaches [critically conscious, AOP practices] increase positive outcomes of social service engagement, particularly among clients of color who are at risk of sex trafficking. (Gerassi 2018)

Limitations of these recommendations include the minimal literature on which they are based and the lack of evidence-based systematic, randomized control studies. These promising practices [culturally adapted, mindfulness, expressive therapies] warrant the attention of researchers and program developers to fulfill the aim of providing equitable, effective, and ethical care of African American girls and women. (Bryant-Davis 2019)

...the literature on effective responses to sex trafficking is minimal, and the role and dynamics of culture have largely been ignored, thus leaving a major gap in research-based knowledge and guidelines for psychological and legal practice with sex trafficking victims. (Bryant-Davis 2017 #2)

...and future research should evaluate the extent to which such trainings [similar to "In Their Shoes"] impact providers' perceptions of racism in their work and actions taken to address structural oppression in their work. (Gerassi 2018)

...future work should explore the perspectives of providers of color who work with people at risk of sex trafficking, specifically about their perceptions of critically conscious, AOP practice and any resulting challenges in working with white colleagues. (Gerassi 2018)

Monitor support for policies that call for specific protections for survivors to help decrease inequities. (Wallace 2022)

[Health Stigma and Discrimination Framework] Health and social impacts: conduct longitudinal studies to evaluate the health and social outcomes before and after multi-level stigma reduction interventions. For example, mental illness and physical health diagnoses, accessible and appropriate care, disability and quality-adjusted life years, life expectancy, employability, education attainment, quality of life, family and social community relationships, etc. (Wallace 2022)

Psychologists could help even more by developing and systematically researching intervention strategies, such as the mindfulness practices discussed in this chapter. (Villareal Armas)

Expand research on the multi layered interactions of stigma related to human trafficking and its measurement (Wallace 2022)

Researchers should follow the standards of documenting the racial and ethnic background of their programs as well as attend to any cultural modifications, limitations, or observations that are made in developing and evaluating programs serving survivors of sex trafficking. (Bryant-Davis 2019)

The availability of public data on identified sex trafficking victims and relevant statistics depends on a range of factors. For instance, agencies and organizations may collect data that is not organized or disseminated for researchers or community audiences to access. Data that is published is not always gathered consistently or presented as all-inclusive. Notably, diversity is not exclusively addressed by solely distinguishing one's gender, race, and age, as most commonly recorded in studies. Additional aspects of an examinee's identity, such as their ethnicity, religion, spiritual beliefs, immigration status, LGBTQ+, and socioeconomic and disability demographics, are to be investigated. (Davidtz 2022)

Identify ways to measure stigma to design effective prevention strategies. (Wallace 2022)

Subcategory: research impact

One of the first steps in community organizing or coalition building is gathering information to define the problem's scope and identify available resources and potential barriers to change... Early on, each agency identified what information they could bring to later meetings to get a more comprehensive view of this number. For example, the child abuse clinic's advanced practice nurse and the university-based public health nurse researcher partnered to review all the clinic's extra-familial sexual abuse cases over the previous 5 years, documenting the increase and comparing abuse experiences and health needs of Hmong runaway girls with non-Hmong girls seen at the clinic. They presented a summary of the results at a Task Force meeting, providing the group with compelling data about the scope and severity of the problem, which served as the basis for a number of HYTF actions. (Saewyc 2007)

We also heard multiple discussions about the lack of human trafficking data specific to our region that might help secure funding. Yet, some participants also worried that lower statistics could be used against Northeastern Ontario funding applications. (Nagy 2020)

Both youth and content experts stressed the importance of considering the historical contexts of research in the African American community which is magnified when the focus is on the topic of mental health and wellbeing. (Bounds 2020)

There are several ways in which psychologists can expand their role to participate in advocacy practices specific to the needs of sex trafficked women in the judicial system. One of the critical steps of advocating on behalf of sex trafficked women is to investigate the interplay of trauma histories and contextual and historical factors (e.g., age, ethnicity, setting, racism, sexism), all of which have an impact on well-being. Research that examines these factors is critical to identifying the specific needs of the population. Once this information is collected, psychologists can disseminate information to leaders and partner with organizations that meet the needs of the specific interest areas. Specifically, psychologists should strive to provide and interpret data in a manner that demonstrates urgency for change, collaborate with meso-level stakeholders to develop a vision for implementing change, and develop a detailed plan for implementing the change process. Lastly,

psychologists and stakeholders should be mindful of macro-systemic barriers and anticipate resistance. (Bryant-Davis 2017 #2)

The research partnership between the university public health nurse researcher and child abuse clinic nurse generated verifiable empirical evidence. This helped secure grant funding, provided information for community organizations and the educational video, and served as a catalyst for the local newspaper series. Results have been disseminated at national and international conferences, and were recently published in professional journals. (Saewyc 2007)

Subcategory: Holistic interventions

In line with previous studies that have identified various service needs for sex trafficking survivors in the United States, our study results suggest that the following services would be needed to facilitate survivor recovery and well-being: trauma-informed and culturally appropriate services that provide or promote community building, legal assistance, medical care, life skills and employment training, and affordable housing. (Lim 2023)

The mental health system may consider whether less restrictive and culturally responsive community-based services are available for young people like Kerry. For example, a race-concordant outpatient provider delivering intensive case management services, peer mentorship services, and peer support groups focusing on distress tolerance, symptom self-management, racial injury, healthy relationships, education, and job skills may have been beneficial. (Ortega 2022)

Youth reinforced how we must be ready to assist in meeting their survival needs, as highlighted in the dialogue below: Interviewer: Let's talk a little bit about young people who had to trade sex to survive. You end up sleepin' with somebody 'cause you needed a place to stay, you needed somethin' to eat, you needed a ride, whatever the case might be...Could STRIVE help that person? Youth 1: Could it? If I was trading sex for a ride, would you be able to give me a free ride? If the answer is yes, then sure. If the answer is no, then I doubt it. (Bounds 2020)

An example of Indigenous women taking up this work is the Ginoozi ("She Is Tall" in Anishinaa-bemowin) Sexual Violence Response Team (GSVRT) at the Native Women's Resource Centre of Toronto (NWRCT, n.d.). The first of its kind in Canada, GSVRT uses a wholistic or wrap around approach to assisting women, girls, two-spirit, and trans women who are, or have been, trafficked. Helpers provide culturally safe services and preventative strategies that address the high risk of sexual violence and trauma that Indigenous women, girls, two-spirit, and trans women face. ...GSVRT's blanket of services includes referrals to trauma therapy, addictions support, health care, access to Elders, traditional healers and ceremonies, housing, food, clothing, and personal care. All of these institutional supports provide a safe space and wholistic services that create a path for healing for women, girls, two-spirit and trans women who have experienced trafficking... the findings from the research that this article is founded on clearly illustrates that NWRCT is of help to the participants as it was the only agency referred to as helpful in the interviews. (Olson-Pitawanakwat 2021)

Getting needs met was central for the youth who participated in our study. Melrose notes that entry into the commercial sex trade is often predicated around the concept of meeting unmet financial and employment needs. We would be remiss to ignore the social and economic context of risks associated with homelessness and sexual exploitation in our prevention efforts. (Bounds 2020)

was leading during the time of this study that focused on girls' social and economic issues: You have to intertwine in everything... I'm teaching girls how to crochet… What I had to do is I have to get them to see how crocheting items, the items can either be things that they wanna wear or gifts that they wanna give…or start charging…a business… (Harper 2013)

One participant described a past afterschool group mentoring program that she founded in collaboration with the school counselor, the school nurse, and the home economics teacher: We would have speakers to come in…a hairstylist owning a business…a fitness person…we had etiquette…we talked about what's appropriate and not appropriate for relationships with the opposite sex… We served as mentors to them… If a particular girl had something going on at school, they were supposed to come to us. We also kept tabs on their grades, we checked with their teachers to see if they saw any improvement, we had like little checklists that they had that teachers would fill out…we do a lot in our mentoring group with career flourishing… (Harper 2013)

Project Rose is also designed to empower victims through the provision of wrap-around social services that assist the participants in creating new, healthy, and legal options for socioeconomic independence. Victims are given referrals for housing, substance abuse, and medical and mental health treatment; they are connected with a mentor who has successfully exited trafficking for at least a year, and with a police officer who orients them to the program. More than three hundred women, between the age of eighteen and fifty-eight, have participated in Project Rose since its inception in 2011. Only 9 percent were rearrested at twelve months' follow-up, and participants in Project Rose were as likely as incarcerated women to show up to court. Critiques of Project Rose note that the program still threatens women with incarceration, which is disempowering. (Bryant-Davis 2017 #2)

Considering persons who have already been trafficked, Contreras and Farley note that only 14 states have programs specifically focused on serving commercially sexually exploited women. Given that African American women are among women most vulnerable to mass incarceration, it is important that therapeutic support for exiting trafficking is provided for them instead of criminalization of those who are most vulnerable due to racism and socioeconomic barriers to empowerment. Programs that address the underlying factors that keep women trapped in sex trafficking include residential care, case management, education and employment services, mental health counseling, addictions counseling, and mentoring by formerly trafficked women all in one site. Project Rose is one such diversion program that helps women escape the cycle of trafficking and incarceration by providing supportive police officers, food, clothing, housing, medical and mental health care, drug detox services, and a survivor mentor who had exited trafficking for at least a year, all of which have been found helpful to recovery. The study found that without the courts criminalizing the women, the women still completed the program and gained the benefit of having police officers who were trusted service providers instead of the traditional role of experiencing all police in a conflictual manner. (Bryant-Davis 2019)

Given the clear need for care coordination and new services, the clinic developed a home visiting and case management service and received grant funding to support it. HYTF members have since gained additional funding, allowing establishment of empowerment groups, family support, early intervention with runaways, and multiagency collaboration. (Saewyc 2007)

Programming priorities include having a dedicated case worker, Indigenous healing and wellness, mental health and addictions, peer outreach and support, and safe shelter and affordable housing. Our francophone participants also highlighted having French programming and services as culturally important, and that it is difficult for francophone clients to translate traumatic emotions into English. We further note the importance of supports for "aging out" youth and helping trafficked women get their children back. (Nagy 2020)

...it is important to integrate culturally responsive healing into system responses... Prominent ideas suggested by the experts we interviewed included peer-to-peer support for women who are being trafficked and mentorship programs for youth by community elders... "...we have parent mentors in our office who are women who have experienced the child welfare system and are now in a place where they are healthy and able to provide hope to women we work with. They have seen it, they share stories, and both have experienced extreme situations of violence. They have lived that trauma and are raw about the reality of it, but also have found a way to heal and move forward. Healing is not just linear; many things cause pain and create step backs, but they are committed to a path. Those relationships matter... Our model has shifted and is working" (Child welfare, key informant) (MMIW)

Finally, some participants expressed a need for collaborative services focused on caregivers. For example, one provider stated: I think, empowering the parents for education…helping them get the GED or the high school diploma, and then getting them some job training… Then they're not just in the community…now you get to interact with professional people, and now you begin to learn the culture outside of your community. (Harper 2013)

Subcategory: Co-creation

...Focus on supporting those who have experienced harms and violence and ask for help. Don't assume to know better than trafficked persons what their unique needs are at any given time… Involve persons with lived experiences in the paid circle of care. This includes in the design, management, and evaluation of programs, as well as community outreach and peer support. (Nagy 2020)

The principle of voice, applied to DMST, is needed to address the lack of young Black female voices in DMST initiatives. Researchers need to ensure young Black females are given opportunities to participate in designing and evaluating policies and services. (Cook 2022)

Joy, the school counselor, recommended not only providing young people with support and activities, but listening to what they need in all these areas ("academically, socially, emotionally, arts") and giving them opportunities to co-create them. (Walker 2022

Interventions should incorporate quality control and honor the voices of survivors by incorporating ongoing program evaluation by the participants. (Bryant-Davis 2019)

As with all violence prevention and public health work, it is essential that those closest to the problem are meaningfully involved in or leading program development, policy advocacy, and evaluation. We subscribe to the disability justice concept of "Nothing About Us Without Us." In exploring and experimenting with public health opportunities outlined in this section, practitioners, with and without lived experience, should ensure that people in the sex trades and survivors of human trafficking are at the decision-making table and that work is done in collaboration and reciprocal partnership. (Larson 2023)

Establishing a youth advisory board for ongoing consultation, partnership, and accountability. In order to ensure that youth voices are heard, we intend to create space for youth involvement by developing a youth advisory board. Community advisory boards are foundational to community-based participatory research. Our findings suggest the need to have a continued connection with the youth we serve to ensure transparency, relatability, and cultural humility. (Bounds 2020)

This approach [wraparound care including healthcare] came out of consultations with 40 community members including families, caregivers, survivors, those with lived experiences of being trafficked, and traditional Knowledge Keepers to ensure that the program fits the needs of those impacted. (Olson-Pitawanakwat 2021)

In order to develop effective and appropriate strategies, the paid involvement of persons with lived experience in the collaborative network is crucial, i.e., survivor-champions, sex workers, or family members. The idea of nothing about us without us is key to developing policy frameworks and frontline supports, including peer outreach and support, that meet the needs of individuals in a manner that respects their autonomy, self-determination, and empowerment, and is without judgment. (Nagy 2020)

According to the advocates, safe spaces also look like giving them opportunities to process what is going on in the world around them "and then us as adults being quiet enough to listen" and even further implementing those things they want and need–whether it's a student-run fashion show, a girls group, after-school program, talks on entrepreneurship, or anything else. (Walker 2022)

Cyril et al. identified how mutually beneficial relationships and the bi-directional learning that occurs with community advisory boards can improve the health of vulnerable populations. The iterative process of engaging with community advisory boards is not only valuable to vulnerable populations, it is also beneficial to the members of the clinical research team. Community engagement on this level has been identified as not only a way to amplify voices in marginalized populations but also a way to combat health disparities. (Bounds 2020)

Programs should center Indigenous voices and cultures to promote healing. Key informants... also agreed that reforms need to de-center Western models and ways of thinking and, instead, come from Indigenous ways of thinking... "We are not uplifting survivors who may have been missing. We're not building them up as the experts. Our Western way of thinking is it's best to hire someone with a degree. We should be training and helping survivors to become better experts." (Mental health provider, key informant)... "It comes down to leadership within the community. Whatever happens next has to be led by people most knowledgeable and connected and within the Indigenous community. This isn't something the state should do to them. It should come from within the community." (Researcher, key informant) (MMIW)

Seek the expertise of those with relevant lived experience to advise on all aspects of research, programs, and policies (eg, include Black DMST survivors in interpretation of findings, formulation of services) (Cook 2022)

Research and evaluation is insufficiently informed by people affected by modern slavery, including ethnic communities. Academia should partner with ethnic communities to conduct relevant research to help inform policy interventions and impactful service provision. (BASNET)

Partnerships & coalition-building

Subcategory: Interprofessional partnerships

As part of primary and secondary prevention, it is important to involve teachers and school officials, other healthcare professionals, social workers, child services agents, and those working within the legal system. The mental health profession may be best suited to develop comprehensive treatment programs that use an ecological framework to work with victims. (Salami' 2021)

When practitioners allow the treatment lens to be informed by culture, it becomes easier to foster important relationships with individuals and agencies in the community, who form the larger container informing survivor rescue and care. Providing culturally based care is critical to working in collaborative networks that include law enforcement, prosecutors, medical and social services, and the business community. (Rajan 2021)

Advocacy initiatives for the micro-level rehabilitation of sex trafficked women and girls may need to be innovative in order to transcend systemic barriers. These initiatives may… involve collaboration with various meso-level helping professionals and psycho-education for members of the judicial system in order to increase attention to the mental health needs of sex trafficked survivors. (Bryant-Davis 2017 #2)

From a public health perspective, it is particularly important to have strong collaboration among various healthcare disciplines. Organizations such as HEAL Trafficking are vital in the fight against human trafficking. This is because they not only provide advocacy and education, but they also help providers improve clinical care for trafficked persons by providing opportunities for providers to connect, access support, and identify best practices. For instance, in collaboration with an organization named Hope for Justice, HEAL Trafficking has developed a Protocol Toolkit for Developing a Response to Victims of Human Trafficking in Health Care. (Salami' 2021)

Meetings also provided opportunities for learning about other organizations' roles and existing services, improving formal and informal communication between systems and services, negotiating changes to procedures, and planning new services for sexually exploited girls. (Saewyc 2007)

The increased communication between group members, both formally and informally, is one of HYTF's most important and powerful accomplishments thus far. Relationship building between members occurred both during and between meetings. This fostered trust, increased information sharing and a greater awareness of the current systems and existing services, and facilitated connections to provide better services. One Hmong organization member reported improved connections with mainstream agencies. (Saewyc 2007)

Clinicians getting comfortable going out of their bubble and partnering with schools more. (Walker 2022)

Building positive interpersonal systems may be particularly helpful for immigrant victims... Mental health stigma and marginalization on the basis of race/ethnicity, language, immigration status, and/or religion may also limit the social resources of immigrant victims of human trafficking. Disconnection from family, friends, and other social networks can have profound effects on clients' physical and mental well-being... Mental health professionals should work with social service and legal aid agencies to help victims reconnect with loved ones from their home countries. (Salami' 2021)

Membership quickly increased through outreach, invitation by founding members, and word-of-mouth. Members came from various professions (health care, law enforcement, social services, education, judiciary) as well as roles in the represented organizations, from senior leaders to front-line workers. They brought widely differing levels of authority, talent, and experience. (Saewyc 2007)

Just as the abolition of the trans-Atlantic slave trade required movement on multiple fronts, the contemporary manifestation of human trafficking and modern-day slavery also requires a multi-sector response... Research, public service, clinical intervention, enforcement, and equal access to care, protection, and justice are key concepts to develop and sustain an effective, global anti-trafficking movement. Along with education through media and public health campaigns, modern day abolitionists must recognize the need to work toward empowerment in all of its forms, including cultural, economic, political, social, and educational. (Bryant-Davis 2017)

The high rates of human trafficking within communities of color present an excellent opportunity for professionals, researchers, and policymakers who are knowledgeable about minority health disparities to contribute their expertise to human trafficking prevention and intervention strategies. (Rollins 2017)

All participants reported that more collaboration or more effective collaboration is needed to meet girls' needs. For instance, one participant stated: "There needs to be a continuum. If I start off with 8 weeks there needs to be another 8 weeks." This participant also discussed prevention specifically: "It's preventative and proactive, so just because they're not "off the chain", as we put it, that doesn't mean that they don't need to learn how to sit right, know how to dress appropriately. They don't need to know how to keep themselves clean? They don't need to know what things they need to be focused on eating? They don't need to know other ways to exercise other than pole dance?" (Harper 2013)

The HYTF created a shared protocol for how and when to refer runaway and truant girls to various member services, and developed formal agreements for sharing information across organizations. (Saewyc 2007)

Maintain open communication and common referral protocols… within the collaborative network. (Nagy 2020)

Subcategory: Community partnership

If mainstream agencies are organizing the collaborative network, it is especially important to reach out to local First Nations, Friendship Centres, other Indigenous groups, and especially Elders, for inclusivity and perspective. (Nagy 2020)

Additionally, mental health and justice professionals should learn how to make use of cultural community leaders, religious leaders, experts, and trained interpreters to serve as consultants and assist with the provision of culturally congruent communication and support. (Bryant-Davis 2017 #2)

Collaboration among grassroots organizations, service providers, and experiential persons is key to addressing human trafficking and violence in the sex trade, including in the sharing and coordination of information, knowledges, and resources. (Nagy 2020)

Faculty must consider partnering with the Indigenous community and rely on Indigenous Elders to guide and inform practices that enhance nursing education to be responsive to diversity in health care. Embracing a strength-based approach utilizing a two-eyed vision with mutual reciprocity and respect will enhance nursing education…Nurse researchers need to partner with tribal communities employing a community participatory action iterative approach. Dissemination of findings to the tribal communities is as important as presenting in professional circles. (Peters-Mosquera et al. 2023)

Attendees concluded that a coalition had the best potential to serve the unmet needs of runaway and sexually exploited Hmong girls, because bringing together institutions and organizations that were mandated to respond to those needs, along with organizations and concerned community members who were committed to improving the lives of Hmong families and youth, could more quickly mobilize resources and make changes to institutional systems. (Saewyc 2007)

Cultural humility also includes building mutually beneficial partnerships with communities... (Ortega 2022)

This suggests that Indigenous communities and agencies in urban areas and institutions, such as policing and health care, may want to attempt working together in ways that support victims of trafficking to escape from it. (Olson-Pitawanakwat 2021)

From the beginning, membership included several Hmong organizations, and Hmong members of government and social service agencies, for example, Hmong police, probation officers, advocates, and counselors. (Saewyc 2007)

Because the HYTF included government agencies, health care, school leaders, and Hmong organizations, not all HYTF members were Hmong. It was important to evaluate how well diversity was recognized and supported, as power imbalances and polarization around racism can create problems. Most Hmong and non-Hmong members felt the group was adequately diverse, and continues to strive to ‘‘embrace diversity,’’ but acknowledged that there is always room for improvement. ‘‘As a group we are not always as culturally sensitive as we could be,’’ one participant noted. (Saewyc 2007)

Advocacy

Subcategory: Community engagement

Nurses and other professionals can foster grassroots efforts to combat public health problems at a variety of levels: initiating, leading, participating, and evaluating. Public health nurses have unique access and position within the community; they are viewed and respected as knowledgeable, approachable, trustworthy professionals that are altruistic in their actions related to creating community health and wellness. These assets and community rapport create the opportunity for coalition building, community organizing, and other effective public health strategies for mobilizing communities and systems change... As public health nurses, we should challenge ourselves to get involved in community organizing and coalition building as strategies for mobilizing communities and systems change. (Saewyc 2007)

Mezzo level… organize community stakeholders to hold public forums about mental health and substance abuse prevention; work with community stakeholders to develop comprehensive, culturally appropriate strategies to improve access to healthcare and behavioral healthcare (Valandra 2018)

...frustration and gaps in the system were clear to a number of community organizations that were involved during late 2003 in either investigating a young teen prostitution ring run by Hmong gang members, or in providing care for victims of the prostitution ring... The idea of a Task Force began with informal discussions between several eventual members at that time. The first meeting was convened in January 2004 by a social worker from the school truancy program, an attorney from the county attorney’s office, and an advanced practice nurse at the child abuse clinic. (Saewyc 2007)

Subcategory: Political advocacy

Health professionals and health systems can advocate for policies that address social determinants of health that are also systemic vulnerabilities to human trafficking such as access to housing, social, legal, and/or employment support. Health systems should also advocate for an end to discriminatory practices against immigrants and communities of color. (Prakash 2022)

Call or email your members of Congress or local elected officials to express your support for proposed policies that advance social and racial justice and public health and human rights approaches to labor exploitation or abuse of minors... Find opportunities in your professional communities (your workplace, listservs, social media, etc.) to share health equity policy demands, resources, and actions that move away from criminalization and other individual-level interventions. (Larson 2023)

Beyond creating clinical spaces that are culturally responsive, there has been a recent call to engage mental health clinicians in changing social norms and improving public policies. This work is necessary to improve the mental health of trafficked individuals, including those differentially affected by minoritized status or statuses. (Ortega 2022)

Health and social service professionals can use their expertise to be part of the solution by reducing the harms of mandatory reporting laws. These laws can remove survivor autonomy, place children outside of their families and communities, and are enforced disproportionately against communities of color. "Mitigating unnecessary harm to families and preserving the provider's role as an authentic source of help is as important as the mandate to report." Advocate for changes to definitions of neglect so that they are not synonymous with poverty. (Larson 2023)

Advocate for living wages, worker rights, and pro-labor policies that mitigate the impacts of capitalism and shift economic conditions from an individualist "free for all" to collective responsibility for the health and well-being of everyone. Support worker organizing for safety in all forms of labor (including sexual)... Addressing employment discrimination for… marginalized peoples is human trafficking prevention. (Larson 2023)

Advocate for reparations via direct cash payments to Black, Indigenous, and people of color for the generational harm historical racism, exploitation, and colonization. (Larson 2023)

Support inclusive policies that provide rights and protections for all people regardless of gender, sexual orientation, race, refugee status, or socioeconomic status. These policies should be reflected on all levels of government, from local to international, and within health facilities; (Wallace 2022)

In many respects, advocacy is an adjunctive treatment component... The American Counseling Association (ACA) has defined advocacy competencies and provided a framework for understanding the roles and responsibilities of mental health professionals who wish to engage in social justice work. These competencies highlight the importance of intervening at multiple levels: the micro-level, which refers to counselors' work with individual clients; the meso-level, which includes the client's support system or immediate community (Salami')

Macro level: support the efforts of the National Institute on Minority Health and Health Disparities (NIMHD); the National Association of Black Social Workers; the Association of Black Psychologists; and the National Alliance on Mental Illness; collaborate with the Racial and Ethnic Mental Health Disparities Coalition to reduce mental health disparities through advocacy for racial and ethnic communities (Valandra 2018)

Additionally, there is a need for individuals across professions and communities to fight for equal protection under the law for all people, and to actively work to combat the multiple forms of discrimination which promote human trafficking, particularly sexism, classism, racism, and ethnic bias. (Larson 2023)

…development of advocacy-driven community connections. Cultural humility requires… an active pursuit of accountability from systems that discriminate against multiracial and multiethnic individuals. (Davidtz 2022)

Subcategory: Supporting survivor advocacy

A Womanist ethic of therapeutic care also integrates empowerment that can translate into the opportunity for the formerly victimized to become role models and mentors for women who are currently rehabilitating their lives. (Bryant-Davis 2019)

These [resistance] strategies can be empowering for the individual and can result in social change that reduces the occurrence of oppressive acts. These resistance strategies have included marches, rallies, voting, community organizing, protests, boycotts, filing complaints, pressing charges, pursuit of civil cases, and advocacy for just policies and procedures. For trafficking victims, this can also take the form of participating in organizations that combat human trafficking, raising awareness in the public, and voting for laws that protect victims of human trafficking. (Bryant-Davis 2017)

Advocacy and community involvement may be particularly empowering for some victims. Thus, mental health professionals may wish to encourage these pursuits for clients who show interest in being active members of their community and provide support to others in need. In addition, psychological support groups for victims of trauma and human trafficking may also be suggested to build a sense of community. (Salami' 2021)

Other than creating healing-centered spaces for girls to process and heal, both several advocates and girls mentioned the need to see themselves as leaders. (Walker 2022)

Subcategory: Education

Further, rather than “screening for trafficking,” providers can educate their patients about their rights as workers and connect them to resources because many victims may not self-identify. (Prakash 2022)

Raising community awareness to prevent other young girls from running away was a key focus. The education subcommittee created a staff development video, an accompanying handout, and a comprehensive resource manual. The video included messages from school staff and local professionals, plus charts and data describing the seriousness of truancy and running away. According to one participant, ‘‘We had more data than we could put into the video . . . so we pulled that info into the resource manual, with prevention strategies, and what to look for and what to do when you see it . . . to empower them [teachers] to do as much as they can at the teaching level.’’ The video was shown to school district employees on a voluntary basis, and led to more referrals to the child abuse clinic. (Saewyc 2007)

They [health systems] can disseminate accurate information about trafficking exploitation and worker rights to counter disinformation. (Prakash 2022)

Pediatrician/medical profession [recommendations from advocates]: ➔ Educational seminars with girls (Walker 2022)
